# Correction: The Children’s Hospitals in Africa Mapping Project (CHAMP) survey: Facilities, equipment, supplies, infrastructure, and capacity to respond to emergencies

**DOI:** 10.1371/journal.pgph.0005805

**Published:** 2026-01-06

**Authors:** Vinayak Bhardwaj, Lawrence R. Stanberry, Philip LaRussa, Wilmot James, Maitry Mahida, Aimable Kanyamuhunga, Atnafu Mekonnen Tekleab, Augustine Omoigberale, Crispen Ngwenya, David Musorewegomo, Dipesalema Joel, Ezekiel Mupere, Fidelis Ewenitie Eki-Udoko, Hannah Bousquet, Heloise Buys, Hilda Angela Mujuru, Ike Oluwa Lagunju, Irene Marete, Jethro Zawolo, Jonathan Kaunda Mwansa, Joseph Tawanda Chava, Maima Kawah Baysah, Mildred Anyango Mudany, Nancy Biyeah Yang Ngum, Nellie V. T. Bell, One Bayani, Pauline Samia, Ruth Nduati, Sam Miti, Schyler Zane Grodman, Thembisile Dintle Mosalakatane, Workeabeba Abebe, Ashraf Coovadia

In the Funding section, the funder’s name is incorrect. The correct funder is: ELMA Foundation (institutional grant to PL; WJ; and LS).
