# Supplementary material for: The Children’s Hospitals in Africa Mapping Project (CHAMP) survey: Facilities, equipment, supplies, infrastructure, and capacity to respond to emergencies
Source: PLOS Glob Public Health. 2025 Nov 26;5(11):e0005153. doi: 10.1371/journal.pgph.0005153 (PMC12654909; doi:10.1371/journal.pgph.0005153)
Supplement: S1 Appendix — (DOCX) [file pgph.0005153.s001.docx]

S1 Appendix: CHAMP RedCap Survey Tool

# Variable / Field Name Field Label

***Field Note***

Instrument: Identification (identification)  Enabled as survey

Field Attributes (Field Type, Validation, Choices, Calculations, etc.)

1. [ record_id ]
2. [ glossary ]
3. [ instituion_name ]
4. [ location ]
5. [ keep_track ]
6. [ identification_complete ]

Record ID

Attached is a glossary of terms and list of abbreviations used throughout the survey. Please download for use as a reference during the rest of the survey.

Name of institution

Location (City/Province/Region/Country)

Please keep track of when you start and complete the survey, the amount of time spent on the survey, and the other people who helped you complete it. We will request this information in the final "Summary and Suggestions" portion of the survey.

Section Header: *Form Status*

Complete?

text descriptive

text text

descriptive

dropdown

| 0 | Incomplete |
| --- | --- |
| 1 | Unverified |
| 2 | Complete |

Instrument: Background (background)  Enabled as survey

1. [ background ]
2. [ facility_designation ]
3. [ facility_designation_other ]

Show the field ONLY if: [facility_designation] = '4'

1. [ facility_designation_123 ]
2. [ facility_public_or_private ]
3. [ terminology ]
4. [ medical_school_affiliated ]
5. [ medical_school_name ]

Show the field ONLY if: [medical_school_affiliated] = '1'

This is a space to give background information on your hospital. *Examples of topics for comment: If your hospital is part of a network, you might comment on how your hospital its into the larger system. If your facility is a mother-and-child hospital or if infants born in another hospital in your network are treated by the same set of doctors but remain at the maternal hospital.*

Section Header: *Type of facility*

What is the designation of your facility?

Please specify "other"

Are you a primary, secondary, or tertiary hospital?

Is your facility public (government), private or a public/private partnership

You may use this box to describe your hospital using your country's terminology (e.g. specialized-regional)

*optional*

Is you hospital affiliated with a medical school?

Name of medical school with which hospital is affiliated and city where it is located.

notes

radio

| 1 | National |
| --- | --- |
| 2 | Regional |
| 3 | District |
| 4 | Other |

text

radio

| 1 | Primary |
| --- | --- |
| 2 | Secondary |
| 3 | Tertiary |

radio

| 1 | public (government) |
| --- | --- |
| 2 | private |
| 3 | public/private partnership |

text

yesno

| 1 | Yes |
| --- | --- |
| 0 | No |

text

| 15 | [ refer_info ] | Section Header: *Network*  The following questions ask about the hospitals and clinics from which you typically/officially accept pediatric patients (hospitals/clinics that up-refer to your institution).  *The term "hospital" will be used for the remaining questions in this section, but this term also includes clinics.*  You may list UP TO 6 hospitals (you do not need to list all 6 and may leave the unused slots blank). Please list the hospitals in order of how many patients they refer to your institution with the hospitals from which you accept the most patients listed first. | descriptive | | |
| --- | --- | --- | --- | --- | --- |
| 16 | [ refer_name_1 ] | Name of Hospital 1 from which you accept referrals | text | | |
| 17 | [ refer_dist_1 ] | What is the approximate distance of Hospital 1 from your institution? | radio | | |
|  |  |  | 1 | 0-5 km |  |
|  |  |  | 2 | 6-10 km |  |
|  |  |  | 3 | 11-20 km |  |
|  |  |  | 4 | 21-50 km |  |
|  |  |  | 5 | 50+ km |  |
| 18 | [ refer_name_2 ]  Show the field ONLY if: [refer_name_1] <> '' | Name of Hospital 2 from which you accept referrals | text | | |
| 19 | [ refer_dist_2 ]  Show the field ONLY if: [refer_name_1] <> '' | What is the approximate distance of Hospital 2 from your institution? | radio | | |
|  |  |  | 1 | 0-5 km |  |
|  |  |  | 2 | 6-10 km |  |
|  |  |  | 3 | 11-20 km |  |
|  |  |  | 4 | 21-50 km |  |
|  |  |  | 5 | 50+ km |  |
| 20 | [ refer_name_3 ]  Show the field ONLY if: [refer_name_2] <> '' | Name of Hospital 3 from which you accept referrals | text | | |
| 21 | [ refer_dist_3 ]  Show the field ONLY if: [refer_name_2] <> '' | What is the approximate distance of Hospital 3 from your institution? | radio | | |
|  |  |  | 1 | 0-5 km |  |
|  |  |  | 2 | 6-10 km |  |
|  |  |  | 3 | 11-20 km |  |
|  |  |  | 4 | 21-50 km |  |
|  |  |  | 5 | 50+ km |  |
| 22 | [ refer_name_4 ]  Show the field ONLY if: [refer_name_3] <> '' | Name of Hospital 4 from which you accept referrals | text | | |
| 23 | [ refer_dist_4 ]  Show the field ONLY if: [refer_name_3] <> '' | What is the approximate distance of Hospital 4 from your institution? | radio | | |
|  |  |  | 1 | 0-5 km |  |
|  |  |  | 2 | 6-10 km |  |
|  |  |  | 3 | 11-20 km |  |
|  |  |  | 4 | 21-50 km |  |
|  |  |  | 5 | 50+ km |  |
| 24 | [ refer_name_5 ]  Show the field ONLY if: [refer_name_4] <> '' | Name of Hospital 5 from which you accept referrals | text | | |

| 25 | [ refer_dist_5 ]  Show the field ONLY if: [refer_name_4] <> '' | What is the approximate distance of Hospital 5 from your institution? | radio | | | | |
| --- | --- | --- | --- | --- | --- | --- | --- |
|  |  |  | 1 | 0-5 km | |  | |
|  |  |  | 2 | 6-10 km | |  |  |
|  |  |  | 3 | 11-20 km | |  |  |
|  |  |  | 4 | 21-50 km | |  |  |
|  |  |  | 5 | 50+ km | |  |  |
| 26 | [ refer_name_6 ]  Show the field ONLY if: [refer_name_5] <> '' | Name of Hospital 6 from which you accept referrals | text | | | | |
| 27 | [ refer_dist_6 ]  Show the field ONLY if: [refer_name_5] <> '' | What is the approximate distance of Hospital 6 from your institution? | radio | | | | |
|  |  |  | 1 | 0-5 km | |  | |
|  |  |  | 2 | 6-10 km | |  |  |
|  |  |  | 3 | 11-20 km | |  |  |
|  |  |  | 4 | 21-50 km | |  |  |
|  |  |  | 5 | 50+ km | |  |  |
| 28 | [ other_referring ] | Are there other hospitals that refer large numbers of patients to you? | yesno | |  | | |
|  |  |  | 1 | Yes |  |  |  |
|  |  |  | 0 | No |  |  |  |
| 29 | [ other_referral_mech ] | What are the other referral mechanisms beyond official ones from earlier questions?  *e.g. referrals from private hospitals, self-referral, other (please specify)* | notes | | | | |
| 30 | [ annual_patients_accepted ] | How many pediatric patients are accepted annually from referring hospitals and clinics for inpatient services?  *If the number is unknown please write "unknown"* | text | | | | |
| 31 | [ twoweek_accepted_patients ]  Show the field ONLY if: [annual_patients_accepted] = 'unknown' or [annual_patients  _accepted] = 'Unknown' or [an nual_patients_accepted] = '"un  known"' | If the number of pediatric patients accepted annually is unknown, please give us your best estimate for the number of patients accepted from referring hospitals and clinics in the past 2 weeks. | text | | | | |
| 32 | [ annual_patients_accept_est ]  Show the field ONLY if: [twoweek_accepted_patients]  > 0 | Estimate for number of pediatric patients accepted annually through referrals: | calc  Calculation: [twoweek_accepted_patients]*26 | | | | |
| 33 | [ up_refer ] | Do you ever up-refer pediatric patients? | radio | | | | |
|  |  |  | 0 | No | | |  |
|  |  |  | 1 | Yes - including other hospitals within the country | | |  |
|  |  |  | 2 | Yes - only internationally | | |  |
| 34 | [ refer_info_2 ]  Show the field ONLY if: [up_refer] = '1' or [up_refer] = '2' | The following questions ask about the hospitals to which you up- refer pediatric patients.  You may list UP TO 4 hospitals (you do not need to list all 4 and may leave the unused slots blank). Please list the hospitals in order of how many patients your refer to them with the hospitals to which you refer the most patients listed first. | descriptive | | | | |
| 35 | [ uprefer_name_1 ]  Show the field ONLY if: [up_refer] = '1' or [up_refer] = '2' | Name of Hospital 1 to which you up-refer pediatric patients | text | | | | |
| 36 | [ uprefer_for_1 ]  Show the field ONLY if: [up_refer] = '1' or [up_refer] = '2' | Do you only refer to Hospital 1 for specific types of cases (e.g. radiotherapy, orthopedic surgery)?  If yes, please specify. Otherwise you may leave this blank. | text | | | | |

| 37 | [ uprefer_dist_1 ]  Show the field ONLY if: [up_refer] = '1' or [up_refer] = '2' | What is the approximate distance of Hospital 1 from your institution? | radio | | |
| --- | --- | --- | --- | --- | --- |
|  |  |  | 1 | 0-5 km |  |
|  |  |  | 2 | 6-10 km |  |
|  |  |  | 3 | 11-20 km |  |
|  |  |  | 4 | 21-50 km |  |
|  |  |  | 5 | 50+ km |  |
| 38 | [ uprefer_name_2 ]  Show the field ONLY if: [uprefer_name_1] <> '' | Name of Hospital 2 to which you up-refer pediatric patients | text | | |
| 39 | [ uprefer_for_2 ]  Show the field ONLY if: [uprefer_name_1] <> '' | Do you only refer to Hospital 2 for specific types of cases (e.g. radiotherapy, orthopedic surgery)?  If yes, please specify. Otherwise you may leave this blank. | text | | |
| 40 | [ uprefer_dist_2 ]  Show the field ONLY if: [uprefer_name_1] <> '' | What is the approximate distance of Hospital 2 from your institution? | radio | | |
|  |  |  | 1 | 0-5 km |  |
|  |  |  | 2 | 6-10 km |  |
|  |  |  | 3 | 11-20 km |  |
|  |  |  | 4 | 21-50 km |  |
|  |  |  | 5 | 50+ km |  |
| 41 | [ uprefer_name_3 ]  Show the field ONLY if: [uprefer_name_2] <> '' | Name of Hospital 3 to which you up-refer pediatric patients | text | | |
| 42 | [ uprefer_for_3 ]  Show the field ONLY if: [uprefer_name_2] <> '' | Do you only refer to Hospital 3 for specific types of cases (e.g. radiotherapy, orthopedic surgery)?  If yes, please specify. Otherwise you may leave this blank. | text | | |
| 43 | [ uprefer_dist_3 ]  Show the field ONLY if: [uprefer_name_2] <> '' | What is the approximate distance of Hospital 3 from your institution? | radio | | |
|  |  |  | 1 | 0-5 km |  |
|  |  |  | 2 | 6-10 km |  |
|  |  |  | 3 | 11-20 km |  |
|  |  |  | 4 | 21-50 km |  |
|  |  |  | 5 | 50+ km |  |
| 44 | [ uprefer_name_4 ]  Show the field ONLY if: [uprefer_name_3] <> '' | Name of Hospital 4 to which you up-refer pediatric patients | text | | |
| 45 | [ uprefer_for_4 ]  Show the field ONLY if: [uprefer_name_3] <> '' | Do you only refer to Hospital 4 for specific types of cases (e.g. radiotherapy, orthopedic surgery)?  If yes, please specify. Otherwise you may leave this blank. | text | | |
| 46 | [ uprefer_dist_4 ]  Show the field ONLY if: [uprefer_name_3] <> '' | What is the approximate distance of Hospital 4 from your institution? | radio | | |
|  |  |  | 1 | 0-5 km |  |
|  |  |  | 2 | 6-10 km |  |
|  |  |  | 3 | 11-20 km |  |
|  |  |  | 4 | 21-50 km |  |
|  |  |  | 5 | 50+ km |  |
| 47 | [ annual_patients_referred ]  Show the field ONLY if: [up_refer] = '1' or [up_refer] = '2' | How many pediatric patients do you up-refer annually?  *If the number is unknown please write "unknown"* | text | | |
| 48 | [ twoweek_refer_patients ]  Show the field ONLY if: [annual_patients_referred] = 'u nknown' or [annual_patients_r eferred] = 'Unknown' or [annu al_patients_referred] = '"unkno wn"' | If the number of patients referred annually is unknown, please give us your best estimate for the number of patients up-referred in the past 2 weeks. | text | | |

1. [ annual_patients_refer_est ]

Show the field ONLY if: [twoweek_refer_patients] > 0

1. [ free_standing ]
2. [ separate_ward ]

Show the field ONLY if: [free_standing] = '0'

1. [ no_separate_ward ]

Show the field ONLY if: [separate_ward] = '0'

1. [ child_max_age ]
2. [ adult_admit ]
3. [ dif_age_range ]
4. [ dif_age_range_yes ]

Show the field ONLY if: [dif_age_range] = '1'

1. [ sep_ward_adolescents ]
2. [ adolescent_housed ] Show the field ONLY if:

[sep_ward_adolescents] = '1'

Estimate for number of patients up-referred annually:

Section Header: *Pediatrics*

Is your facility a free-standing children's hospital?

If not a free-standing children's hospital, is there a separate ward/facility to care for children?

If no, what other spaces are used to care for children?

What is the maximum age of children you care for in your ward/facility?

At what age are patients admitted to the adult ward?

Is there a difference in the age range for acute sick children and those who are followed at chronic diseases clinics? (e.g. diabetic, HIV, cardiac)

If yes, please explain:

Is there a separate ward/facility for adolescents (i.e. age 10-19 years)?

If yes, is this facility under pediatrics or in the adult hospital?

calc

Calculation: [twoweek_refer_patients]*26

yesno

1 Yes

1. No

yesno

1. Yes
2. No text

text (integer, Min: 0, Max: 30)

Custom alignment: RH

text (integer, Min: 0, Max: 30) Custom alignment: RH

yesno

1. Yes
2. No notes

yesno

1. Yes
2. No

checkbox

1. adolescent_housed 1 Pediatric
2. adolescent_housed 2 Adult
3. [ adolescent_housed_other ]

Show the field ONLY if: [adolescent_housed(3)] = '1'

If you selected "other," please specify

3

text

adolescent_housed 3

Other

1. [ background_clarify ]
2. [ background_complete ]

Section Header: *Clariications*

This is a space to clarify any answers from this section.

Section Header: *Form Status*

notes

dropdown

Complete?

Instrument: Departments (departments)  Enabled as survey

1. Incomplete
2. Unverified
3. Complete
4. [ departments_background ]
5. [ sep_ped_inpatient ]
6. [ n_inpatient_beds ]
7. [ avg_daily_census_inpatient ]
8. [ avg_bed_occupancy_inpat ]

This is a space to give background information on the inpatient areas, intensive care unit, outpatient department, and emergency (casualty) room setup in your hospital.

Section Header: *Inpatient (these questions all refer to pediatric inpatients)*

Do you have a separate pediatric inpatient area?

Total number of pediatric inpatient beds (using definition of pediatric patient in your institution, and assuming that you have access to a full complement of nurses, doctors, and other staff to care for these children)

What is the average daily census for pediatric inpatients of any kind?

What is the average bed occupancy rate?

*(as a percent)*

notes

yesno

1 Yes

0 No

text (integer)

text

text (integer, Min: 1, Max: 100)

| 1 | Yes |
| --- | --- |
| 0 | No |

| 1 | ideal_inpat_beds_why_not 1 | Staffing (number of nurses) |
| --- | --- | --- |
| 2 | ideal_inpat_beds_why_not 2 | Staffing (number of doctors) |
| 3 | ideal_inpat_beds_why_not 3 | Finances |
| 4 | ideal_inpat_beds_why_not 4 | Space |
| 5 | ideal_inpat_beds_why_not 5 | Other |

| 1 | Yes |
| --- | --- |
| 0 | No |

| 1 | daily |
| --- | --- |
| 2 | weekly |
| 3 | monthly |
| 4 | seasonally (e.g. malaria season) |
| 5 | rarely |

| 67 | [ high_month_census_inpat ] | What was your highest monthly census for pediatric inpatients of any kind in the past year? | text |
| --- | --- | --- | --- |
| 68 | [ high_month_inpat_reason ] | What was the primary reason for the high month census?  *(e.g. malaria)* | text |
| 69 | [ full_capacity ] | How many times have you reached 100% capacity in the past year? | text |
| 70 | [ adq_inpat_beds ] | Do you have an adequate number of pediatric inpatient beds to meet current needs according to your current criteria for admission? | yesno |
| 71 | [ addl_inpat_beds ]  Show the field ONLY if: [adq_inpat_beds] = '0' | If no, how many additional beds are needed for pediatric inpatients? | text |
| 72 | [ addl_inpat_beds_by_ward ]  Show the field ONLY if: [adq_inpat_beds] = '0' | Are additional beds needed in particular wards? If yes, please specify. | text |
| 73 | [ ideal_inpat_beds ] | What would be the ideal number of additional beds for pediatric inpatients? (does not necessarily have to follow current admissions criteria)  For this question assume that staffing, space, finances, etc. are not an issue - i.e. consider only the need.  *If current number of beds is optimal, enter "0"* | text |
| 74 | [ ideal_inpat_bed_use ]  Show the field ONLY if: [ideal_inpat_beds] > 0 | How would you use these additional beds?  *(e.g. would open 25 additional beds in the nursery to keep more babies under observation or in general pediatrics to admit more children with malaria, bronchiolitis, diarrhea, etc.)* | notes |
| 75 | [ ideal_inpat_beds_why_not ]  Show the field ONLY if: [ideal_inpat_beds] > 0 | What would prevent you from adding the additional beds?  *please identify the MAIN reason(s)* | checkbox |
| 76 | [ ideal_beds_why_not_other ]  Show the field ONLY if: [ideal_inpat_beds_why_not(5)]  = '1' | If you selected other, please specify | text |
| 77 | [ mult_pat_per_bed ] | Is it ever necessary to put more than one child in a bed? | yesno |
| 78 | [ mult_pat_per_bed_freq ]  Show the field ONLY if: [mult_pat_per_bed] = '1' | If yes, how frequently is it necessary to put more than one child in a bed? | radio |
| 79 | [ separated_wards ] | If there are multiple (non-ICU) pediatric wards  (i.e. separated by age, disease, public vs. private, etc.) please list the different wards. If there is only one ward, please indicate this in the comments, otherwise, indicate age ranges, disease classifications, etc.  *e.g. ward for age 0-1 month, surgical ward, pediatric oncology ward* | notes |
| 80 | [ n_inpat_isolation_rms ] | Number of dedicated isolation rooms for pediatric inpatients. (Isolation room refers to a separate room, not just a particular bed at one end of the ward with a curtain around it). | text (integer) |

1. [ inpat_cohort ]
2. [ isolation_sop ]
3. [ isolation_sop_attach ]

Show the field ONLY if: [isolation_sop] = '1'

1. [ isolation_sop_attach_2 ]

Show the field ONLY if: [isolation_sop_attach] <> ''

1. [ isolation_sop_attach_3 ]

Show the field ONLY if: [isolation_sop_attach_2] <> ''

1. [ isolation_sop_url ]

Show the field ONLY if: [isolation_sop] = '1'

1. [ isolation_sop_url_2 ]

Show the field ONLY if: [isolation_sop_url] <> ''

1. [ isolation_sop_url_3 ]

Show the field ONLY if: [isolation_sop_url_2] <> ''

1. [ inpat_surge ]
2. [ inpat_surge_how_many ]

Show the field ONLY if: [inpat_surge] = '1'

1. [ inpat_surge_where ]

Show the field ONLY if: [inpat_surge] = '1'

1. [ inpat_surge_yes ]

Show the field ONLY if: [inpat_surge] = '1'

1. [ inpat_surge_no ]

Show the field ONLY if: [inpat_surge] = '0'

1. [ inpat_surge_no_yes ]

Show the field ONLY if: [inpat_surge_no] = '1'

1. [ inpat_surge_no_yes_2 ]

Show the field ONLY if: [inpat_surge_no] = '1'

1. [ inpatient_clarify ]
2. [ high_care_units ]
3. [ high_care_units_explain ]

Show the field ONLY if: [high_care_units] = '1'

In an infectious disease emergency, do you have the capacity to cohort patients (i.e. you put all of the children with bronchiolitis on one ward)?

Do you have an SOP on isolation and/or cohorting?

If yes, please attach the document here. If SOP is available online, insert the URL below.

Additional file

Additional file

Paste the URL here:

Additional URL

Additional URL

In a catastrophic event that overwhelms normal capacity (e.g. Cholera, chemical poisoning), are you able to accept additional pediatric patients above your usual maximum capacity?

If yes, how many?

If yes, to which unit(s) would additional inpatients be admitted?

If yes, how do you accommodate the excess patients?

*(e.g. more beds are put in the ward, double up in each bed, put patients on the loor, etc.)*

If no, is there pediatric inpatient surge capacity elsewhere?

If yes, where?

If yes, how many additional pediatric patients can be admitted?

This is a space to clarify any answers from the inpatient section.

Section Header: *High-care/Intermediary/Transitional care (not ICU)*

Does your facility have a unit (or units) for pediatric patients in addition to the ICU and general wards? (e.g. high- care/intermediary/transitional care).

If yes, please explain.

yesno

1. Yes
2. No

yesno

1. Yes
2. No file

file

file

text

text

text

yesno

1. Yes
2. No

text (integer)

notes

notes

yesno

1. Yes
2. No text

text

notes

yesno

1. Yes
2. No notes

| 99 | [ n_high_care_units ]  Show the field ONLY if: [high_care_units] = '1' | How many such units are there? | text (integer) | | | |
| --- | --- | --- | --- | --- | --- | --- |
| 100 | [ first_high_care_unit ]  Show the field ONLY if: [n_high_care_units] > 1 | Name of first unit described | text | | | |
| 101 | [ high_care_units_use ]  Show the field ONLY if: [high_care_units] = '1' | What is that unit used for? | notes | | | |
| 102 | [ high_care_units_beds ]  Show the field ONLY if: [high_care_units] = '1' | Number of beds in that unit | text (integer) | | | |
| 103 | [ high_care_units_census ]  Show the field ONLY if: [high_care_units] = '1' | What is the average daily census in that unit? | text (integer) | | | |
| 104 | [ high_care_units_occup ]  Show the field ONLY if: [high_care_units] = '1' | What is the bed occupancy rate in that unit?  *as a percent* | text (integer, Min: 1, Max: 100) | | | |
| 105 | [ high_care_units_adq ]  Show the field ONLY if: [high_care_units] = '1' | Do you have an adequate number of beds in that unit to meet current needs? | yesno | |  | |
|  |  |  | 1 | Yes |  |  |
|  |  |  | 0 | No |  |  |
| 106 | [ high_care_units_addl ]  Show the field ONLY if: [high_care_units_adq] = '0' | If no, how many additional beds are needed? | text (integer) | | | |
| 107 | [ high_care_units_addl_y_not ]  Show the field ONLY if: [high_care_units_adq] = '0' | What would prevent you from adding the additional beds?  *please identify the MAIN reason(s)* | checkbox | | | |
|  |  |  | 1 | high_care_units_addl_y_not 1 | | Staffing (number of nurses) |
|  |  |  | 2 | high_care_units_addl_y_not 2 | | Staffing (number of doctors) |
|  |  |  | 3 | high_care_units_addl_y_not 3 | | Finances |
|  |  |  | 4 | high_care_units_addl_y_not 4 | | Space |
|  |  |  | 5 | high_care_units_addl_y_not 5 | | Other |
| 108 | [ hcu_addl_y_not_other ]  Show the field ONLY if: [high_care_units_addl_y_not (5)] = '1' | If you selected other, please specify | text | | | |
| 109 | [ high_care_units_use_2 ]  Show the field ONLY if: [n_high_care_units] > 1 | What is that unit used for? | notes | | | |
| 110 | [ high_care_units_beds_2 ]  Show the field ONLY if: [n_high_care_units] > 1 | Number of beds in that unit | text (integer) | | | |
| 111 | [ high_care_units_census_2 ]  Show the field ONLY if: [n_high_care_units] > 1 | What is the average daily census in that unit? | text (integer) | | | |
| 112 | [ high_care_units_occup_2 ]  Show the field ONLY if: [n_high_care_units] > 1 | What is the bed occupancy rate in that unit?  *as a percent* | text (integer, Min: 1, Max: 100) | | | |
| 113 | [ high_care_units_adq_2 ]  Show the field ONLY if: [n_high_care_units] > 1 | Do you have an adequate number of beds in that unit to meet current needs? | yesno | |  | |
|  |  |  | 1 | Yes |  |  |
|  |  |  | 0 | No |  |  |
| 114 | [ high_care_units_addl_2 ]  Show the field ONLY if: [high_care_units_adq_2] = '0' | If no, how many additional beds are needed? | text (integer) | | | |

| 115 | [ high_care_units_addl_y_not_2  ]  Show the field ONLY if: [high_care_units_adq_2] = '0' | What would prevent you from adding the additional beds?  *please identify the MAIN reason(s)* | checkbox | | | |
| --- | --- | --- | --- | --- | --- | --- |
|  |  |  | 1 | high_care_units_addl_y_not_2 1 | | Staffing (number of nurses) |
|  |  |  | 2 | high_care_units_addl_y_not_2 2 | | Staffing (number of doctors) |
|  |  |  | 3 | high_care_units_addl_y_not_2 3 | | Finances |
|  |  |  | 4 | high_care_units_addl_y_not_2 4 | | Space |
|  |  |  | 5 | high_care_units_addl_y_not_2 5 | | Other |
| 116 | [ hcu_addl_y_not_other_2 ]  Show the field ONLY if: [high_care_units_addl_y_not_2 (5)] = '1' | If you selected other, please specify | text | | | |
| 117 | [ first_high_care_unit_3 ]  Show the field ONLY if: [n_high_care_units] > 2 | Name of third unit described | text | | | |
| 118 | [ high_care_units_use_3 ]  Show the field ONLY if: [n_high_care_units] > 2 | What is that unit used for? | notes | | | |
| 119 | [ high_care_units_beds_3 ]  Show the field ONLY if: [n_high_care_units] > 2 | Number of beds in that unit | text (integer) | | | |
| 120 | [ high_care_units_census_3 ]  Show the field ONLY if: [n_high_care_units] > 2 | What is the average daily census in that unit? | text (integer) | | | |
| 121 | [ high_care_units_occup_3 ]  Show the field ONLY if: [n_high_care_units] > 2 | What is the bed occupancy rate in that unit?  *as a percent* | text (integer, Min: 1, Max: 100) | | | |
| 122 | [ high_care_units_adq_3 ]  Show the field ONLY if: [n_high_care_units] > 2 | Do you have an adequate number of beds in that unit to meet current needs? | yesno | |  | |
|  |  |  | 1 | Yes |  |  |
|  |  |  | 0 | No |  |  |
| 123 | [ high_care_units_addl_3 ]  Show the field ONLY if: [high_care_units_adq_3] = '0' | If no, how many additional beds are needed? | text (integer) | | | |
| 124 | [ high_care_units_addl_y_not_3  ]  Show the field ONLY if: [high_care_units_adq_3] = '0' | What would prevent you from adding the additional beds?  *please identify the MAIN reason(s)* | checkbox | | | |
|  |  |  | 1 | high_care_units_addl_y_not_3 1 | | Staffing (number of nurses) |
|  |  |  | 2 | high_care_units_addl_y_not_3 2 | | Staffing (number of doctors) |
|  |  |  | 3 | high_care_units_addl_y_not_3 3 | | Finances |
|  |  |  | 4 | high_care_units_addl_y_not_3 4 | | Space |
|  |  |  | 5 | high_care_units_addl_y_not_3 5 | | Other |
| 125 | [ hcu_addl_y_not_other_3 ]  Show the field ONLY if: [high_care_units_addl_y_not_3 (5)] = '1' | If you selected other, please specify | text | | | |
| 126 | [ high_care_clarify ] | This is a space to clarify any answers from the high-care section. | notes | | | |
| 127 | [ icu ] | Section Header: *Intensive Care Units (ICU)*  Does your facility have an ICU where children are cared for? | yesno | |  | |
|  |  |  | 1 | Yes |  |  |
|  |  |  | 0 | No |  |  |

| 128 | [ icu_types ] | Which of the following best describe the ICU(s)  *check all that apply* | checkbox | | | | |
| --- | --- | --- | --- | --- | --- | --- | --- |
|  |  |  | 1 | icu_types 1 | | Adult ICU that is also used for children | |
|  |  |  | 2 | icu_types 2 | | Separate pediatric ICU (PICU) | |
|  |  |  | 3 | icu_types 3 | | Separate neonatal ICU (NICU) | |
|  |  |  | 4 | icu_types 4 | | Combined NICU/PICU | |
| 129 | [ adult_icu_beds ]  Show the field ONLY if: [icu_types(1)] = '1' | Section Header: *If you have an adult ICU where children are also cared for:*  How many beds are available for use for pediatric patients? | text | | | | |
| 130 | [ adult_icu_dedicated ]  Show the field ONLY if: [icu_types(1)] = '1' | Are there dedicated pediatric beds in the ICU? | yesno | |  | | |
|  |  |  | 1 | Yes |  |  |  |
|  |  |  | 0 | No |  |  |  |
| 131 | [ adult_icu_n_dedicated ]  Show the field ONLY if: [adult_icu_dedicated] = '1' | If yes, how many dedicated pediatric beds are there in the ICU? | text | | | | |
| 132 | [ adult_icu_adc ]  Show the field ONLY if: [icu_types(1)] = '1' | What is the average daily census of pediatric patients in the ICU? | text | | | | |
| 133 | [ adult_icu_bor ]  Show the field ONLY if: [icu_types(1)] = '1' | What is the average bed occupancy rate in the ICU?  *as a percent* | text (integer, Min: 1, Max: 100) | | | | |
| 134 | [ adult_icu_adq_beds ]  Show the field ONLY if: [icu_types(1)] = '1' | Do you have an adequate number of beds in the ICU to meet current pediatric needs? | yesno | |  | | |
|  |  |  | 1 | Yes |  |  |  |
|  |  |  | 0 | No |  |  |  |
| 135 | [ adult_icu_addl_beds ]  Show the field ONLY if: [adult_icu_adq_beds] = '0' | If no, how many additional beds are needed for pediatric patients in the ICU? | text | | | | |
| 136 | [ adult_icu_addl_y_not ]  Show the field ONLY if: [adult_icu_adq_beds] = '0' | What would prevent you from adding the additional beds?  *please identify the MAIN reason(s)* | checkbox | | | | |
|  |  |  | 1 | adult_icu_addl_y_not 1 | | | Staffing (number of nurses) |
|  |  |  | 2 | adult_icu_addl_y_not 2 | | | Staffing (number of doctors) |
|  |  |  | 3 | adult_icu_addl_y_not 3 | | | Finances |
|  |  |  | 4 | adult_icu_addl_y_not 4 | | | Space |
|  |  |  | 5 | adult_icu_addl_y_not 5 | | | Other |
| 137 | [ adult_icu_addl_y_not_other ]  Show the field ONLY if: [adult_icu_addl_y_not(5)] = '1' | If you selected other, please specify | text | | | | |

| 138 | [ icu_services ]  Show the field ONLY if: [icu_types(1)] = '1' | Which of the following are available for pediatric patients in ICU beds? | checkbox | | | |
| --- | --- | --- | --- | --- | --- | --- |
|  |  |  | 1 | icu_services 1 | | Pulse oximetry |
|  |  |  | 2 | icu_services 2 | | Continuous ECG |
|  |  |  | 3 | icu_services 3 | | Invasive pressure monitoring |
|  |  |  | 4 | icu_services 4 | | Mechanical ventilators |
|  |  |  | 5 | icu_services 5 | | CPAP |
|  |  |  | 6 | icu_services 6 | | Oxygen concentrators |
|  |  |  | 7 | icu_services 7 | | Oxygen cylinders |
|  |  |  | 8 | icu_services 8 | | Peritoneal dialysis |
|  |  |  | 9 | icu_services 9 | | Hemodialysis |
|  |  |  | 10 | icu_services 10 | | Cooling devices for induced hypothermia |
|  |  |  | 11 | icu_services 11 | | High frequency oscillators (HFVO) |
|  |  |  | 12 | icu_services 12 | | EEG monitors (Brainz monitors) |
|  |  |  | 13 | icu_services 13 | | Ultrasound imaging in the ICU |
|  |  |  | 14 | icu_services 14 | | Phototherapy |
|  |  |  | 15 | icu_services 15 | | Central lines |
| 139 | [ adult_icu_ventilators ]  Show the field ONLY if: [icu_services(4)] = '1' | How many mechanical ventilators do you have in the ICU? | text (integer) | | | |
| 140 | [ adult_icu_funct_vent ]  Show the field ONLY if: [icu_services(4)] = '1' | How many mechanical ventilator units are functional on a given day? (i.e. may or may not be in use, but could be used if necessary) | text (integer) | | | |
| 141 | [ adult_icu_cpap ]  Show the field ONLY if: [icu_services(5)] = '1' | How many CPAP units do you have in the ICU? | text (integer) | | | |
| 142 | [ adult_icu_funct_cpap ]  Show the field ONLY if: [icu_services(5)] = '1' | How many CPAP units are functional on a given day? | text (integer) | | | |
| 143 | [ adult_icu_o2_conc ]  Show the field ONLY if: [icu_services(6)] = '1' | How many oxygen concentrators do you have in the ICU? | text (integer) | | | |
| 144 | [ adult_icu_funct_o2_conc ]  Show the field ONLY if: [icu_services(6)] = '1' | How many oxygen concentrator units are functional on a given day? | text (integer) | | | |
| 145 | [ adult_icu_o2_cylinders ]  Show the field ONLY if: [icu_services(7)] = '1' | How many oxygen cylinders do you have in the ICU? | text (integer) | | | |
| 146 | [ adult_icu_surge ]  Show the field ONLY if: [icu_types(1)] = '1' | Do you have surge capacity in the adult ICU for pediatric patients? | yesno | |  | |
|  |  |  | 1 | Yes |  |  |
|  |  |  | 0 | No |  |  |
| 147 | [ adult_icu_surge_yes ]  Show the field ONLY if: [adult_icu_surge] = '1' | If yes, how many additional pediatric ICU patients could be admitted to your adult ICU? | text | | | |
| 148 | [ adult_icu_surge_no ]  Show the field ONLY if: [adult_icu_surge] = '0' | If no, is there pediatric ICU surge capacity elsewhere? | yesno | |  | |
|  |  |  | 1 | Yes |  |  |
|  |  |  | 0 | No |  |  |
| 149 | [ adult_icu_surge_where ]  Show the field ONLY if: [adult_icu_surge_no] = '1' | If yes, where? | text | | | |

150

151

152

153

154

155

156

157

158

159

160

161

162

163

164

[ adult_icu_surge_other_n ]

Show the field ONLY if: [adult_icu_surge_no] = '1'

[ adult_icu_isolation ]

Show the field ONLY if: [icu_types(1)] = '1'

[ adult_icu_n_isolation ]

Show the field ONLY if: [adult_icu_isolation] = '1'

[ adult_icu_n_beds_iso ]

Show the field ONLY if: [adult_icu_isolation] = '1'

[ adult_icu_cohort ]

Show the field ONLY if: [icu_types(1)] = '1'

[ adult_icu_cohort_n ]

Show the field ONLY if: [adult_icu_cohort] = '1'

[ adult_icu_cohort_no ]

Show the field ONLY if: [adult_icu_cohort] = '0'

[ adult_icu_cohort_where ]

Show the field ONLY if: [adult_icu_cohort_no] = '1'

[ adult_icu_cohort_no_n ]

Show the field ONLY if: [adult_icu_cohort_no] = '1'

[ picu_n_beds ]

Show the field ONLY if: [icu_types(2)] = '1'

[ picu_adc ]

Show the field ONLY if: [icu_types(2)] = '1'

[ picu_bor ]

Show the field ONLY if: [icu_types(2)] = '1'

[ picu_adq_beds ]

Show the field ONLY if: [icu_types(2)] = '1'

[ picu_addl_beds ]

Show the field ONLY if: [picu_adq_beds] = '0'

[ picu_addl_y_not ]

If yes, how many additional pediatric patients could be admitted?

Are there isolation rooms in the adult ICU that can be used for pediatric patients?

If yes, number of dedicated isolation rooms

If yes, total number of beds in ICU isolation rooms

In an infectious disease emergency, do you have the capacity to cohort pediatric patients in the adult ICU?

If yes, how many pediatric patients?

If no, can you cohort pediatric patients requiring intensive care elsewhere?

If yes, where?

If yes, how many patients?

Section Header: *If you have a separate Pediatric Intensive Care Unit (PICU):*

How many beds are in the PICU?

What is the average daily census in the PICU?

What is the average bed occupancy rate in the PICU?

*as a percent*

Do you have an adequate number of beds in the PICU to meet current needs?

If no, how many additional pediatric ICU beds are needed?

What would prevent you from adding the additional beds?

text

yesno

1. Yes
2. No text

text

yesno

1. Yes
2. No text

yesno

1. Yes
2. No text

text

text

text

text (integer, Min: 1, Max: 100)

yesno

1. Yes
2. No text

checkbox

165

Show the field ONLY if: [picu_adq_beds] = '0'

[ picu_addl_y_not_other ]

Show the field ONLY if: [picu_addl_y_not(5)] = '1'

*please identify the MAIN reason(s)*

If you selected other, please specify

1

2

3

4

5

text

picu_addl_y_not 1

picu_addl_y_not 2

picu_addl_y_not 3

picu_addl_y_not 4

picu_addl_y_not 5

Staffing (number of nurses) Staffing (number of doctors) Finances

Space Other

| 166 | [ picu_services ]  Show the field ONLY if: [icu_types(2)] = '1' | Which of the following are available for patients in PICU beds? | checkbox | | | |
| --- | --- | --- | --- | --- | --- | --- |
|  |  |  | 1 | picu_services 1 | | Pulse oximetry |
|  |  |  | 2 | picu_services 2 | | Continuous ECG |
|  |  |  | 3 | picu_services 3 | | Invasive pressure monitoring |
|  |  |  | 4 | picu_services 4 | | Mechanical ventilators |
|  |  |  | 5 | picu_services 5 | | CPAP |
|  |  |  | 6 | picu_services 6 | | Oxygen concentrators |
|  |  |  | 7 | picu_services 7 | | Oxygen cylinders |
|  |  |  | 8 | picu_services 8 | | Peritoneal dialysis |
|  |  |  | 9 | picu_services 9 | | Hemodialysis |
|  |  |  | 10 | picu_services 10 | | Cooling devices for induced hypothermia |
|  |  |  | 11 | picu_services 11 | | High frequency oscillators (HFVO) |
|  |  |  | 12 | picu_services 12 | | EEG monitors (Brainz monitors) |
|  |  |  | 13 | picu_services 13 | | Ultrasound imaging in the ICU |
|  |  |  | 14 | picu_services 14 | | Phototherapy |
|  |  |  | 15 | picu_services 15 | | Central lines |
| 167 | [ picu_ventilators ]  Show the field ONLY if: [picu_services(4)] = '1' | How many mechanical ventilators do you have in the PICU? | text (integer) | | | |
| 168 | [ picu_funct_vent ]  Show the field ONLY if: [picu_services(4)] = '1' | How many mechanical ventilator units are functional on a given day? | text (integer) | | | |
| 169 | [ picu_cpap ]  Show the field ONLY if: [picu_services(5)] = '1' | How many CPAP units do you have in the PICU? | text (integer) | | | |
| 170 | [ picu_funct_cpap ]  Show the field ONLY if: [picu_services(5)] = '1' | How many CPAP units are functional on a given day? | text (integer) | | | |
| 171 | [ picu_o2_conc ]  Show the field ONLY if: [picu_services(6)] = '1' | How many oxygen concentrators do you have in the PICU? | text (integer) | | | |
| 172 | [ picu_funct_o2_conc ]  Show the field ONLY if: [picu_services(6)] = '1' | How many oxygen concentrator units are functional on a given day? | text (integer) | | | |
| 173 | [ picu_o2_cylinders ]  Show the field ONLY if: [picu_services(7)] = '1' | How many oxygen cylinders do you have in the PICU? | text (integer) | | | |
| 174 | [ picu_surge ]  Show the field ONLY if: [icu_types(2)] = '1' | Do you have surge capacity in the PICU? | yesno | |  | |
|  |  |  | 1 | Yes |  |  |
|  |  |  | 0 | No |  |  |
| 175 | [ picu_surge_yes ]  Show the field ONLY if: [picu_surge] = '1' | If yes, how many additional patients could be admitted to the PICU? | text | | | |
| 176 | [ picu_surge_no ]  Show the field ONLY if: [picu_surge] = '0' | If no, is there PICU surge capacity elsewhere? | yesno | |  | |
|  |  |  | 1 | Yes |  |  |
|  |  |  | 0 | No |  |  |
| 177 | [ picu_surge_where ]  Show the field ONLY if: [picu_surge_no] = '1' | If yes, where? | text | | | |

178

179

180

181

182

183

184

185

186

187

188

189

190

191

192

[ picu_surge_other_n ]

Show the field ONLY if: [picu_surge_no] = '1'

[ picu_isolation ]

Show the field ONLY if: [icu_types(2)] = '1'

[ picu_n_isolation ]

Show the field ONLY if: [picu_isolation] = '1'

[ picu_n_beds_iso ]

Show the field ONLY if: [picu_isolation] = '1'

[ picu_cohort ]

Show the field ONLY if: [icu_types(2)] = '1'

[ picu_cohort_n ]

Show the field ONLY if: [picu_cohort] = '1'

[ picu_cohort_no ]

Show the field ONLY if: [picu_cohort] = '0'

[ picu_cohort_where ]

Show the field ONLY if: [picu_cohort_no] = '1'

[ picu_cohort_no_n ]

Show the field ONLY if: [picu_cohort_no] = '1'

[ nicu_n_beds ]

Show the field ONLY if: [icu_types(3)] = '1'

[ nicu_adc ]

Show the field ONLY if: [icu_types(3)] = '1'

[ nicu_bor ]

Show the field ONLY if: [icu_types(3)] = '1'

[ nicu_adq_beds ]

Show the field ONLY if: [icu_types(3)] = '1'

[ nicu_addl_beds ]

Show the field ONLY if: [nicu_adq_beds] = '0'

[ nicu_addl_y_not ]

If yes, how many additional PICU patients could be admitted?

Are there isolation rooms in the PICU?

If yes, number of dedicated isolation rooms in the PICU

If yes, total number of beds in PICU isolation rooms

In an infectious disease emergency, do you have the capacity to cohort patients PICU?

If yes, how many patients?

If no, can you cohort pediatric patients requiring intensive care elsewhere?

If yes, where?

If yes, how many patients?

Section Header: *If you have a separate Neonatal Intensive Care Unit (NICU):*

How many beds are in the NICU?

What is the average daily census in the NICU?

What is the average bed occupancy rate in the NICU?

*as a percent*

Do you have an adequate number of beds in the NICU to meet current needs?

If no, how many additional NICU beds are needed?

What would prevent you from adding the additional beds?

text

yesno

1. Yes
2. No text

text

yesno

1. Yes
2. No text

yesno

1. Yes
2. No text

text

text

text

text (integer, Min: 1, Max: 100)

yesno

1. Yes
2. No text

checkbox

193

Show the field ONLY if: [nicu_adq_beds] = '0'

[ nicu_addl_y_not_other ]

Show the field ONLY if: [nicu_addl_y_not(5)] = '1'

*please identify the MAIN reason(s)*

If you selected other, please specify

1

2

3

4

5

text

nicu_addl_y_not 1

nicu_addl_y_not 2

nicu_addl_y_not 3

nicu_addl_y_not 4

nicu_addl_y_not 5

Staffing (number of nurses) Staffing (number of doctors) Finances

Space Other

| 194 | [ nicu_services ]  Show the field ONLY if: [icu_types(3)] = '1' | Which of the following are available for patients in NICU beds? | checkbox | | | |
| --- | --- | --- | --- | --- | --- | --- |
|  |  |  | 1 | nicu_services 1 | | Pulse oximetry |
|  |  |  | 2 | nicu_services 2 | | Continuous ECG |
|  |  |  | 3 | nicu_services 3 | | Invasive pressure monitoring |
|  |  |  | 4 | nicu_services 4 | | Mechanical ventilators |
|  |  |  | 5 | nicu_services 5 | | CPAP |
|  |  |  | 6 | nicu_services 6 | | Oxygen concentrators |
|  |  |  | 7 | nicu_services 7 | | Oxygen cylinders |
|  |  |  | 8 | nicu_services 8 | | Peritoneal dialysis |
|  |  |  | 9 | nicu_services 9 | | Hemodialysis |
|  |  |  | 10 | nicu_services 10 | | Cooling devices for induced hypothermia |
|  |  |  | 11 | nicu_services 11 | | High frequency oscillators (HFVO) |
|  |  |  | 12 | nicu_services 12 | | EEG monitors (Brainz monitors) |
|  |  |  | 13 | nicu_services 13 | | Ultrasound imaging in ICU |
|  |  |  | 14 | nicu_services 14 | | Phototherapy |
|  |  |  | 15 | nicu_services 15 | | Central lines |
| 195 | [ nicu_ventilators ]  Show the field ONLY if: [nicu_services(4)] = '1' | How many mechanical ventilators do you have in the NICU? | text (integer) | | | |
| 196 | [ nicu_funct_ventilators ]  Show the field ONLY if: [nicu_services(4)] = '1' | How many mechanical ventilator units are functional on a given day? | text (integer) | | | |
| 197 | [ nicu_cpap ]  Show the field ONLY if: [nicu_services(5)] = '1' | How many CPAP units do you have in the NICU? | text (integer) | | | |
| 198 | [ nicu_funct_cpap ]  Show the field ONLY if: [nicu_services(5)] = '1' | How many CPAP units are functional on a given day? | text (integer) | | | |
| 199 | [ nicu_o2_conc ]  Show the field ONLY if: [nicu_services(6)] = '1' | How many oxygen concentrators do you have in the NICU? | text (integer) | | | |
| 200 | [ nicu_funct_o2_conc ]  Show the field ONLY if: [nicu_services(6)] = '1' | How many oxygen concentrator units are functional on a given day? | text (integer) | | | |
| 201 | [ nicu_o2_cylinders ]  Show the field ONLY if: [nicu_services(7)] = '1' | How many oxygen cylinders do you have in the NICU? | text (integer) | | | |
| 202 | [ nicu_surge ]  Show the field ONLY if: [icu_types(3)] = '1' | Do you have surge capacity in the NICU? | yesno | |  | |
|  |  |  | 1 | Yes |  |  |
|  |  |  | 0 | No |  |  |
| 203 | [ nicu_surge_yes ]  Show the field ONLY if: [nicu_surge] = '1' | If yes, how many additional patients could be admitted to the NICU? | text | | | |
| 204 | [ nicu_surge_no ]  Show the field ONLY if: [nicu_surge] = '0' | If no, is there NICU surge capacity elsewhere? | yesno | |  | |
|  |  |  | 1 | Yes |  |  |
|  |  |  | 0 | No |  |  |
| 205 | [ nicu_surge_where ]  Show the field ONLY if: [nicu_surge_no] = '1' | If yes, where? | text | | | |

206

207

208

209

210

211

212

213

214

215

216

217

218

219

220

[ nicu_surge_other_n ]

Show the field ONLY if: [nicu_surge_no] = '1'

[ nicu_isolation ]

Show the field ONLY if: [icu_types(3)] = '1'

[ nicu_n_isolation ]

Show the field ONLY if: [nicu_isolation] = '1'

[ nicu_n_beds_iso ]

Show the field ONLY if: [nicu_isolation] = '1'

[ nicu_cohort ]

Show the field ONLY if: [icu_types(3)] = '1'

[ nicu_cohort_n ]

Show the field ONLY if: [nicu_cohort] = '1'

[ nicu_cohort_no ]

Show the field ONLY if: [nicu_cohort] = '0'

[ nicu_cohort_where ]

Show the field ONLY if: [nicu_cohort_no] = '1'

[ nicu_cohort_no_n ]

Show the field ONLY if: [nicu_cohort_no] = '1'

[ npicu_n_beds ]

Show the field ONLY if: [icu_types(4)] = '1'

[ npicu_adc ]

Show the field ONLY if: [icu_types(4)] = '1'

[ npicu_bor ]

Show the field ONLY if: [icu_types(4)] = '1'

[ npicu_adq_beds ]

Show the field ONLY if: [icu_types(4)] = '1'

[ npicu_beds_addl ]

Show the field ONLY if: [npicu_adq_beds] = '0'

[ npicu_beds_addl_size ]

Show the field ONLY if: [npicu_adq_beds] = '0'

If yes, how many additional NICU patients could be admitted?

Are there isolation rooms in the NICU?

If yes, number of dedicated isolation rooms in the NICU

If yes, total number of beds in NICU isolation rooms

In an infectious disease emergency, do you have the capacity to cohort patients the NICU?

If yes, how many patients?

If no, can you cohort neonatal patients requiring intensive care elsewhere?

If yes, where?

If yes, how many patients?

Section Header: *If you have a combined Neonatal/Pediatric Intensive Care Unit (NICU/PICU)*

How many beds are in the NICU/PICU?

What is the average daily census in the NICU/PICU?

What is the average bed occupancy rate in the NICU/PICU?

*as a percent*

Do you have an adequate number of beds in the NICU/PICU to meet current needs?

If no, how many additional NICU/PICU beds are needed?

If no, are more beds needed specifically for bigger or smaller children?

text

yesno

1. Yes
2. No text

text

yesno

1. Yes
2. No text

yesno

1. Yes
2. No text

text

text

text

text (integer, Min: 1, Max: 100)

yesno

1. Yes
2. No text

radio

1. bigger
2. smaller
3. both

| 221 | [ npicu_addl_y_not ]  Show the field ONLY if: [npicu_adq_beds] = '0' | What would prevent you from adding the additional beds?  *please identify the MAIN reason(s)* | checkbox | | | |
| --- | --- | --- | --- | --- | --- | --- |
|  |  |  | 1 | npicu_addl_y_not 1 | | Staffing (number of nurses) |
|  |  |  | 2 | npicu_addl_y_not 2 | | Staffing (number of doctors) |
|  |  |  | 3 | npicu_addl_y_not 3 | | Finances |
|  |  |  | 4 | npicu_addl_y_not 4 | | Space |
|  |  |  | 5 | npicu_addl_y_not 5 | | Other |
| 222 | [ npicu_addl_y_not_other ]  Show the field ONLY if: [npicu_addl_y_not(5)] = '1' | If you selected other, please specify | text | | | |
| 223 | [ npicu_reserved_beds ]  Show the field ONLY if: [icu_types(4)] = '1' | Are certain beds in the NICU/PICU reserved for neonatal patients? | yesno | |  | |
|  |  |  | 1 | Yes |  |  |
|  |  |  | 0 | No |  |  |
| 224 | [ npicu_n_reserved_beds ]  Show the field ONLY if: [npicu_reserved_beds] = '1' | If yes, how many beds are reserved for neonatal patients? | text | | | |
| 225 | [ npicu_reserved_beds_adq ]  Show the field ONLY if: [npicu_reserved_beds] = '1' | If yes, is the number of beds reserved for neonatal patients adequate for current needs? | yesno | |  | |
|  |  |  | 1 | Yes |  |  |
|  |  |  | 0 | No |  |  |
| 226 | [ npicu_reserved_beds_addl ]  Show the field ONLY if: [npicu_reserved_beds_adq] = '0' | If no, how many additional neonatal ICU beds are needed? | text | | | |
| 227 | [ npicu_services ]  Show the field ONLY if: [icu_types(4)] = '1' | Which of the following are available for patients in NICU/PICU beds? | checkbox | | | |
|  |  |  | 1 | npicu_services 1 | | Pulse oximetry |
|  |  |  | 2 | npicu_services 2 | | Continuous ECG |
|  |  |  | 3 | npicu_services 3 | | Invasive pressure monitoring |
|  |  |  | 4 | npicu_services 4 | | Mechanical ventilators |
|  |  |  | 5 | npicu_services 5 | | CPAP |
|  |  |  | 6 | npicu_services 6 | | Oxygen concentrators |
|  |  |  | 7 | npicu_services 7 | | Oxygen cylinders |
|  |  |  | 8 | npicu_services 8 | | Peritoneal dialysis |
|  |  |  | 9 | npicu_services 9 | | Hemodialysis |
|  |  |  | 10 | npicu_services 10 | | Cooling devices for induced hypothermia |
|  |  |  | 11 | npicu_services 11 | | High frequency oscillators (HFVO) |
|  |  |  | 12 | npicu_services 12 | | EEG monitors (Brainz monitors) |
|  |  |  | 13 | npicu_services 13 | | Ultrasound imaging in the ICU |
|  |  |  | 14 | npicu_services 14 | | Phototherapy |
|  |  |  | 15 | npicu_services 15 | | Central lines |
| 228 | [ npicu_ventilators ]  Show the field ONLY if: [npicu_services(4)] = '1' | How many mechanical ventilators do you have in the NICU? | text (integer) | | | |
| 229 | [ npicu_funct_vent ]  Show the field ONLY if: [npicu_services(4)] = '1' | How many mechanical ventilator units are functional on a given day? | text (integer) | | | |
| 230 | [ npicu_cpap ]  Show the field ONLY if: [npicu_services(5)] = '1' | How many CPAP units do you have in the NICU/PICU? | text (integer) | | | |

| 231 | [ npicu_funct_cpap ]  Show the field ONLY if: [npicu_services(5)] = '1' | How many CPAP units are functional on a given day? | text (integer) | | |
| --- | --- | --- | --- | --- | --- |
| 232 | [ npicu_o2_conc ]  Show the field ONLY if: [npicu_services(6)] = '1' | How many oxygen concentrators do you have in the NICU/PICU? | text (integer) | | |
| 233 | [ npicu_funct_o2_conc ]  Show the field ONLY if: [npicu_services(6)] = '1' | How many oxygen concentrator units are functional on a given day? | text (integer) | | |
| 234 | [ npicu_o2_cylinders ]  Show the field ONLY if: [npicu_services(7)] = '1' | How many oxygen cylinders do you have in the NICU/PICU? | text (integer) | | |
| 235 | [ npicu_surge ]  Show the field ONLY if: [icu_types(4)] = '1' | Do you have surge capacity in the NICU/PICU? | yesno | |  |
|  |  |  | 1 | Yes |  |
|  |  |  | 0 | No |  |
| 236 | [ npicu_surge_yes ]  Show the field ONLY if: [npicu_surge] = '1' | If yes, how many additional patients could be admitted to the NICU/PICU? | text | | |
| 237 | [ npicu_surge_no ]  Show the field ONLY if: [npicu_surge] = '0' | If no, is there NICU/PICU surge capacity elsewhere? | yesno | |  |
|  |  |  | 1 | Yes |  |
|  |  |  | 0 | No |  |
| 238 | [ npicu_surge_where ]  Show the field ONLY if: [npicu_surge_no] = '1' | If yes, where? | text | | |
| 239 | [ npicu_surge_other_n ]  Show the field ONLY if: [npicu_surge_no] = '1' | If yes, how many additional patients could be admitted? | text | | |
| 240 | [ npicu_isolation ]  Show the field ONLY if: [icu_types(4)] = '1' | Are there isolation rooms in the NICU/PICU? | yesno | |  |
|  |  |  | 1 | Yes |  |
|  |  |  | 0 | No |  |
| 241 | [ npicu_n_isolation ]  Show the field ONLY if: [npicu_isolation] = '1' | If yes, number of dedicated isolation rooms in the NICU/PICU | text | | |
| 242 | [ npicu_n_beds_iso ]  Show the field ONLY if: [npicu_isolation] = '1' | If yes, total number of beds in NICU/PICU isolation rooms | text | | |
| 243 | [ npicu_cohort ]  Show the field ONLY if: [icu_types(4)] = '1' | In an infectious disease emergency, do you have the capacity to cohort patients NICU/PICU? | yesno | |  |
|  |  |  | 1 | Yes |  |
|  |  |  | 0 | No |  |
| 244 | [ npicu_cohort_n ]  Show the field ONLY if: [npicu_cohort] = '1' | If yes, how many patients? | text | | |
| 245 | [ npicu_cohort_no ]  Show the field ONLY if: [npicu_cohort] = '0' | If no, can you cohort pediatric patients requiring intensive care elsewhere? | yesno | |  |
|  |  |  | 1 | Yes |  |
|  |  |  | 0 | No |  |
| 246 | [ npicu_cohort_where ]  Show the field ONLY if: [npicu_cohort_no] = '1' | If yes, where? | text | | |
| 247 | [ npicu_cohort_no_n ]  Show the field ONLY if: [npicu_cohort_no] = '1' | If yes, how many patients? | text | | |
| 248 | [ icu_clarify ] | Section Header: *ICU clariications*  This is a space to clarify any answers from the ICU section | notes | | |

249

250

251

252

253

254

255

256

257

258

259

260

261

262

263

264

[ opd_background ]

[ opd_use ]

[ opd_sep ]

[ opd_sep_no ]

Show the field ONLY if: [opd_sep] = '0'

[ opd_max_pat ]

[ opd_max_pat_calc ]

[ opd_n_seen_per_day ]

[ opd_highest_seen ]

[ opd_high_reason ] Show the field ONLY if:

[opd_highest_seen] = 'unknow n' OR [opd_highest_seen] = 'U nknown' OR [opd_highest_see n] = '"unknown"'

[ opd_surge ]

[ opd_surge_n ]

Show the field ONLY if: [opd_surge] = '1'

[ opd_surge_no ]

Show the field ONLY if: [opd_surge] = '0'

[ opd_surge_where ]

Show the field ONLY if: [opd_surge_no] = '1'

[ opd_surge_no_n ]

Show the field ONLY if: [opd_surge_no] = '1'

[ opd_triage ]

[ opd_wait_time ]

Section Header: *Outpatient*

This is a space to give background on the outpatient area set up in your hospital.

What is the outpatient area used for? (i.e., outpatient subspecialty clinic visits, general pediatric care, etc).

Do you have a separate pediatric outpatient area?

If no, where are pediatric outpatients seen?

What is the maximum number of pediatric patients that can be seen at one time in the outpatient area?

Please specify how the number above is calculated (i.e. based on total number of examining rooms, observation beds, etc.)?

What is the average number of pediatric outpatients seen on a given day?

What was the highest number of pediatric outpatients seen on one day in the past year?

*If the number is unknown please write "unknown"*

If the information is available, what was the reason for the high census?

Do you have surge capacity for pediatric patients in the outpatient area?

If yes, how many additional patients (beyond your usual maximum capacity) could be seen in the outpatient area?

If no, is there pediatric outpatient surge capacity elsewhere?

If yes, where?

If yes, how many additional patients could be seen?

Is there a triage system for pediatric outpatients as they enter the outpatient area?

If yes, please explain

On average, how long (in hours) must a patient wait to be seen in

notes

notes

yesno

1 Yes

1. No text

text

notes

text

text

text

yesno

1. Yes
2. No text

yesno

1. Yes
2. No text

text

notes

radio

the outpatient department?

1. < 1 hour
2. 1-3 hours
3. 4-8 hours
4. 8-24 hours
5. > 24 hours

265

266

[ outpatient_clarify ] [ er_background ]

This is a space to clarify any answers from the outpatient section.

Section Header: *Emergency (Casualty) Area*

This is a space to give background on the emergency (casualty) area set up in your hospital

notes notes

267

268

269

270

271

272

273

274

275

276

277

278

279

280

281

[ er_sep ]

[ er_n_beds ]

Show the field ONLY if: [er_sep] = '1'

[ er_no_sep ]

Show the field ONLY if: [er_sep] = '0'

[ er_no_sep_n_beds ]

Show the field ONLY if: [er_sep] = '0'

[ er_avg_seen ]

[ er_high_census ]

[ er_high_census_reason ]

[ er_surge ]

[ er_surge_n ]

Show the field ONLY if: [er_surge] = '1'

[ er_surge_other ]

Show the field ONLY if: [er_surge] = '0'

[ er_surge_other_where ]

Show the field ONLY if: [er_surge_other] = '1'

[ er_surge_other_n ]

Show the field ONLY if: [er_surge_other] = '1'

[ er_infectious_triage ]

[ er_infectious_explain ]

Show the field ONLY if: [er_infectious_triage] = '1'

[ er_wait_time ]

Do you have a separate pediatric emergency (casualty) area?

If yes, how many beds are in the pediatric emergency (casualty) area?

If no, where are pediatric patient seen for emergency services?

How many beds are available for pediatric patients in this area?

What is the average number of pediatric patients seen in the emergency (casualty) area on a given day?

What was the highest daily census in the emergency (casualty) area in the past year?

*If the number is unknown please write "unknown"*

If the information is available, what was the reason for the high census?

Do you have surge capacity for pediatric patients in the emergency (casualty) area?

If yes, how many additional pediatric patients (beyond your usual maximum capacity) could be seen?

If no, is there pediatric emergency area surge capacity elsewhere?

If yes, where?

If yes, how many additional pediatric patients could be seen?

Is there a mechanism to quickly identify and isolate patients with contagious diseases as they enter the emergency (casualty) area?

If yes, please explain.

On average, how long must a patient wait to be seen in the

yesno

1 Yes

1. No text

text

text

text

text

text

yesno

1. Yes
2. No text

yesno

1. Yes
2. No text

text

yesno

1. Yes
2. No notes

radio

emergency (casualty) area?

1. < 1 hour
2. 1-3 hours
3. 4-8 hours
4. 8-24 hours
5. > 24 hours

282

283

[ er_clarify ]

[ departments_complete ]

This is a space to clarify any answers from the emergency (casualty) area section

Section Header: *Form Status*

notes

dropdown

Complete?

Instrument: Capacity/Services (capacityservices)  Enabled as survey

1. Incomplete
2. Unverified
3. Complete

| 284 | [ services ] | For the questions in this section, please answer for your facility OR your network where applicable. | descriptive | | | |
| --- | --- | --- | --- | --- | --- | --- |
| 285 | [ surgery_background ] | Section Header: *Surgery*  This is a space to give background on surgical services in your hospital or network.  *e.g. indicate if all surgeries or speciic surgeries are conducted at another hospital in your network* | notes | | | |
| 286 | [ surgery_where ] | Is surgery for pediatric patients:  *check all that apply* | checkbox | | | |
|  |  |  | 1 | surgery_where 1 | | performed in your hospital |
|  |  |  | 2 | surgery_where 2 | | up-referred to another hospital in your network of hospitals |
|  |  |  | 3 | surgery_where 3 | | up-referred to a hospital outside of your network of hospitals |
| 287 | [ surgery_where_referred ]  Show the field ONLY if: [surgery_where(2)] = '1' or [sur gery_where(3)] = '1' | If surgeries are up-referred, where, and for what kinds of surgeries? | notes | | | |
| 288 | [ n_ped_ors ]  Show the field ONLY if: [surgery_where(1)] = '1' or [sur gery_where(2)] = '1' | Number of DEDICATED pediatric operating theatres in your hospital/the hospital in your network where pediatric surgeries are performed  *If there are no dedicated pediatric theatres, enter "0"* | text (integer) | | | |
| 289 | [ n_ped_ors_available ]  Show the field ONLY if: [n_ped_ors] > 0 | How many dedicated pediatric operating theatres are available on a daily basis? | text | | | |
| 290 | [ n_gen_ors ]  Show the field ONLY if: [surgery_where(1)] = '1' or [sur gery_where(2)] = '1' | How many general (adult/pediatric) operating theatres do you have that can be used for pediatric surgery?  *If there are no general or adult theatres that are used for children, enter "0"* | text | | | |
| 291 | [ n_gen_ors_for_ped ]  Show the field ONLY if: [n_gen_ors] > 0 | How many of the general operating theatres are used for pediatric surgery on a daily basis? | text (integer) | | | |
| 292 | [ peak_ors_used_per_day ]  Show the field ONLY if: [surgery_where(1)] = '1' or [sur gery_where(2)] = '1' | What is the peak number of operating theatres used for all pediatric surgery per day? | text (integer) | | | |
| 293 | [ adequate_ors ]  Show the field ONLY if: [surgery_where(1)] = '1' or [sur gery_where(2)] = '1' | Do you have an adequate number of operating theatres to meet your current needs for pediatric surgery (assuming that you have access to a full complement of nurses, surgeons, anesthesiologists, and other staff)? | yesno  1 Yes  0 No | | | |
| 294 | [ addl_ors_needed ]  Show the field ONLY if: [adequate_ors] = '0' | If no, how many additional operating theatres are needed? | text (integer) | | | |
| 295 | [ avg_n_surguries ]  Show the field ONLY if: [surgery_where(1)] = '1' or [sur gery_where(2)] = '1' | Average number of pediatric surgeries performed per week | text (integer) | | | |
| 296 | [ surgery_list ]  Show the field ONLY if: [surgery_where(1)] = '1' or [sur gery_where(2)] = '1' | Please attach a list of pediatric surgical cases over the past month (please indicate age of patient and type of case: e.g. 2-year-old appendectomy) | file | | | |
| 297 | [ surgery_wait ]  Show the field ONLY if: [surgery_where(1)] = '1' or [sur gery_where(2)] = '1' | Approximately how long does a child have to wait for an elective surgery (e.g. club foot or cleft lip)? | radio | | | |
|  |  |  | 1 | < 1 week |  | |
|  |  |  | 2 | 1 week - 1 month |  |  |
|  |  |  | 3 | 1-3 months |  |  |
|  |  |  | 4 | 3-6 months |  |  |
|  |  |  | 5 | > 6 months |  |  |

| 1 | anesthetic_agents 1 | barbiturates |
| --- | --- | --- |
| 2 | anesthetic_agents 2 | propofol |
| 3 | anesthetic_agents 3 | ketamine |
| 4 | anesthetic_agents 4 | dexmetatomidine |
| 5 | anesthetic_agents 5 | benzodiazepines |
| 6 | anesthetic_agents 6 | narcotics |
| 7 | anesthetic_agents 7 | succinylcholine |
| 8 | anesthetic_agents 8 | pancuronium |
| 9 | anesthetic_agents 9 | vecuronium |
| 10 | anesthetic_agents 10 | rocuronium |
| 11 | anesthetic_agents 11 | cisatracurium |
| 12 | anesthetic_agents 12 | atracurium |
| 13 | anesthetic_agents 13 | mivacurium |
| 14 | anesthetic_agents 14 | sevoflurane |
| 15 | anesthetic_agents 15 | halothane |
| 16 | anesthetic_agents 16 | desflurane |
| 17 | anesthetic_agents 17 | isoflurane |
| 18 | anesthetic_agents 18 | methoxyflurane |
| 19 | anesthetic_agents 19 | ether |
| 20 | anesthetic_agents 20 | chloroform |
| 21 | anesthetic_agents 21 | naloxone |
| 22 | anesthetic_agents 22 | sugammadex |
| 23 | anesthetic_agents 23 | flumazenil |

| 298 | [ sterilize_tools ]  Show the field ONLY if: [surgery_where(1)] = '1' or [sur gery_where(2)] = '1' | How are reusable tools/equipment sterilized?  *check all that apply* | checkbox | | | | |
| --- | --- | --- | --- | --- | --- | --- | --- |
|  |  |  | 1 | sterilize_tools 1 | Autoclave | |  |
|  |  |  | 2 | sterilize_tools 2 | Dry heat | |  |
|  |  |  | 3 | sterilize_tools 3 | Radiation | |  |
|  |  |  | 4 | sterilize_tools 4 | Chemically | |  |
|  |  |  | 5 | sterilize_tools 5 | Other | |  |
| 299 | [ sterilize_tools_other ]  Show the field ONLY if: [sterilize_tools(5)] = '1' | Please specify other method(s) by which reusable tools are sterilized. | text | | | | |
| 300 | [ surgery_clarify ] | This is a space to clarify any answers from the surgery section. | notes | | | | |
| 301 | [ anesthesia_methods ]  Show the field ONLY if: [surgery_where(1)] = '1' or [sur gery_where(2)] = '1' | Section Header: *Anesthesia*  Which of the following methods are available for the administration of anesthetic agents | checkbox | | | | |
|  |  |  | 1 | anesthesia_methods 1 | | Vaporizer | |
|  |  |  | 2 | anesthesia_methods 2 | | Copper kettle | |
|  |  |  | 3 | anesthesia_methods 3 | | Open drop | |
|  |  |  | 4 | anesthesia_methods 4 | | By intravenous therapy (IV) | |
|  |  |  | 5 | anesthesia_methods 5 | | By ether and chloroform drip | |
|  |  |  | 6 | anesthesia_methods 6 | | Other | |
| 302 | [ other_anesthesia_methods ]  Show the field ONLY if: [anesthesia_methods(6)] = '1' | Please specify which other methods for the administration of anesthetic agents are available. | text | | | | |
| 303 | [ anesthetic_agents ]  Show the field ONLY if: [surgery_where(1)] = '1' or [sur gery_where(2)] = '1' | What anesthetic agents are available?  *check all that apply* | checkbox  Custom alignment: RH | | | | |
| 304 | [ anesthesia_clarify ] | This is a space to clarify any answers from the anesthesia section. | notes | | | | |

305

306

[ pharmacy_where ]

[ pharmacy_where_outside ]

Show the field ONLY if: [pharmacy_where(1)] = '0'

Section Header: *Pharmacy*

Do you rely on pharmacy services available:

*check all that apply*

If not in your hospital, where are pharmacy services available?

checkbox

1. pharmacy_where 1 in your hospital
2. pharmacy_where 2 in your network of

hospitals

text

307

308

309

310

311

[ national_formulary ]

[ national_formulary_attach ]

Show the field ONLY if: [national_formulary] = '1'

[ national_formulary_url ]

Show the field ONLY if: [national_formulary] = '1'

[ community_pharm ]

[ community_pharm_freq ]

Do you have a national formulary (essential medicines list)?

Please attach a copy of the national formulary here or insert URL below

Paste URL here:

Does your hospital ever send parents of pediatric inpatients out to commercial pharmacies in the community to purchase medications that are not available at the hospital

If yes, how frequently?

yesno

1. Yes
2. No file

text

yesno

1. Yes
2. No

radio

Show the field ONLY if: [community_pharm] = '1'

1. always
2. most times
3. occasionally
4. rarely
5. never

312

313

314

315

316

317

318

[ commun_pharm_circum ]

Show the field ONLY if: [community_pharm] = '1'

[ dispensing_pharm_in ]

[ dispensing_pharm_in_hrs ]

Show the field ONLY if: [dispensing_pharm_in] = '1'

[ dispensing_pharm_out ]

[ dispensing_pharm_out_hrs ]

Show the field ONLY if: [dispensing_pharm_out] = '1'

[ compounding_pharm ]

[ med_payment ]

If yes, in what circumstances are parents sent to commercial pharmacies in the community?

In your hospital/network, do you have a dispensing pharmacy for inpatients?

What are the inpatient pharmacy hours of operation?

*Days, Hours*

In your hospital/network, do you have a dispensing pharmacy for outpatients?

What are the outpatient pharmacy hours of operation?

*Days, Hours*

Do you have a compounding pharmacy (i.e. to prepare medications not commercially available such as liquid ciprofloxacin suspension or "magic mix")?

Who pays for medications?

notes

yesno

1 Yes

1. No text

yesno

1. Yes
2. No text

yesno

1. Yes
2. No

checkbox

*check all that apply*

1. med_payment 1
2. med_payment 2
3. med_payment 3
4. med_payment 4

patient government private insurance national insurance

319

[ pay_for_meds ]

Are there any circumstances where the child/parent has to pay for essential medications?

yesno

1 Yes

1. No

| 320 | [ pay_for_meds_yes ]  Show the field ONLY if: [pay_for_meds] = '1' | If yes, please specify. | notes | | | | | |
| --- | --- | --- | --- | --- | --- | --- | --- | --- |
| 321 | [ pharmacy_clarify ] | This is a space to clarify any answers from the pharmacy section. | notes | | | | | |
| 322 | [ radiology_where ] | Section Header: *Radiology*  Are radiological services:  *check all that apply* | checkbox | | | | | |
|  |  |  | 1 | radiology_where 1 | | | provided in your hospital | |
|  |  |  | 2 | radiology_where 2 | | | up-referred to another hospital in your network of hospitals | |
|  |  |  | 3 | radiology_where 3 | | | up-referred to a hospital outside your network | |
| 323 | [ radiology_equipment ] | Which of the following services are available at your hospital? | checkbox | | | | | |
|  |  |  | 1 | radiology_equipment 1 | | | | Ultrasound |
|  |  |  | 2 | radiology_equipment 2 | | | | X-ray |
|  |  |  | 3 | radiology_equipment 3 | | | | Computed Tomography scan (CT) |
|  |  |  | 4 | radiology_equipment 4 | | | | Magnetic Resonance Imaging scan (MRI) |
|  |  |  | 5 | radiology_equipment 5 | | | | Positron Emission Tomography (PET) scan |
|  |  |  | 6 | radiology_equipment 6 | | | | Diagnostic nuclear medicine capabilities (e.g. bone, renal, or tagged white cell scan) |
|  |  |  | 7 | radiology_equipment 7 | | | | Therapeutic Nuclear Medicine capabilities (e.g. radiotherapy for malignancies) |
| 324 | [ radiology_refer_intro ] | If not available in your facility, do you refer patients to receive the following services: | descriptive | | | | | |
| 325 | [ ultrasound_refer ]  Show the field ONLY if: [radiology_equipment(1)] = '0' | Ultrasound | checkbox | | | | | |
|  |  |  | 1 | ultrasound_refer 1 | | | to another hospital in your network of hospitals | |
|  |  |  | 2 | ultrasound_refer 2 | | | to a hospital outside your network of hospitals | |
| 326 | [ xray_refer ]  Show the field ONLY if: [radiology_equipment(2)] = '0' | X-ray/radiogram | checkbox | | | | | |
|  |  |  | 1 | xray_refer 1 | | to another hospital in your network of hospitals | | |
|  |  |  | 2 | xray_refer 2 | | to a hospital outside your network of hospitals | | |
| 327 | [ ct_refer ]  Show the field ONLY if: [radiology_equipment(3)] = '0' | Computed Tomography scan (CT) | checkbox | | | | | |
|  |  |  | 1 | ct_refer 1 | to another hospital in your network of hospitals | | | |
|  |  |  | 2 | ct_refer 2 | to a hospital outside your network of hospitals | | | |
| 328 | [ mri_refer ]  Show the field ONLY if: [radiology_equipment(4)] = '0' | Magnetic Resonance Imaging scan (MRI) | checkbox | | | | | |
|  |  |  | 1 | mri_refer 1 | | to another hospital in your network of hospitals | | |
|  |  |  | 2 | mri_refer 2 | | to a hospital outside your network of hospitals | | |
| 329 | [ pet_refer ]  Show the field ONLY if: [radiology_equipment(5)] = '0' | Positron Emission Tomography (PET) scan | checkbox | | | | | |
|  |  |  | 1 | pet_refer 1 | | to another hospital in your network of hospitals | | |
|  |  |  | 2 | pet_refer 2 | | to a hospital outside your network of hospitals | | |

330

[ dnm_refer ]

Show the field ONLY if: [radiology_equipment(6)] = '0'

Diagnostic nuclear medicine (e.g. bone, renal, or tagged white cell scan)

checkbox

1. dnm_refer 1 to another hospital in your

network of hospitals

1. dnm_refer 2

to a hospital outside your network of hospitals

331

[ tnm_refer ]

Therapeutic Nuclear Medicine (e.g. radiotherapy for malignancies)

checkbox

332

Show the field ONLY if: [radiology_equipment(7)] = '0'

[ radiotherapy_referral_site ]

Show the field ONLY if: [tnm_refer(1)] = '1' or [tnm_ref er(2)] = '1'

Where do you refer a patient that requires radiotherapy?

1

2

text

tnm_refer 1

tnm_refer 2

to another hospital in your network of hospitals

to a hospital outside your network of hospitals

333

334

335

336

337

338

339

340

341

342

343

344

345

[ linear_accelerator ]

Show the field ONLY if: [radiology_equipment(7)] = '1'

[ radionuclide_therapy ]

[ radioactive_isotopes ]

Show the field ONLY if: [radionuclide_therapy] = '1'

[ radioisotope_attach ]

Show the field ONLY if: [radionuclide_therapy] = '1'

[ radioisotope_attach_2 ]

Show the field ONLY if: [radioisotope_attach] <> ''

[ radioisotope_attach_3 ]

Show the field ONLY if: [radioisotope_attach_2] <> ''

[ radioisotope_url ]

Show the field ONLY if: [radionuclide_therapy] = '1'

[ radioisotope_url_2 ]

Show the field ONLY if: [radioisotope_url] <> ''

[ radioisotope_url_3 ]

Show the field ONLY if: [radioisotope_url_2] <> ''

[ blood_irradiator ]

[ cesium_137 ]

Show the field ONLY if: [blood_irradiator] = '1'

[ irradiator_security ]

Show the field ONLY if: [cesium_137] = '1'

[ cs137_attach ]

Show the field ONLY if: [cesium_137] = '1'

Do you have a linear accelerator on-site?

Is radiotherapy provided in your hospital/network?

What radioactive isotopes are in use (i.e. cobalt., etc.)?

If radioisotopes are in use, please attach the SOP or insert the URL below that outlines their safe use and disposal

Additional file

Additional file

Paste URL here:

Additional URL

Additional URL

Do you have a blood irradiator in your hospital/network?

Do you have a blood irradiator that uses Cesium-137?

What are the security practices around the Cs-137 blood irradiator?

Please attach security SOPs or guidelines for the blood irradiator here, or as a URL below.

yesno

1 Yes

1. No

yesno

1. Yes
2. No text

file

file

file

text

text

text

yesno

1. Yes
2. No

yesno

1. Yes
2. No notes

file

| 346 | [ cs137_attach_2 ]  Show the field ONLY if: [cs137_attach] <> '' | Additional file | file | | |
| --- | --- | --- | --- | --- | --- |
| 347 | [ cs137_attach_3 ]  Show the field ONLY if: [cs137_attach_2] <> '' | Additional file | file | | |
| 348 | [ cs137_url ]  Show the field ONLY if: [cesium_137] = '1' | Paste URL here: | text | | |
| 349 | [ cs137_url_2 ]  Show the field ONLY if: [cs137_url] <> '' | Additional URL | text | | |
| 350 | [ cs137_url_3 ]  Show the field ONLY if: [cs137_url_2] <> '' | Additional URL | text | | |
| 351 | [ radiology_clarify ] | This is a space to clarify any answers from the radiology section. | notes | | |
| 352 | [ autopsy_services ] | Section Header: *Autopsy*  Do you have autopsy services at your facility? | yesno | |  |
|  |  |  | 1 | Yes |  |
|  |  |  | 0 | No |  |
| 353 | [ n_autopsies ]  Show the field ONLY if: [autopsy_services] = '1' | If yes, how many autopsies did you perform on children last year? | text (integer) | | |
| 354 | [ refer_autopsy ]  Show the field ONLY if: [autopsy_services] = '0' | If no, do you up-refer for autopsy services? | yesno | |  |
|  |  |  | 1 | Yes |  |
|  |  |  | 0 | No |  |
| 355 | [ refer_autopsy_where ]  Show the field ONLY if: [autopsy_services] = '0' | If yes, where? | text | | |
| 356 | [ autopsy_clarify ] | This is a space to clarify any answers from the autopsy section.  *e.g. may indicate that police service will oversee autopsy in cases of suspicious deaths* | notes | | |
| 357 | [ dental_services ] | Section Header: *Dentistry*  Do you provide dental service for children in your hospital/network? | yesno | |  |
|  |  |  | 1 | Yes |  |
|  |  |  | 0 | No |  |
| 358 | [ dental_refer ]  Show the field ONLY if: [dental_services] = '0' | If no, do you refer elsewhere for dental services? | yesno | |  |
|  |  |  | 1 | Yes |  |
|  |  |  | 0 | No |  |
| 359 | [ dental_refer_where ]  Show the field ONLY if: [dental_refer] = '1' | If yes, where?  *(e.g. another hospital, general dentists practicing in the community)* | text | | |
| 360 | [ n_dental_chairs ]  Show the field ONLY if: [dental_services] = '1' | Number of dental chairs | text (integer) | | |
| 361 | [ adequate_dental_chairs ]  Show the field ONLY if: [dental_services] = '1' | Do you have an adequate number of chairs to meet current needs? | yesno | |  |
|  |  |  | 1 | Yes |  |
|  |  |  | 0 | No |  |
| 362 | [ addl_dental_chairs_needed ]  Show the field ONLY if: [adequate_dental_chairs] = '0' | If no, how many additional dental chairs are needed? | text (integer) | | |
| 363 | [ dentistry_clarify ] | This is a space to clarify any answers from the dentistry section. | notes | | |
| 364 | [ malnutrition_ward ] | Section Header: *Nutrition*  Do you have a malnutrition ward? | yesno | |  |
|  |  |  | 1 | Yes |  |
|  |  |  | 0 | No |  |

365

366

367

368

[ malnutrition_ward_beds ]

Show the field ONLY if: [malnutrition_ward] = '1'

[ adequate_malnutrition_beds ]

Show the field ONLY if: [malnutrition_ward] = '1'

[ addl_malnut_beds_needed ]

Show the field ONLY if: [adequate_malnutrition_beds]

= '0'

[ malnut_addl_y_not ]

If yes, how many beds are in the malnutrition ward?

Do you have an adequate number of beds in the malnutrition ward to meet current needs (assuming you have a full complement of medical staff)?

If no, how many additional beds are needed?

What would prevent you from adding the additional beds?

text (integer)

yesno

1. Yes
2. No

text (integer)

checkbox

369

Show the field ONLY if: [adequate_malnutrition_beds]

= '0'

[ malnut_addl_y_not_other ]

Show the field ONLY if: [malnut_addl_y_not(5)] = '1'

*please identify the MAIN reason(s)*

If you selected other, please specify

1

2

3

4

5

text

malnut_addl_y_not 1

malnut_addl_y_not 2

malnut_addl_y_not 3

malnut_addl_y_not 4

malnut_addl_y_not 5

Staffing (number of nurses)

Staffing (number of doctors)

Finances Space Other

370

371

372

373

374

375

376

377

378

[ parenteral_nutrition ]

[ parenteral_nutrition_other ]

[ parenteral_nutrition_types ]

Show the field ONLY if: [parenteral_nutrition_other] = '1'

[ nutrition_counseling ]

[ nutrition_clarify ] [ provide_meals ]

[ breastmilk_bank ]

[ food_services_clarify ]

[ blood_donation ]

Do you provide parenteral nutrition (IV) for malnourished patients?

Do you provide parenteral nutrition (IV) for other types of pediatric patients (e.g. short gut, etc.)?

If yes, what other types of patients (e.g short gut, etc.)

Do you provide nutrition counseling for non-malnourished pediatric patients?

This is a space to clarify any answers from the nutrition section.

Section Header: *Food Services*

Do you provide meals for pediatric patients?

For neonates, do you have a donor breastmilk bank?

This is a space to clarify any answers from the food services section.

Section Header: *Blood Bank*

Do you have a blood donor program at your institution?

yesno

1. Yes
2. No

yesno

1. Yes
2. No text

yesno

1. Yes
2. No

notes

yesno

1. Yes
2. No

yesno

1. Yes
2. No notes

yesno

1. Yes
2. No

379

[ blood_presecreen ]

Regardless of where blood comes from (i.e. from your institution,

checkbox

380

[ blood_presecreen_other ]

Show the field ONLY if: [blood_presecreen(7)] = '1'

or elsewhere) which of the following is the blood prescreened for?

Please specify "other"

1

2

3

4

5

6

7

text

blood_presecreen 1

blood_presecreen 2

blood_presecreen 3

blood_presecreen 4

blood_presecreen 5

blood_presecreen 6

blood_presecreen 7

HIV

Hepatitis B Hepatitis C HTVL

RPR CMV

Other

381

382

383

384

385

386

387

388

389

390

391

[ national_blood_donor ]

[ blood_bank ]

[ blood_supply ]

[ ped_blood_units ]

[ transfusion_guidelines ]

[ transfusion_guide_attach ]

Show the field ONLY if: [transfusion_guidelines] = '1'

[ transfusion_guide_attach_2 ]

Show the field ONLY if: [transfusion_guide_attach] <> ''

[ transfusion_guide_attach_3 ]

Show the field ONLY if: [transfusion_guide_attach_2] <

> ''

[ transfusion_guide_url ]

Show the field ONLY if: [transfusion_guidelines] = '1'

[ transfusion_guide_url_2 ]

Show the field ONLY if: [transfusion_guide_url] <> ''

[ transfusion_guide_url_3 ]

Show the field ONLY if: [transfusion_guide_url_2] <> ''

Do you have a national blood donor program?

Do you have a blood bank in your facility/network?

Average daily supply of blood (number of units) available for your institution

Does the blood bank stock or prepare pediatric units?

Does your institution have guidelines for who should/should not be transfused?

If yes, please attach transfusion guidelines here or URL below.

Additional file

Additional file

Paste URL here:

Additional URL

Additional URL

yesno

1. Yes
2. No

yesno

1. Yes
2. No text

yesno

1. Yes
2. No

yesno

1. Yes
2. No file

file

file

text

file

file

| 392 | [ blood_products ] | Is your institution able to provide the following other blood products?  *check all that apply* | checkbox | | | | | |
| --- | --- | --- | --- | --- | --- | --- | --- | --- |
|  |  |  | 1 | blood_products 1 | | | Intravenous Gamma Globulin (IVIG) | |
|  |  |  | 2 | blood_products 2 | | | Immune Serum Immunoglobulin | |
|  |  |  | 3 | blood_products 3 | | | Fresh Frozen Plasma | |
|  |  |  | 4 | blood_products 4 | | | Clotting Factors | |
|  |  |  | 5 | blood_products 5 | | | Platelets | |
|  |  |  | 6 | blood_products 6 | | | Other | |
| 393 | [ blood_products_other ]  Show the field ONLY if: [blood_products(6)] = '1' | If you selected "other," please specify | text | | | | | |
| 394 | [ blood_bank_clarify ] | This is a space to clarify any answers from the blood bank section. | notes | | | | | |
| 395 | [ toxicology_service ] | Section Header: *Toxicology Service (Poison Control Center)*  Do you have access to a toxicology service? | yesno | |  | | | |
|  |  |  | 1 | Yes |  |  |  |  |
|  |  |  | 0 | No |  |  |  |  |
| 396 | [ toxicology_service_hrs ]  Show the field ONLY if: [toxicology_service] = '1' | If yes, what are the hours of operation?  *(days, hours)* | text | | | | | |
| 397 | [ services_clarify ] | This is a space to clarify any answers from the toxicology section. | notes | | | | | |
| 398 | [ capacityservices_complete ] | Section Header: *Form Status*  Complete? | dropdown | | | | | |
|  |  |  | 0 | Incomplete | |  | | |
|  |  |  | 1 | Unverified | |  |  |  |
|  |  |  | 2 | Complete | |  |  |  |
| Instrument: Laboratory Tests (laboratory_tests)  Enabled as survey | | | | | | | | |
| 399 | [ lab_background ] | This is a space to give background on the laboratory setup in your hospital. | notes | | | | | |
| 400 | [ available_chem_tests ] | Section Header: *Panels*  Please select all of the following panels that are available | checkbox | | | | | |
|  |  |  | 1 | available_chem_tests 1 | | | | Electrolyte panel (sodium, potassium, chloride, CO2, etc.) |
|  |  |  | 2 | available_chem_tests 2 | | | | Metabolic panel (glucose, albumin, calcium, etc.) |
|  |  |  | 3 | available_chem_tests 3 | | | | Liver function panel (ALT, ALL, GGT, etc.) |
|  |  |  | 4 | available_chem_tests 4 | | | | Renal function panel (Blood Urea Nitrogen, serum creatinine, urine creatinine, urinalysis -  i.e. for blood, protein,  Nitrites, ketones, etc.) |
|  |  |  | 5 | available_chem_tests 5 | | | | Blood gas determination (e.g. pO2, pCO2, pH) |
|  |  |  | 6 | available_chem_tests 6 | | | | Hematology panel (Red blood cell count, White blood cell count, Hemoglobin, Hematocrit, Platelets) |
|  |  |  | 7 | available_chem_tests 7 | | | | Coagulation panel (Prothrombin Time (PT), Partial Thromboplastin Time (PTT)) |
| 401 | [ panels_who_pays ] | Who pays for the following tests? | descriptive | | | | | |

402

403

404

405

406

407

408

409

410

[ electrolyte_who_pays ]

Show the field ONLY if: [available_chem_tests(1)] = '1'

[ metabolic_who_pays ] Show the field ONLY if:

[available_chem_tests(2)] = '1'

[ liver_who_pays ] Show the field ONLY if:

[available_chem_tests(3)] = '1'

[ renal_who_pays ] Show the field ONLY if:

[available_chem_tests(4)] = '1'

[ blood_gas_who_pays ] Show the field ONLY if:

[available_chem_tests(5)] = '1'

[ heme_who_pays ] Show the field ONLY if:

[available_chem_tests(6)] = '1'

[ coag_who_pays ] Show the field ONLY if:

[available_chem_tests(7)] = '1'

[ in_house ]

[ electrolyte_in_house ] Show the field ONLY if:

[available_chem_tests(1)] = '1'

Electrolyte Panel

Metabolic Panel

Liver Function Panel

Renal Function Panel

Blood Gas Determination

Hematology Panel

Coagulation Panel

Can you do the following tests in-house? Electrolyte Panel

checkbox

| 1 | electrolyte_who_pays 1 | Patient |
| --- | --- | --- |
| 2 | electrolyte_who_pays 2 | Government |
| 3 | electrolyte_who_pays 3 | Private Insurance |
| 4 | electrolyte_who_pays 4 | National Insurance |

Field Annotation: Check all that apply checkbox

| 1 | metabolic_who_pays 1 | Patient |
| --- | --- | --- |
| 2 | metabolic_who_pays 2 | Government |
| 3 | metabolic_who_pays 3 | Private Insurance |
| 4 | metabolic_who_pays 4 | National Insurance |

Field Annotation: Check all that apply checkbox

| 1 | liver_who_pays 1 | Patient |
| --- | --- | --- |
| 2 | liver_who_pays 2 | Government |
| 3 | liver_who_pays 3 | Private Insurance |
| 4 | liver_who_pays 4 | National Insurance |

Field Annotation: Check all that apply checkbox

| 1 | renal_who_pays 1 | Patient |
| --- | --- | --- |
| 2 | renal_who_pays 2 | Government |
| 3 | renal_who_pays 3 | Private Insurance |
| 4 | renal_who_pays 4 | National Insurance |

Field Annotation: Check all that apply checkbox

| 1 | blood_gas_who_pays 1 | Patient |
| --- | --- | --- |
| 2 | blood_gas_who_pays 2 | Government |
| 3 | blood_gas_who_pays 3 | Private Insurance |
| 4 | blood_gas_who_pays 4 | National Insurance |

Field Annotation: Check all that apply checkbox

| 1 | heme_who_pays 1 | Patient |
| --- | --- | --- |
| 2 | heme_who_pays 2 | Government |
| 3 | heme_who_pays 3 | Private Insurance |
| 4 | heme_who_pays 4 | National Insurance |

Field Annotation: Check all that apply checkbox

| 1 | coag_who_pays 1 | Patient |
| --- | --- | --- |
| 2 | coag_who_pays 2 | Government |
| 3 | coag_who_pays 3 | Private Insurance |
| 4 | coag_who_pays 4 | National Insurance |

Field Annotation: Check all that apply descriptive

radio (Matrix)

1. Yes
2. No

411

412

413

414

415

416

417

418

419

420

421

422

423

424

425

426

427

[ metabolic_in_house ]

Show the field ONLY if: [available_chem_tests(2)] = '1'

[ liver_in_house ]

Show the field ONLY if: [available_chem_tests(3)] = '1'

[ renal_in_house ] Show the field ONLY if:

[available_chem_tests(4)] = '1'

[ blood_gas_in_house ] Show the field ONLY if:

[available_chem_tests(5)] = '1'

[ heme_in_house ] Show the field ONLY if:

[available_chem_tests(6)] = '1'

[ coag_in_house ] Show the field ONLY if:

[available_chem_tests(7)] = '1' [ commercial_labs ]

[ electrolyte_commercial ]

Show the field ONLY if: [available_chem_tests(1)] = '1'

[ metabolic_commercial ]

Show the field ONLY if: [available_chem_tests(2)] = '1'

[ liver_commercial ] Show the field ONLY if:

[available_chem_tests(3)] = '1'

[ renal_commercial ] Show the field ONLY if:

[available_chem_tests(4)] = '1'

[ blood_gas_commercial ]

Show the field ONLY if: [available_chem_tests(5)] = '1'

[ heme_commercial ] Show the field ONLY if:

[available_chem_tests(6)] = '1'

[ coag_commercial ] Show the field ONLY if:

[available_chem_tests(7)] = '1' [ microassays ]

[ electrolyte_microassays ]

Show the field ONLY if: [available_chem_tests(1)] = '1'

[ metabolic_microassays ]

Show the field ONLY if: [available_chem_tests(2)] = '1'

Metabolic Panel

Liver Function Panel

Renal Function Panel

Blood Gas Determination

Hematology Panel

Coagulation Panel

Do you ever use commercial laboratories to provide the following services?

Electrolyte Panel

Metabolic Panel

Liver Function Panel

Renal Function Panel

Blood Gas Determination

Hematology Panel

Coagulation Panel

Are assays available to perform these tests on small volumes of blood?

Electrolyte Panel

Metabolic Panel

radio (Matrix)

1. Yes
2. No

radio (Matrix)

1. Yes
2. No

radio (Matrix)

1. Yes
2. No

radio (Matrix)

1. Yes
2. No

radio (Matrix)

1. Yes
2. No

radio (Matrix)

1. Yes
2. No descriptive

radio (Matrix)

1. Yes
2. No

radio (Matrix)

1. Yes
2. No

radio (Matrix)

1. Yes
2. No

radio (Matrix)

1. Yes
2. No

radio (Matrix)

1. Yes
2. No

radio (Matrix)

1. Yes
2. No

radio (Matrix)

1. Yes
2. No descriptive

radio (Matrix)

1. Yes
2. No

radio (Matrix)

1. Yes
2. No

428

429

430

431

432

433

[ liver_microassays ]

Show the field ONLY if: [available_chem_tests(3)] = '1'

[ renal_microassays ] Show the field ONLY if:

[available_chem_tests(4)] = '1'

[ blood_gass_microassays ]

Show the field ONLY if: [available_chem_tests(5)] = '1'

[ heme_microassays ] Show the field ONLY if:

[available_chem_tests(6)] = '1'

[ coag_microassays ] Show the field ONLY if:

[available_chem_tests(7)] = '1'

[ blood_gas_det ]

Show the field ONLY if: [available_chem_tests(5)] = '1'

Liver Function Panel

Renal Function Panel

Blood Gas Determination

Hematology Panel

Coagluation Panel

Which types of blood can blood gas determination tests be performed on?

radio (Matrix)

1. Yes
2. No

radio (Matrix)

1. Yes
2. No

radio (Matrix)

1. Yes
2. No

radio (Matrix)

1. Yes
2. No

radio (Matrix)

1. Yes
2. No

radio

1. arterial
2. venuos
3. both

434

435

[ hematocrits ]

Show the field ONLY if: [available_chem_tests(6)] = '1'

[ cell_counts ]

Do you have a centrifuge to run hematocrits?

How are differential white blood cell counts performed?

yesno

1 Yes

1. No

checkbox

*check all that apply*

1. cell_counts 1
2. cell_counts 2
3. cell_counts 3
4. cell_counts 4

automated machine microscopy not available

436

[ anemia_diagnosis ]

What methods do you use to diagnose anemia?

checkbox

437

[ anemia_diagnosis_other ]

Show the field ONLY if: [anemia_diagnosis(5)] = '1'

*check all that apply*

If you selected "other", please specify

1

2

3

4

5

text

anemia_diagnosis 1

anemia_diagnosis 2

anemia_diagnosis 3

anemia_diagnosis 4

anemia_diagnosis 5

HemoCueÂ® (or similar hemoglobinometer)

Hemoglobin color scale

Direct cyanmethemoglobin method

Automated analyzer Other

438

439

[ poc_test ]

[ poc_tests_available ]

Are point-of-care (i.e. at the bedside and/or in the patient care unit) tests available?

If yes, which point-of-care tests are available?

yesno

1 Yes

1. No

checkbox

Show the field ONLY if: [poc_test] = '1'

1. poc_tests_available 1
2. poc_tests_available 2
3. poc_tests_available 3
4. poc_tests_available 4
5. poc_tests_available 5
6. poc_tests_available 6

Blood gases Electrolytes Hematology Glucose analysis Bilirubin

Other

440

441

442

443

[ anemia_diagnosis_other_2 ]

Show the field ONLY if: [poc_tests_available(6)] = '1'

[ stool_cultures ]

[ stool_cultures_in_house ]

Show the field ONLY if: [stool_cultures] = '1'

[ stool_cultures_who_pays ]

Show the field ONLY if: [stool_cultures] = '1'

If you selected "other", please specify

Section Header: *Microbiology*

Are stool cultures available?

Can you do stool cultures in-house?

Who pays for stool cultures?

*check all that apply*

text

yesno

1 Yes

1. No

yesno

1. Yes
2. No

checkbox

1. stool_cultures_who_pays 1
2. stool_cultures_who_pays 2
3. stool_cultures_who_pays 3
4. stool_cultures_who_pays 4

patient government private insurance national insurance

444

[ stool_cultures_commercial ]

Do you ever, or also use commercial and/or reference

checkbox

445

[ stool_cultures_reference ]

Show the field ONLY if: [stool_cultures_commercial(2)]

= '1'

laboratories for bacterial cultures for stool cultures ?

*check all that apply*

If you use a reference lab, do you only refer to reference labs for certain suspected conditions? If yes, please specify.

*If no or not applicable leave blank*

1

2

3

text

stool_cultures_commercial 1

stool_cultures_commercial 2

stool_cultures_commercial 3

Yes - Commerical laboratories

Yes - Reference laboratories

No

446

447

448

449

450

451

[ stool_cultures_bactec ]

Show the field ONLY if: [stool_cultures] = '1'

[ sbf_cultures ]

[ sbf_cultures_sensitivities ]

Show the field ONLY if: [sbf_cultures] = '1'

[ sbf_cultures_in_house ]

Show the field ONLY if: [sbf_cultures] = '1'

[ sbf_cultures_microscopic ]

Show the field ONLY if: [sbf_cultures] = '1'

[ sbf_cultures_who_pays ]

Show the field ONLY if: [sbf_cultures] = '1'

Do you use automated BactecÂ® or similar machines?

Are bacterial cultures on sterile body fluids (e.g. blood, spinal fluid) available?

If yes, can you provide antibiotic sensitivities?

Can you do bacterial cultures on sterile body fluids in-house?

Are microscopic exams available (gram stain)?

Who pays for bacterial cultures on sterile body fluids?

*check all that apply*

yesno

1. Yes
2. No

yesno

1. Yes
2. No

yesno

1. Yes
2. No

yesno

1. Yes
2. No

yesno

1. Yes
2. No

checkbox

1. sbf_cultures_who_pays 1
2. sbf_cultures_who_pays 2
3. sbf_cultures_who_pays 3
4. sbf_cultures_who_pays 4

patient government private insurance national insurance

452

[ sbf_cultures_commercial ]

Do you ever, or also use commercial and/or reference

checkbox

laboratories for bacterial cultures for sterile body fluids ?

*check all that apply*

- 1. sbf_cultures_commercial 1
  2. sbf_cultures_commercial 2
  3. sbf_cultures_commercial 3

Yes - Commerical laboratories

Yes - Reference laboratories

No

| 453 | [ sbf_cultures_reference ]  Show the field ONLY if: [sbf_cultures_commercial(2)] = '1' | If you use a reference lab, do you only refer to reference labs for certain suspected conditions? If yes, please specify.  *If no or not applicable leave blank* | text | | | | | |
| --- | --- | --- | --- | --- | --- | --- | --- | --- |
| 454 | [ serology_lab ] | Section Header: *Diagnostic Serology*  Do you have a diagnostic serology laboratory in-house? | yesno | |  | | | |
|  |  |  | 1 | Yes |  |  |  |  |
|  |  |  | 0 | No |  |  |  |  |
| 455 | [ available_serology_assays ]  Show the field ONLY if: [serology_lab] = '1' | If yes, which assays are available? | checkbox | | | | | |
|  |  |  | 1 | available_serology_assays 1 | | | HIV |  |
|  |  |  | 2 | available_serology_assays 2 | | | Salmonella |  |
|  |  |  | 3 | available_serology_assays 3 | | | TORCHES |  |
|  |  |  | 4 | available_serology_assays 4 | | | CMV |  |
|  |  |  | 5 | available_serology_assays 5 | | | Herpes Simplex |  |
|  |  |  | 6 | available_serology_assays 6 | | | Other |  |
| 456 | [ serology_other ]  Show the field ONLY if: [available_serology_assays(6)]  = '1' | If you selected "other," please specify | text | | | | | |
| 457 | [ serology_commercial ] | Do you ever, or also use commercial and/or reference laboratories for diagnostic serology services?  *check all that apply* | checkbox | | | | | |
|  |  |  | 1 | serology_commercial 1 | | Yes - Commerical laboratories | | |
|  |  |  | 2 | serology_commercial 2 | | Yes - Reference laboratories | | |
|  |  |  | 3 | serology_commercial 3 | | No | | |
| 458 | [ serology_commercial_which ]  Show the field ONLY if: [serology_commercial(1)] = '1' or [serology_commercial(2)] = '1' | If yes, for which serologic assays? | notes | | | | | |
| 459 | [ molecular_diagnostics ] | Section Header: *Molecular Diagnostics*  Are molecular diagnostics (i.e. PCR, etc) available in-house? | yesno | |  | | | |
|  |  |  | 1 | Yes |  |  |  |  |
|  |  |  | 0 | No |  |  |  |  |

460

[ available_molecular_diag ]

If yes, which molecular diagnostics are available?

checkbox

461

Show the field ONLY if: [molecular_diagnostics] = '1'

[ available_molec_diag_other ]

Show the field ONLY if: [available_molecular_diag(25)]

= '1'

If you selected other, please specify

1

2

3

4

5

6

7

8

9

10

11

12

13

14

15

16

17

18

19

20

21

22

23

24

25

text

available_molecular_diag 1

available_molecular_diag 2

available_molecular_diag 3

available_molecular_diag 4

available_molecular_diag 5

available_molecular_diag 6

available_molecular_diag 7

available_molecular_diag 8

available_molecular_diag 9

available_molecular_diag 10

available_molecular_diag 11

available_molecular_diag 12

available_molecular_diag 13

available_molecular_diag 14

available_molecular_diag 15

available_molecular_diag 16

available_molecular_diag 17

available_molecular_diag 18

available_molecular_diag 19

available_molecular_diag 20

available_molecular_diag 21

available_molecular_diag 22

available_molecular_diag 23

available_molecular_diag 24

available_molecular_diag 25

Cytomegalovirus qualitative PCR

Cytomegalovirus quantitative PCR

Adenovirus qualitativ PCR

Adenovirus quantitative PCR

Hepatitis B virus qualitative PCR

Hepatitis B virus quantitative PCR

Hepatitis C virus qualitative PCR

Hepatitis C virus quantitative PCR

HIV-1 qualitative DNA PCR

HIV-1 quantitative RN PCR

HIV-2 qualitative DNA PCR

HIV-2 quantitative RN PCR

Methicillin Resistant Staphylococcus Aure PCR Screen

Respiratory Pathoge PCR Panel

Tuberculosis Nucleic Acid Amplification

Herpes simplex-1 vir qualitative DNA PCR

Herpes simplex-2 vir qualitative DNA PCR

Varicella Zoster Virus qualitative DNA PCR

Meningitis/Encephali PCR Panel, CSF

Gastrointestinal Pathogen PCR Panel

Legionella Urine Antigen

Streptococcus pneumoniae

Urine Antigen ELISA assay

Cryptococcal antigen ELISA assa

Other

462

[ molec_diag_who_pays ]

Show the field ONLY if: [molecular_diagnostics] = '1'

Who pays for molecular diagnostics?

*check all that apply*

checkbox

1. molec_diag_who_pays 1
2. molec_diag_who_pays 2
3. molec_diag_who_pays 3
4. molec_diag_who_pays 4

patient government private insurance national insurance

463

[ molec_diag_commercial ]

Do you ever, or also use commercial and/or reference

checkbox

laboratories for molecular diagnostic services?

*check all that apply*

- 1. molec_diag_commercial 1
  2. molec_diag_commercial 2
  3. molec_diag_commercial 3

Yes - Commerical laboratories

Yes - Reference laboratories

No

464

465

466

467

468

469

470

471

472

[ malaria_diagnosis ]

[ malaria_who_pays ] Show the field ONLY if:

[malaria_diagnosis(1)] = '1' or [malaria_diagnosis(2)] = '1'

[ mal_microscopic_who_pays ]

Show the field ONLY if: [malaria_diagnosis(1)] = '1'

[ mal_rapid_test_who_pays ]

Show the field ONLY if: [malaria_diagnosis(2)] = '1'

[ malaria_in_house ] Show the field ONLY if:

[malaria_diagnosis(1)] = '1' or [malaria_diagnosis(2)] = '1'

[ mal_microscopic_in_house ]

Show the field ONLY if: [malaria_diagnosis(1)] = '1'

[ mal_rapid_test_in_house ]

Show the field ONLY if: [malaria_diagnosis(2)] = '1'

[ malaria_commercial ]

[ mal_microscopic_commercial

Section Header: *Malaria Diagnosis*

Which of the following methods are available for diagnosis of malaria?

Who pays for the following malaria diagnostics?

Microscopic diagnosis

Rapid test

Can you do the following tests for malaria in-house?

Microscopic diagnosis

Rapid test

Do you ever, or also use commercial and/or reference laboratories for these services?

Microscopic diagnosis

checkbox

1 malaria_diagnosis 1 Microscopic diagnosis

2 malaria_diagnosis 2 Rapid test (HRP2 or pLDH) descriptive

checkbox

| 1 | mal_microscopic_who_pays 1 | Patient |
| --- | --- | --- |
| 2 | mal_microscopic_who_pays 2 | Government |
| 3 | mal_microscopic_who_pays 3 | Private Insurance |
| 4 | mal_microscopic_who_pays 4 | National Insurance |

Field Annotation: check all that apply checkbox

| 1 | mal_rapid_test_who_pays 1 | Patient |
| --- | --- | --- |
| 2 | mal_rapid_test_who_pays 2 | Government |
| 3 | mal_rapid_test_who_pays 3 | Private Insurance |
| 4 | mal_rapid_test_who_pays 4 | National Insurance |

Field Annotation: check all that apply descriptive

radio (Matrix)

1 Yes

0 No

radio (Matrix)

1 Yes

0 No descriptive

checkbox

] 1 mal_microscopic_commercial 1

2 mal_microscopic_commercial 2

3 mal_microscopic_commercial 3

Yes - Commercial Laboratories

Yes - Reference Laboratories

No

473

[ mal_rapid_test_commercial ]

Rapid Test

checkbox

1. mal_rapid_test_commercial 1
2. mal_rapid_test_commercial 2
3. mal_rapid_test_commercial 3

Yes - Commercial Laboratories

Yes - Reference Laboratories

No

474

[ tb_diagnosis ]

Section Header: *Tuberculosis (TB) Diagnosis*

checkbox

Which of the following non-radiologic methods are available for diagnosis of TB?

1 tb_diagnosis 1

2 tb_diagnosis 2

3 tb_diagnosis 3

4 tb_diagnosis 4

Microscopic exam (Acid Fast stain, Ziehl-Neelsen test) of body fluids (sputum, cerebral spinal, gastric aspirate, pleural and/ or peritoneal fluids)

Culture for TB

Polymerase chain reaction (i.e. GeneXpert, Multiplex PCR assays, etc.)

Interferon gamma release assay

475

476

477

478

479

480

481

[ tb_culture_sensitivities ]

Show the field ONLY if: [tb_diagnosis(2)] = '1'

[ tb_who_pays ]

[ tb_microscopic_who_pays ]

Show the field ONLY if: [tb_diagnosis(1)] = '1'

[ tb_culture_who_pays ]

Show the field ONLY if: [tb_diagnosis(2)] = '1'

[ tb_pcr_who_pays ]

Show the field ONLY if: [tb_diagnosis(3)] = '1'

[ tb_ifngamma_who_pays ]

Show the field ONLY if: [tb_diagnosis(4)] = '1'

[ tb_commercial ]

For a TB culture, can you provide antibiotic sensitivities?

Who pays for the following tests for TB diagnosis? Microscopic exam

Culture for TB

PCR

Interferon gamma release

Do you ever, or also use commercial and or reference laboratories for these services? (If yes check all that apply, or check no)

yesno

1 Yes

0 No

descriptive checkbox

| 1 | tb_microscopic_who_pays 1 | Patient |
| --- | --- | --- |
| 2 | tb_microscopic_who_pays 2 | Government |
| 3 | tb_microscopic_who_pays 3 | Private Insurance |
| 4 | tb_microscopic_who_pays 4 | National Insurance |

Field Annotation: check all that apply checkbox

| 1 | tb_culture_who_pays 1 | Patient |
| --- | --- | --- |
| 2 | tb_culture_who_pays 2 | Government |
| 3 | tb_culture_who_pays 3 | Private Insurance |
| 4 | tb_culture_who_pays 4 | National Insurance |

Field Annotation: check all that apply checkbox

| 1 | tb_pcr_who_pays 1 | Patient |
| --- | --- | --- |
| 2 | tb_pcr_who_pays 2 | Government |
| 3 | tb_pcr_who_pays 3 | Private Insurance |
| 4 | tb_pcr_who_pays 4 | National Insurance |

Field Annotation: check all that apply checkbox

| 1 | tb_ifngamma_who_pays 1 | Patient |
| --- | --- | --- |
| 2 | tb_ifngamma_who_pays 2 | Government |
| 3 | tb_ifngamma_who_pays 3 | Private Insurance |
| 4 | tb_ifngamma_who_pays 4 | National Insurance |

Field Annotation: check all that apply descriptive

| 482 | [ tb_microscopic_commercial ] | Microscopic exam | checkbox | | | | | | | |
| --- | --- | --- | --- | --- | --- | --- | --- | --- | --- | --- |
|  |  |  | 1 | tb_microscopic_commercial 1 | | | | | | Yes - Commercial Laboratories |
|  |  |  | 2 | tb_microscopic_commercial 2 | | | | | | Yes - Reference Laboratories |
|  |  |  | 3 | tb_microscopic_commercial 3 | | | | | | No |
| 483 | [ tb_culture_commercial ] | Culture for TB | checkbox | | | | | | | |
|  |  |  | 1 | tb_culture_commercial 1 | | | | Yes - Commercial Laboratories | | |
|  |  |  | 2 | tb_culture_commercial 2 | | | | Yes - Reference Laboratories | | |
|  |  |  | 3 | tb_culture_commercial 3 | | | | No | | |
| 484 | [ tb_pcr_commercial ] | PCR | checkbox | | | | | | | |
|  |  |  | 1 | tb_pcr_commercial 1 | | | Yes - Commercial Laboratories | | | |
|  |  |  | 2 | tb_pcr_commercial 2 | | | Yes - Reference Laboratories | | | |
|  |  |  | 3 | tb_pcr_commercial 3 | | | No | | | |
| 485 | [ tb_ifngamma_commercial ] | Interferon gamma release | checkbox | | | | | | | |
|  |  |  | 1 | tb_ifngamma_commercial 1 | | | | | Yes - Commercial Laboratories | |
|  |  |  | 2 | tb_ifngamma_commercial 2 | | | | | Yes - Reference Laboratories | |
|  |  |  | 3 | tb_ifngamma_commercial 3 | | | | | No | |
| 486 | [ lab_clarify ] | Section Header: *Clariications*  This is a space to clarify any answers from the laboratory tests section. | notes | | | | | | | |
| 487 | [ laboratory_tests_complete ] | Section Header: *Form Status*  Complete? | dropdown | | | | | | | |
|  |  |  | 0 | Incomplete | |  | | | | |
|  |  |  | 1 | Unverified | |  |  |  |  |  |
|  |  |  | 2 | Complete | |  |  |  |  |  |
| Instrument: Clinical Staff (clinical_staff)  Enabled as survey | | | | | | | | | | |
| 488 | [ staff_background ] | This is a space to give background on the professional staff (i.e. medical doctors and nurses) in your hospital.  *e.g. may deine terms used in your country such as "registrar" or "resident"* | notes | | | | | | | |
| 489 | [ doctors ] | Section Header: *Non-surgeon Medical Doctors*  Numbers of non-surgeon medical doctors that care for children (including doctors with adult and pediatric training) | descriptive | | | | | | | |
| 490 | [ n_doctors_1 ] | Number of Consultants, Fellows, and Senior Registrars | text | | | | | | | |
| 491 | [ n_doctors_2 ] | Number of Residents/Registrars (i.e. those doing MMed training) | text | | | | | | | |
| 492 | [ n_doctors_3 ] | Number of Medical officers/Senior House Officers (SHO)/Government Medical Officers (GMO) | text | | | | | | | |
| 493 | [ n_doctors_4 ] | Number of Interns (in the whole internship program with which your hospital is affiliated) | text | | | | | | | |
| 494 | [ n_doctors_5 ] | Number of Interns stationed in your hospital (if dedicated children's hospital) or in the pediatric ward at any given time. | text | | | | | | | |
| 495 | [ adequate_doctors ] | Is the number of non-surgeon medical doctors adequate for your current needs? | yesno | |  | | | | | |
|  |  |  | 1 | Yes |  |  |  |  |  |  |
|  |  |  | 0 | No |  |  |  |  |  |  |
| 496 | [ addl_doctors_needed ]  Show the field ONLY if: [adequate_doctors] = '0' | If no, how many additional non-surgeon medical doctors are needed? | text (integer) | | | | | | | |
| 497 | [ n_pediatricians ] | Pediatricians: Number of medical doctors with general pediatric training (i.e. someone who has done an MMed in pediatrics or equivalent training program in pediatrics) | text | | | | | | | |

| 498 | [ adequate_pediatricians ] | Is the number of pediatricians adequate for your current needs? | yesno | |  | |
| --- | --- | --- | --- | --- | --- | --- |
|  |  |  | 1 | Yes |  |  |
|  |  |  | 0 | No |  |  |
| 499 | [ addl_pediatricians_needed ]  Show the field ONLY if: [adequate_pediatricians] = '0' | If no, how many additional pediatricians are needed? | text (integer) | | | |
| 500 | [ pediatric_subspecialty ] | Pediatric sub-specialists: Number of pediatricians with formal subspecialty training | descriptive | | | |
| 501 | [ pediatric_subspecialties ] | In which of the following subspecialties are there trained pediatricians in your hospital? | checkbox | | | |
|  |  |  | 1 | pediatric_subspecialties 1 | | allergy |
|  |  |  | 2 | pediatric_subspecialties 2 | | cardiology |
|  |  |  | 3 | pediatric_subspecialties 3 | | critical care |
|  |  |  | 4 | pediatric_subspecialties 4 | | community pediatrics |
|  |  |  | 5 | pediatric_subspecialties 5 | | dermatology |
|  |  |  | 6 | pediatric_subspecialties 6 | | development |
|  |  |  | 7 | pediatric_subspecialties 7 | | emergency medicine |
|  |  |  | 8 | pediatric_subspecialties 8 | | endocrinology |
|  |  |  | 9 | pediatric_subspecialties 9 | | gastroenterology |
|  |  |  | 10 | pediatric_subspecialties 10 | | hematology- oncology |
|  |  |  | 11 | pediatric_subspecialties 11 | | infectious disease |
|  |  |  | 12 | pediatric_subspecialties 12 | | neonatalogy |
|  |  |  | 13 | pediatric_subspecialties 13 | | nephrology |
|  |  |  | 14 | pediatric_subspecialties 14 | | neurology |
|  |  |  | 15 | pediatric_subspecialties 15 | | pulmonology |
|  |  |  | 16 | pediatric_subspecialties 16 | | rheumatology |
|  |  |  | 17 | pediatric_subspecialties 17 | | other |
| 502 | [ n_subspecialists ] | For each of the subspecialties you indicated, please specify the number of doctors | descriptive | | | |
| 503 | [ n_ss_allergy ]  Show the field ONLY if: [pediatric_subspecialties(1)] = '1' | Pediatric Allergy | text | | | |
| 504 | [ n_ss_cardiology ]  Show the field ONLY if: [pediatric_subspecialties(2)] = '1' | Pediatric Cardiology | text | | | |
| 505 | [ n_ss_critical_care ]  Show the field ONLY if: [pediatric_subspecialties(3)] = '1' | Pediatric Critical Care | text | | | |
| 506 | [ n_ss_cp ]  Show the field ONLY if: [pediatric_subspecialties(4)] = '1' | Community Pediatrics | text | | | |
| 507 | [ n_ss_derm ]  Show the field ONLY if: [pediatric_subspecialties(5)] = '1' | Pediatric Dermatology | text | | | |
| 508 | [ n_ss_dev ]  Show the field ONLY if: [pediatric_subspecialties(6)] = '1' | Development | text | | | |

| 509 | [ n_ss_emerg ]  Show the field ONLY if: [pediatric_subspecialties(7)] = '1' | Pediatric Emergency Medicine | text | | |
| --- | --- | --- | --- | --- | --- |
| 510 | [ n_ss_endocrinology ]  Show the field ONLY if: [pediatric_subspecialties(8)] = '1' | Pediatric Endocrinology | text | | |
| 511 | [ n_ss_gastro ]  Show the field ONLY if: [pediatric_subspecialties(9)] = '1' | Pediatric Gastroenterology | text | | |
| 512 | [ n_ss_heme_onc ]  Show the field ONLY if: [pediatric_subspecialties(10)] = '1' | Pediatric Hematology-Oncology | text | | |
| 513 | [ n_ss_id ]  Show the field ONLY if: [pediatric_subspecialties(11)] = '1' | Pediatric Infectious Disease | text | | |
| 514 | [ n_ss_neonate ]  Show the field ONLY if: [pediatric_subspecialties(12)] = '1' | Neonatology | text | | |
| 515 | [ n_ss_neph ]  Show the field ONLY if: [pediatric_subspecialties(13)] = '1' | Pediatric Nephrology | text | | |
| 516 | [ n_ss_neuro ]  Show the field ONLY if: [pediatric_subspecialties(14)] = '1' | Pediatric Neurology | text | | |
| 517 | [ n_ss_pulm ]  Show the field ONLY if: [pediatric_subspecialties(15)] = '1' | Pediatric Pulmonology | text | | |
| 518 | [ n_ss_rheum ]  Show the field ONLY if: [pediatric_subspecialties(16)] = '1' | Pediatric Rheumatology | text | | |
| 519 | [ ped_subspecialties_other ]  Show the field ONLY if: [pediatric_subspecialties(17)] = '1' | If you selected "other," please specify pediatric subspecialty and number of doctors | notes | | |
| 520 | [ adequate_ped_ss ] | Is the number of pediatric subspecialists adequate for your current needs? | yesno | |  |
|  |  |  | 1 | Yes |  |
|  |  |  | 0 | No |  |
| 521 | [ addl_ped_ss ]  Show the field ONLY if: [adequate_ped_ss] = '0' | If no, how many additional subspecialists are needed and in what areas? | notes | | |
| 522 | [ n_specialty_doctors ] | Specialists: Number of non-pediatrician doctors with formal specialty training that can also care for children (for example an adult cardiologist that also treats children) | descriptive | | |

| 523 | [ specialties ] | In which of the following specialties are there trained doctors that also care for children? | checkbox | | | |
| --- | --- | --- | --- | --- | --- | --- |
|  |  |  | 1 | specialties 1 | allergy |  |
|  |  |  | 2 | specialties 2 | cardiology |  |
|  |  |  | 3 | specialties 3 | critical care |  |
|  |  |  | 4 | specialties 4 | dermatology |  |
|  |  |  | 5 | specialties 5 | emergency medicine |  |
|  |  |  | 6 | specialties 6 | endocrinology |  |
|  |  |  | 7 | specialties 7 | gastroenterology |  |
|  |  |  | 8 | specialties 8 | hematology-oncology |  |
|  |  |  | 9 | specialties 9 | infectious disease |  |
|  |  |  | 10 | specialties 10 | nephrology |  |
|  |  |  | 11 | specialties 11 | neurology |  |
|  |  |  | 12 | specialties 12 | perinatology |  |
|  |  |  | 13 | specialties 13 | pulmonology |  |
|  |  |  | 14 | specialties 14 | rheumatology |  |
|  |  |  | 15 | specialties 15 | other |  |
| 524 | [ n_specialists ] | For each of the specialties you indicated, please specify the number of doctors | descriptive | | | |
| 525 | [ n_s_allergy ]  Show the field ONLY if: [specialties(1)] = '1' | Allergy | text | | | |
| 526 | [ n_s_cardiology ]  Show the field ONLY if: [specialties(2)] = '1' | Cardiology | text | | | |
| 527 | [ n_s_critical_care ]  Show the field ONLY if: [specialties(3)] = '1' | Critical care | text | | | |
| 528 | [ n_s_dermatology ]  Show the field ONLY if: [specialties(4)] = '1' | Dermatology | text | | | |
| 529 | [ n_s_emergency ]  Show the field ONLY if: [specialties(5)] = '1' | Emergency medicine | text | | | |
| 530 | [ n_s_endocrinology ]  Show the field ONLY if: [specialties(6)] = '1' | Endocrinology | text | | | |
| 531 | [ n_s_gastro ]  Show the field ONLY if: [specialties(7)] = '1' | Gastroenterology | text | | | |
| 532 | [ n_s_heme_onc ]  Show the field ONLY if: [specialties(8)] = '1' | Hematology-Oncology | text | | | |
| 533 | [ n_s_id ]  Show the field ONLY if: [specialties(9)] = '1' | Infectious Disease | text | | | |
| 534 | [ n_s_neph ]  Show the field ONLY if: [specialties(10)] = '1' | Nephrology | text | | | |
| 535 | [ n_s_neuro ]  Show the field ONLY if: [specialties(11)] = '1' | Neurology | text | | | |
| 536 | [ n_s_perinatal ]  Show the field ONLY if: [specialties(12)] = '1' | Perinatology | text | | | |

| 537 | [ n_s_pulm ]  Show the field ONLY if: [specialties(13)] = '1' | Pulmonology | text | | |
| --- | --- | --- | --- | --- | --- |
| 538 | [ n_s_rheum ]  Show the field ONLY if: [specialties(14)] = '1' | Rheumatology | text | | |
| 539 | [ specialties_other ]  Show the field ONLY if: [specialties(15)] = '1' | If you selected "other," please specify specialties and number of doctors | notes | | |
| 540 | [ adequate_specialists ] | Is the number of specialists that care for children adequate for your current needs? | yesno | |  |
|  |  |  | 1 | Yes |  |
|  |  |  | 0 | No |  |
| 541 | [ addl_specialists_needed ]  Show the field ONLY if: [adequate_specialists] = '0' | If no, for which of the specialties would it be beneficial to have (additional) staff? | notes | | |
| 542 | [ adolescent_specialists ] | Adolescent medicine: Number of specialists with formal training in adolescent medicine | text (integer) | | |
| 543 | [ adequate_adolescent ] | Is the number of adolescent medicine specialists adequate for your current needs? | yesno | |  |
|  |  |  | 1 | Yes |  |
|  |  |  | 0 | No |  |
| 544 | [ addl_adolescent ]  Show the field ONLY if: [adequate_adolescent] = '0' | If no, how many additional adolescent medicine specialists are needed? | text (integer) | | |
| 545 | [ doctors_clarify ] | This is a space to clarify any answers from the "Non-Surgeon Medical Doctors" section | notes | | |
| 546 | [ n_nurses ] | Section Header: *Nurses*  Nurses: Number of nurses that care for children (including nurses with adult and pediatric training) | text | | |
| 547 | [ adequate_nurses ] | Is the number of nurses adequate for your current needs? | yesno | |  |
|  |  |  | 1 | Yes |  |
|  |  |  | 0 | No |  |
| 548 | [ addl_nurses ]  Show the field ONLY if: [adequate_nurses] = '0' | If no, how many additional nurses are needed? | text (integer) | | |
| 549 | [ n_ped_nurses ] | Pediatric nurses: Number of nurses with additional formal training in pediatric nursing  *If this category of staff does not exist in your country, type "NA". Otherwise type in a number (e.g. type "0" if this category of staff is used but there are currently none in your hospital)* | text | | |
| 550 | [ adequate_ped_nurses ]  Show the field ONLY if: [n_ped_nurses] >= '0' | Is the number of pediatric nurses adequate for your current needs? | yesno | |  |
|  |  |  | 1 | Yes |  |
|  |  |  | 0 | No |  |
| 551 | [ addl_ped_nurses ]  Show the field ONLY if: [adequate_ped_nurses] = '0' | If no, how many additional pediatric nurses are needed? | text (integer) | | |
| 552 | [ n_nn_nurses ] | Neonatal nurses: Number of nurses with additional formal training in neonatal nursing  *If this category of staff does not exist in your country, type: NA* | text | | |
| 553 | [ adq_nn_nurses ]  Show the field ONLY if: [n_nn_nurses] >= '0' | Is the number of neonatal nurses adequate for your current needs? | yesno | |  |
|  |  |  | 1 | Yes |  |
|  |  |  | 0 | No |  |
| 554 | [ addl_nn_nurses ]  Show the field ONLY if: [adq_nn_nurses] = '0' | If no, how many additional neonatal nurses are needed? | text | | |

| 1 | Yes |
| --- | --- |
| 0 | No |

| 1 | np_specialize 1 | Pediatrics |
| --- | --- | --- |
| 2 | np_specialize 2 | Neonatology |
| 3 | np_specialize 3 | Other |

| 1 | Yes |
| --- | --- |
| 0 | No |

| 1 | Yes |
| --- | --- |
| 0 | No |

| 555 | [ n_2_p_in_nicu ] | What is the nurse-to-patient ratio in in the NICU and/or neonatal high-care unit?  *(number of nurses:number of patients)* | text |
| --- | --- | --- | --- |
| 556 | [ n_cc_nurses ] | Critical Care nurses: Number of nurses with additional formal training in critical care nursing  *If this category of staff does not exist in your country, type "NA". Otherwise type in a number (e.g. type "0" if this category of staff is used but there are currently none in your hospital)* | text |
| 557 | [ adq_cc_nurses ]  Show the field ONLY if: [n_cc_nurses] >= '0' | Is the number of critical care nurses adequate for your current needs? | yesno |
| 558 | [ addl_cc_nurses ]  Show the field ONLY if: [adq_cc_nurses] = '0' | If no, how many additional critical care nurses are needed? | text |
| 559 | [ nurse_patient_ratio_icu ] | What is the nurse-to-patient ratio in the ICU?  *(number of nurses:number of patients)* | text |
| 560 | [ n_nps ] | Number of Nurse Practitioners that care for children  *If this category of staff does not exist in your country, type "NA". Otherwise type in a number (e.g. type "0" if this category of staff is used but there are currently none in your hospital)* | text |
| 561 | [ np_specialize ]  Show the field ONLY if: [n_nps] >= '0' | In which areas do the nurse practitioners specialize? (if applicable) | checkbox |
| 562 | [ n_ped_nps ]  Show the field ONLY if: [np_specialize(1)] = '1' | Number of pediatric nurse practitioners | text |
| 563 | [ n_nn_nps ]  Show the field ONLY if: [np_specialize(2)] = '1' | Number of neonatal nurse practitioners | text |
| 564 | [ np_specialize_other ]  Show the field ONLY if: [np_specialize(3)] = '1' | If you selected other, please specify specialty and number of doctors. | text |
| 565 | [ adq_nps ]  Show the field ONLY if: [n_nps] >= '0' | Is the number of nurse practitioners adequate for your current needs? | yesno |
| 566 | [ addl_nps ]  Show the field ONLY if: [adq_nps] = '0' | If no, how many additional nurses practitioners are needed and in which areas? | text |
| 567 | [ nurses_clarify ] | This is a space to clarify any answers from the "Nurses" section | notes |
| 568 | [ surgeons ] | Section Header: *Surgeons and Surgical Staff*  General surgeons: Total number of general surgeons that also operate on children (including surgeons with adult and pediatric training) | descriptive |
| 569 | [ n_surgeons_1 ] | Number of Consultants, Fellows and Senior Registrars | text  Custom alignment: RH |
| 570 | [ n_surgeons_2 ] | Number of Residents/Registrars (i.e. those doing an MMed in surgery) | text  Custom alignment: RH |
| 571 | [ n_surgeons_3 ] | Number of Medical officers/Senior House officers (SHO)/Government Medical Officer (GMO) | text  Custom alignment: RH |
| 572 | [ adequate_surgeons ] | Is the number of general surgeons adequate for your current pediatric needs? | yesno |
| 573 | [ addl_surgeons ]  Show the field ONLY if: [adequate_surgeons] = '0' | If no, how many additional general surgeons are needed? | text (integer) |
| 574 | [ n_ped_surgeons ] | Pediatric Surgeons: Number of surgeons with additional formal training in pediatric surgery | text |

| 575 | [ adequate_ped_surgeons ] | Is the number of pediatric surgeons adequate for your current needs? | yesno | |  | |
| --- | --- | --- | --- | --- | --- | --- |
|  |  |  | 1 | Yes |  |  |
|  |  |  | 0 | No |  |  |
| 576 | [ addl_ped_surgeons ]  Show the field ONLY if: [adequate_ped_surgeons] = '0' | If no, how many additional surgeons with formal training in pediatric surgery are needed? | text (integer) | | | |
| 577 | [ ped_ss_surgeons ] | Pediatric subspecialist surgeons: Number of surgeons with additional formal training in a pediatric subspecialty surgical field. | descriptive | | | |
| 578 | [ p_surgeon_specialties ] | In which of the following subspecialties are the pediatric surgeons trained? | checkbox | | | |
|  |  |  | 1 | p_surgeon_specialties 1 | | cardiothoracic surgery |
|  |  |  | 2 | p_surgeon_specialties 2 | | neonatal surgery |
|  |  |  | 3 | p_surgeon_specialties 3 | | neurological surgery |
|  |  |  | 4 | p_surgeon_specialties 4 | | oncology |
|  |  |  | 5 | p_surgeon_specialties 5 | | ophthalmic surgery |
|  |  |  | 6 | p_surgeon_specialties 6 | | oral and maxillofacial surgery |
|  |  |  | 7 | p_surgeon_specialties 7 | | orthopedic surgery |
|  |  |  | 8 | p_surgeon_specialties 8 | | otorhinolaryngology |
|  |  |  | 9 | p_surgeon_specialties 9 | | plastic surgery |
|  |  |  | 10 | p_surgeon_specialties 10 | | trauma surgery |
|  |  |  | 11 | p_surgeon_specialties 11 | | vascular surgery |
|  |  |  | 12 | p_surgeon_specialties 12 | | other |
| 579 | [ n_surg_subspecialists ] | For each of the surgical subspecialties you indicated, please specify the number of doctors | descriptive | | | |
| 580 | [ n_p_cardio_surg ]  Show the field ONLY if: [p_surgeon_specialties(1)] = '1' | Pediatric cardiothoracic surgeons | text | | | |
| 581 | [ n_neonatal_surg ]  Show the field ONLY if: [p_surgeon_specialties(2)] = '1' | Neonatal surgeons | text | | | |
| 582 | [ n_p_neurosurg ]  Show the field ONLY if: [p_surgeon_specialties(3)] = '1' | Pediatric neurosurgeons | text | | | |
| 583 | [ n_p_onc_surg ]  Show the field ONLY if: [p_surgeon_specialties(4)] = '1' | Pediatric surgical oncologists | text | | | |
| 584 | [ n_p_opth_surg ]  Show the field ONLY if: [p_surgeon_specialties(5)] = '1' | Pediatric ophthalmic surgeons | text | | | |
| 585 | [ n_p_oral_surg ]  Show the field ONLY if: [p_surgeon_specialties(6)] = '1' | Pediatric oral and maxillofacial surgeons | text | | | |
| 586 | [ n_p_ortho_surg ]  Show the field ONLY if: [p_surgeon_specialties(7)] = '1' | Pediatric orthopedic surgeons | text | | | |
| 587 | [ n_p_ent ]  Show the field ONLY if: [p_surgeon_specialties(8)] = '1' | Pediatric otorhinolaryngologists | text | | | |
| 588 | [ n_p_plastic_surg ]  Show the field ONLY if: [p_surgeon_specialties(9)] = '1' | Pediatric plastic surgeons | text | | | |

| 589 | [ n_p_trauma_surg ]  Show the field ONLY if: [p_surgeon_specialties(10)] = '1' | Pediatric trauma surgeons | text | | | |
| --- | --- | --- | --- | --- | --- | --- |
| 590 | [ n_p_vascular_surg ]  Show the field ONLY if: [p_surgeon_specialties(11)] = '1' | Pediatric vascular surgeons | text | | | |
| 591 | [ n_p_surgeon_ss_other ]  Show the field ONLY if: [p_surgeon_specialties(12)] = '1' | If you selected "other," please specify pediatric surgical subspecialty and number of surgeons. | notes | | | |
| 592 | [ adequate_ped_ss_surgeons ] | Is the number of pediatric subspecialist surgeons adequate for your current needs? | yesno | |  | |
|  |  |  | 1 | Yes |  |  |
|  |  |  | 0 | No |  |  |
| 593 | [ addl_ped_ss_surgeons ]  Show the field ONLY if: [adequate_ped_ss_surgeons] = '0' | If no, how many additional pediatric surgeons are needed and in which subspecialties? | notes | | | |
| 594 | [ specialist_surgeons ] | Specialist surgeons: Number of non-pediatric surgeons with specialty training (i.e. not general surgeons) that operate on children (for example, an adult cardiothoracic surgeon that also operates on children) | descriptive | | | |
| 595 | [ surgeon_specialties ] | In which of the following specialties are the surgeons trained? | checkbox | | | |
|  |  |  | 1 | surgeon_specialties 1 | | cardiothoracic surgery |
|  |  |  | 2 | surgeon_specialties 2 | | colon and rectal surgery |
|  |  |  | 3 | surgeon_specialties 3 | | gynecology and obstetrics |
|  |  |  | 4 | surgeon_specialties 4 | | neurological surgery |
|  |  |  | 5 | surgeon_specialties 5 | | oncology |
|  |  |  | 6 | surgeon_specialties 6 | | ophthalmic surgery |
|  |  |  | 7 | surgeon_specialties 7 | | oral and maxillofacial surgery |
|  |  |  | 8 | surgeon_specialties 8 | | orthopedic surgery |
|  |  |  | 9 | surgeon_specialties 9 | | otorhinolaryngology |
|  |  |  | 10 | surgeon_specialties 10 | | plastic surgery |
|  |  |  | 11 | surgeon_specialties 11 | | trauma surgery |
|  |  |  | 12 | surgeon_specialties 12 | | vascular surgery |
|  |  |  | 13 | surgeon_specialties 13 | | other |
| 596 | [ n_surg_specialists ] | For each of the surgical specialties you indicated, please specify the number of surgeons | descriptive | | | |
| 597 | [ n_cardio_surg ]  Show the field ONLY if: [surgeon_specialties(1)] = '1' | Cardiothoracic surgeons | text | | | |
| 598 | [ n_colon_surg ]  Show the field ONLY if: [surgeon_specialties(2)] = '1' | Colon and rectal surgeons | text | | | |
| 599 | [ n_gyn_surg ]  Show the field ONLY if: [surgeon_specialties(3)] = '1' | Gynecologic and obstetric surgeons | text | | | |
| 600 | [ n_neurosurg ]  Show the field ONLY if: [surgeon_specialties(4)] = '1' | Neurosurgeons | text | | | |

| 1 | Yes |
| --- | --- |
| 0 | No |

| 1 | Yes |
| --- | --- |
| 0 | No |

| 1 | Yes |
| --- | --- |
| 0 | No |

| 601 | [ n_onc_surg ]  Show the field ONLY if: [surgeon_specialties(5)] = '1' | Surgical oncologists | text |
| --- | --- | --- | --- |
| 602 | [ n_opth_surg ]  Show the field ONLY if: [surgeon_specialties(6)] = '1' | Ophthalmic surgeons | text |
| 603 | [ n_oral_surg ]  Show the field ONLY if: [surgeon_specialties(7)] = '1' | Oral and maxillofacial surgeons | text |
| 604 | [ n_ortho_surg ]  Show the field ONLY if: [surgeon_specialties(8)] = '1' | Orthopedic surgeons | text |
| 605 | [ n_ent ]  Show the field ONLY if: [surgeon_specialties(9)] = '1' | Otorhinolaryngologists | text |
| 606 | [ n_plastic_surg ]  Show the field ONLY if: [surgeon_specialties(10)] = '1' | Plastic surgeons | text |
| 607 | [ n_trauma_surg ]  Show the field ONLY if: [surgeon_specialties(11)] = '1' | Trauma surgeons | text |
| 608 | [ n_vascular_surg ]  Show the field ONLY if: [surgeon_specialties(12)] = '1' | Vascular surgeons | text |
| 609 | [ n_surg_specialty_other ]  Show the field ONLY if: [surgeon_specialties(13)] = '1' | If you selected "other," please specify surgical specialty and number of surgeons. | notes |
| 610 | [ adequate_specialty_surg ] | Is the number of specialty surgeons adequate for your current pediatric needs? | yesno |
| 611 | [ addl_specialty_surg ]  Show the field ONLY if: [adequate_specialty_surg] = '0' | If no, how many additional surgeons are needed and in which surgical specialties? | notes |
| 612 | [ n_surgical_nurses ] | Surgical/scrub nurses: Number of nurses with formal training in surgical nursing that participate in surgeries for children  *If this category of staff does not exist in your country, type: NA. Otherwise type in a number (e.g. type "0" if this category of staff is used but there are currently none in your hospital)* | text |
| 613 | [ adequate_surg_nurses ]  Show the field ONLY if: [n_surgical_nurses] >= '0' | Is the number of surgical nurses adequate for your current needs? | yesno |
| 614 | [ addl_surg_nurses ]  Show the field ONLY if: [adequate_surg_nurses] = '0' | If no, how many additional surgical nurses are needed? | text |
| 615 | [ n_surgical_techs ] | Surgical technologists: Number of non-doctor, non-nurse healthcare workers (i.e. surgical technologists) that participate in surgeries for children  *If this category of staff does not exist in your country, type "NA". Otherwise type in a number (e.g. type "0" if this category of staff is used but there are currently none in your hospital)* | text |
| 616 | [ adequate_surg_techs ]  Show the field ONLY if: [n_surgical_techs] >= '0' | Is the number of surgical technologists adequate for your current needs? | yesno |
| 617 | [ addl_surg_techs ]  Show the field ONLY if: [adequate_surg_techs] = '0' | If no, how many additional surgical technologists are needed? | text |

618

619

620

621

622

623

624

625

626

627

628

629

630

631

632

633

634

635

636

[ surgeons_clarify ]

[ anesthesiologists ]

[ n_anesthiesiologists_1 ] [ n_anesthiesiologists_2 ]

[ n_anesthiesiologists_3 ]

[ adequate_anesthesiologists ]

[ addl_anesthesiologists ]

Show the field ONLY if: [adequate_anesthesiologists]

= '0'

[ n_ped_anesthesiologists ]

[ adequate_ped_anesthes ]

[ addl_ped_anesthes ] Show the field ONLY if:

[adequate_ped_anesthes] = '0' [ n_nurse_anesth ]

[ adequate_nurse_anesth ]

Show the field ONLY if: [n_nurse_anesth] >= '0'

[ addl_nurse_anesth ] Show the field ONLY if:

[adequate_nurse_anesth] = '0' [ n_anesth_techs ]

[ adequate_anesth_techs ]

Show the field ONLY if: [n_anesth_techs] >= '0'

[ addl_anesth_techs ] Show the field ONLY if:

[adequate_anesth_techs] = '0' [ anesthesiologists_clarify ]

[ n_pharmacists ]

[ adq_pharmacists ]

This is a space to clarify any answers from the "Surgeons and Surgical Staff" section

Section Header: *Anesthesiologists and Anesthesia Staff*

Anesthesiologists: Number of anesthesiologists that participate in surgeries for children

Number of Consultants, Fellows and Senior Registrars.

Number of Residents/Registrars (i.e. those doing an MMed in anesthesiology)

Number of Medical officers/Senior House officers (SHO)/Government Medical Officer (GMO) that provide anesthesia

Is the number of anesthesiologists adequate for your current pediatric needs?

If no, how many additional anesthesiologists are needed?

Pediatric anesthesiologists: Number of anesthesiologists with additional formal training in pediatric anesthesiology

Is the number of pediatric anesthesiologists adequate for your current needs?

If no, how many additional pediatric anesthesiologists are needed?

Nurse anesthetists: Number of nurse anesthetists (nurses with formal training in anesthesiology) that participate in surgeries for children

*If this category of staff does not exist in your country, type "NA". Otherwise type in a number (e.g. type "0" if this category of staff is used but there are currently none in your hospital)*

Is the number of nurse anesthetists adequate for your current needs?

If no, how many additional nurse anesthetists are needed?

Anesthesia technicians: Number of non-doctor, non-nurse healthcare workers (i.e. anesthesia technicians) that participate in surgeries for infants and children

*If this category of staff does not exist in your country, type "NA". Otherwise type in a number (e.g. type "0" if this category of staff is used but there are currently none in your hospital)*

Is the number of anesthesia technicians adequate for your current needs?

If no, how many additional anesthesia technicians are needed?

This is a space to clarify any answers from the "Anesthesiologists and Anesthesia Staff" section

Section Header: *Pharmacists and Pharmacy Staff*

Number of pharmacists that provide services for children

Is the number of pharmacists adequate for your current needs?

notes

descriptive

text text

text

yesno

| 1 | Yes |
| --- | --- |
| 0 | No |

text

text

yesno

| 1 | Yes |
| --- | --- |
| 0 | No |

text

text

yesno

| 1 | Yes |
| --- | --- |
| 0 | No |

text

text

yesno

| 1 | Yes |
| --- | --- |
| 0 | No |

text

notes

text

yesno

| 1 | Yes |
| --- | --- |
| 0 | No |

637

638

639

640

641

642

643

644

645

646

647

648

649

650

651

652

653

654

[ addl_pharmacists ]

Show the field ONLY if: [adq_pharmacists] = '0'

[ n_pharm_techs ]

[ adq_pharm_techs ]

Show the field ONLY if: [n_pharm_techs] >= '0'

[ addl_pharm_techs ]

Show the field ONLY if: [adq_pharm_techs] = '0'

[ clinical_pharm ]

[ pharmacists_clarify ]

[ radiologists ]

[ n_radiologists_1 ] [ n_radiologists_2 ]

[ n_radiologists_3 ]

[ adequate_radiologists ]

[ addl_radiologists ] Show the field ONLY if:

[adequate_radiologists] = '0' [ n_ped_radiologists ]

[ adequate_ped_radiologists ]

[ addl_ped_radiologists ] Show the field ONLY if:

[adequate_ped_radiologists] = '0'

[ radiologists_trained_to ]

[ n_radiology_techs ]

[ adequate_radiology_techs ]

Show the field ONLY if: [n_radiology_techs] >= '0'

If no, how many additional pharmacists are needed?

Number of pharmacy technicians (pharmacy assistants) that provide services for children

*If this category of staff does not exist in your country, type "NA". Otherwise type in a number (e.g. type "0" if this category of staff is used but there are currently none in your hospital)*

Is the number of pharmacy technicians adequate for your current needs?

If no, how many additional pharmacy technicians are needed?

Do you have a clinical pharmacy service where the pharmacy provides the patient/family with information and the drugs being prescribed and/or assists doctors in addressing the drug needs of the patient?

This is a space to clarify any answers from the "Pharmacists and Pharmacy Staff" section.

Section Header: *Radiologists and Radiology Staff*

Radiologists: Number of doctors with training in radiology that provide services for children

Number of Consultants, Fellows and Senior Registrars

Number of Residents/Registrars (i.e. those doing an MMed in radiology)

Number of Medical officers/Senior House officers (SHO)/Government Medical Officer (GMO) that provide radiology services

Is the number of radiologists adequate for your current pediatric needs?

If no, how many additional radiologists are needed?

Number of radiologists with additional formal training in pediatric radiology

Is the number of pediatric radiologists adequate for your current needs?

If no, how many additional pediatric radiologists are needed?

Are the radiologists trained to:

Radiographers: Number of radiology technicians/radiographers that provide services for children

*If this category of staff does not exist in your country, type "NA". Otherwise type in a number (e.g. type "0" if this category of staff is used but there are currently none in your hospital)*

Is the number of radiology technicians/radiographers adequate for your current needs?

text

text

yesno

| 1 | Yes |
| --- | --- |
| 0 | No |

text

yesno

| 1 | Yes |
| --- | --- |
| 0 | No |

notes

descriptive

text text

text

yesno

| 1 | Yes |
| --- | --- |
| 0 | No |

text

text

yesno

| 1 | Yes |
| --- | --- |
| 0 | No |

text

checkbox

| 1 | radiologists_trained_to 1 | reduce intussusceptions |
| --- | --- | --- |
| 2 | radiologists_trained_to 2 | diagnose midgut volvulus |
| 3 | radiologists_trained_to 3 | diagnose pyloric stenosis |

text

yesno

| 1 | Yes |
| --- | --- |
| 0 | No |

655

656

657

658

659

660

661

662

663

664

665

666

667

668

669

670

671

672

673

[ addl_radiology_techs ]

Show the field ONLY if: [adequate_radiology_techs] = '0'

[ n_ped_radiology_techs ]

[ adequate_ped_radiol_techs ]

Show the field ONLY if: [n_ped_radiology_techs] >= '0'

[ addl_ped_radiology_techs ]

Show the field ONLY if: [adequate_ped_radiol_techs] = '0'

[ radiologists_clarify ]

[ n_dentists ]

[ adequate_dentists ]

[ addl_dentists ]

Show the field ONLY if: [adequate_dentists] = '0'

[ n_ped_dentists ]

[ adequate_ped_dentists ]

[ addl_ped_dentists ] Show the field ONLY if:

[adequate_ped_dentists] = '0' [ n_dental_surg ]

[ adequate_dental_surg ]

[ addl_dental_surg ] Show the field ONLY if:

[adequate_dental_surg] = '0' [ n_dental_nurses ]

[ adequate_dental_nurses ]

Show the field ONLY if: [n_dental_nurses] >= '0'

[ addl_dental_nurses ] Show the field ONLY if:

[adequate_dental_nurses] = '0' [ facial_trauma ]

[ dentists_clarify ]

If no, how many additional radiology technicians/radiographers are needed?

Number of radiology technicians/radiographers with additional formal training in pediatric radiology

*If this category of staff does not exist in your country, type "NA". Otherwise type in a number (e.g. type "0" if this category of staff is used but there are currently none in your hospital)*

Is the number of radiography technicians/radiographers with additional formal training in pediatric radiology adequate for your current needs?

If no, how many additional pediatric radiology technicians/radiographers are needed?

This is a space to clarify any answers from the "Radiologists and Radiology Staff" section.

Section Header: *Dentists, Dental Surgeons, and Dental Staff*

Number of dentists that care for children

Is the number of dentists adequate for your current pediatric needs?

If no, how many additional dentists are needed?

Number of dentists with formal training in pediatric dentistry

Is the number of dentists with formal training in pediatric dentistry adequate for your current needs?

If no, how many additional dentists with formal training in pediatric dentistry are needed?

Number of dental surgeons that care for children

Is the number of dental surgeons adequate for your current pediatric needs?

If no, how many additional dental surgeons are needed?

Number of nurses with formal training in dental care that care for children

*If this category of staff does not exist in your country, type "NA". Otherwise type in a number (e.g. type "0" if this category of staff is used but there are currently none in your hospital)*

Is the number of nurses with formal training in dental care adequate for your current pediatric needs?

If no, how many additional nurses with formal training in dental care are needed?

Do dental services deal with facial trauma?

This is a place to clarify any answers from the "Dentists, Dental Surgeons, and Dental Staff" section.

text

text

yesno

| 1 | Yes |
| --- | --- |
| 0 | No |

text

notes

text

yesno

| 1 | Yes |
| --- | --- |
| 0 | No |

text

text yesno

| 1 | Yes |
| --- | --- |
| 0 | No |

text

text yesno

| 1 | Yes |
| --- | --- |
| 0 | No |

text

text

yesno

| 1 | Yes |
| --- | --- |
| 0 | No |

text

yesno

| 1 | Yes |
| --- | --- |
| 0 | No |

notes

674

675

676

677

678

679

680

681

682

683

684

685

686

687

688

[ n_phys_nutritionists ]

[ adequate_phys_nutr ]

Show the field ONLY if: [n_phys_nutritionists] >= '0'

[ addl_phys_nutritionists ]

Show the field ONLY if: [adequate_phys_nutr] = '0'

[ n_ped_phys_nutr ]

[ adequate_ped_phys_nutr ]

Show the field ONLY if: [n_ped_phys_nutr] >= '0'

[ addl_ped_phys_nutr ] Show the field ONLY if:

[adequate_ped_phys_nutr] = '0'

[ n_nondoc_nutr ]

[ adequate_nondoc_nutr ]

Show the field ONLY if: [n_nondoc_nutr] >= '0'

[ addl_nondoc_nutr ] Show the field ONLY if:

[adequate_nondoc_nutr] = '0' [ n_nondoc_ped_nutr ]

[ adequate_nondoc_ped_nutr ]

Show the field ONLY if: [n_nondoc_ped_nutr] >= '0'

[ addl_nondoc_ped_nutr ]

Show the field ONLY if: [adequate_nondoc_ped_nutr]

= '0'

[ nutritionists_clarify ]

[ n_clinical_officers ]

[ adequate_clinical_officers ]

Show the field ONLY if: [n_clinical_officers] >= '0'

Section Header: *Nutrition Specialists*

Number of doctors that provide nutrition care for children (i.e. doctors who care for children with malnutrition and other nutrition issues)

*If this category of staff does not exist in your country, type "NA". Otherwise type in a number (e.g. type "0" if this category of staff is used but there are currently none in your hospital)*

Is the number of doctors that provide nutrition care for children adequate for your current needs?

If no, how many additional doctors are needed?

Number of doctors with formal training in pediatric nutrition

*If this category of staff does not exist in your country, type "NA". Otherwise type in a number (e.g. type "0" if this category of staff is used but there are currently none in your hospital)*

Is the number of doctors with formal training in pediatric nutrition adequate for your current needs?

If no, how many additional doctors with formal training in pediatric nutrition are needed?

Number of non-doctor staff with formal training in nutrition that care for children (i.e. dietitians, nutritionists)

*If this category of staff does not exist in your country, type "NA". Otherwise type in a number (e.g. type "0" if this category of staff is used but there are currently none in your hospital)*

Is the number of staff adequate for your current needs?

If no, how many additional non-doctor nutrition staff members are needed?

Number of non-doctor staff with formal training in pediatric nutrition

*If this category of staff does not exist in your country, type "NA". Otherwise type in a number (e.g. type "0" if this category of staff is used but there are currently none in your hospital)*

Is the number of staff members adequate for your current needs?

If no, how many additional non-doctor pediatric nutrition staff members are needed?

This is a space to clarify any answers from the "Nutrition Specialists" section

Section Header: *Clinical Oicers (Doctor/Physician Assistants) and Other Clinical Staff*

Number of clinical officers that care for children

*If this category of staff does not exist in your country, type "NA". Otherwise type in a number (e.g. type "0" if this category of staff is used but there are currently none in your hospital)*

Is the number of clinical officers adequate for your current needs?

text

yesno

| 1 | Yes |
| --- | --- |
| 0 | No |

text

text

yesno

| 1 | Yes |
| --- | --- |
| 0 | No |

text

text

yesno

| 1 | Yes |
| --- | --- |
| 0 | No |

text

text

yesno

| 1 | Yes |
| --- | --- |
| 0 | No |

text

notes

text

yesno

| 1 | Yes |
| --- | --- |
| 0 | No |

689

690

691

692

693

694

695

[ addl_clinical_officers ]

Show the field ONLY if: [adequate_clinical_officers] = '0'

[ n_ped_clinical_officers ]

[ adequate_ped_cos ] Show the field ONLY if:

[n_ped_clinical_officers] >= '0'

[ addl_ped_clinical_officers ]

Show the field ONLY if: [adequate_ped_cos] = '0'

[ other_staff ]

[ training ]

[ training_programs ]

Show the field ONLY if: [training] = '1'

If no, how many additional clinical officers are needed?

Number of clinical officers with formal training in pediatrics

*If this category of staff does not exist in your country, type "NA". Otherwise type in a number (e.g. type "0" if this category of staff is used but there are currently none in your hospital)*

Is the number of clinical officers with formal training in pediatrics adequate for your current needs?

If no, how many additional clinical officers with formal training in pediatrics are needed?

Are there other categories of clinical staff that have not been mentioned? If yes, please comment.

Section Header: *Training Programs*

Do you have postgraduate medical training programs (i.e. MMed programs)?

Which of the following programs are available

text (integer)

text

yesno

1 Yes

1. No

text (integer)

notes

yesno

1. Yes
2. No

checkbox

1. training_programs 1 internship
2. training_programs 2 residency

696

697

698

[ intern_training_duration ]

Show the field ONLY if: [training_programs(1)] = '1'

[ res_training_duration ]

Show the field ONLY if: [training_programs(2)] = '1'

[ fellow_training_duration ]

Show the field ONLY if: [training_programs(3)] = '1'

What is the duration of the internship program (in months)?

What is the duration of the residency program (in months)?

What is the duration of the fellowship program (in months)?

3

text

text

text

training_programs 3

fellowship

699

700

701

702

703

704

705

[ doc_training_programs ]

Show the field ONLY if: [training] = '1'

[ nursing_training ]

[ nurse_training_programs ]

Show the field ONLY if: [nursing_training] = '1'

[ nurse_training_duration ]

Show the field ONLY if: [nursing_training] = '1'

[ dentist_training ]

[ dentist_training_enrolled ]

Show the field ONLY if: [dentist_training] = '1'

[ dentist_training_duration ]

Show the field ONLY if: [dentist_training] = '1'

Which pediatric-specific programs (i.e. MMed in Pediatrics, others) are offered, and how many are enrolled in each? *program name (number enrolled)*

Do you have nursing training programs?

If yes, which programs and how many are enrolled?

*program name (number enrolled)*

If yes, what is the duration (in months)?

Do you have dentist training programs?

If yes, how many are enrolled?

If yes, what is the duration (in months)?

notes

yesno

1 Yes

1. No notes

text

yesno

1. Yes
2. No text

text

706

707

708

709

710

711

712

713

714

[ dho_training ]

[ dho_training_enrolled ]

Show the field ONLY if: [dho_training] = '1'

[ dho_training_duration ]

Show the field ONLY if: [dho_training] = '1'

[ training_clarify ]

[ staff_surge ]

[ diversion ]

[ diversion_depts ]

Show the field ONLY if: [diversion] > 0

[ staff_clarify ]

[ clinical_staff_complete ]

Do you have clinical dental health officer training programs

If yes, how many are enrolled?

If yes, what is the duration (in months)?

This is a space to clarify any answers from the "Training Programs" section.

Section Header: *Staff Surge Capacity*

Irrespective of bed capacity, in the event that a catastrophic situation exceeds your staff capacity for children, what strategies can be used?

*(e.g. diversion, pull additional staff from other services, contact retired staff, make do with existing resources, etc.)*

How many times in the past month have you had to put pediatric services on diversion (closed to new admissions because the facility has reached the maximum number of patients that can be cared for in the inpatient wards and ICU or the Emergency (Casualty) area)?

If diversion has occurred, in what departments?

This is a space to clarify any answers from the "Staff Surge Capacity" section.

Section Header: *Form Status*

yesno

1. Yes
2. No text

text

notes

notes

text

text

notes

dropdown

Complete?

- 1. Incomplete
  2. Unverified
  3. Complete

Instrument: Supply Chain and Waste Removal (supply_chain_and_waste_removal)  Enabled as survey

715

716

717

718

[ adequate_supplies ]

[ supplies_shortages ]

Show the field ONLY if: [adequate_supplies] = '0'

[ reuse_supplies ]

[ top_reused_supplies ]

Show the field ONLY if: [reuse_supplies] = '1'

Section Header: *Medical Supplies*

Do you have adequate medical supplies to meet your current pediatric needs?

If no, please specify shortages.

Do you reuse disposable supplies?

If yes, what are the top three most commonly reused supplies?

yesno

1. Yes
2. No notes

yesno

1. Yes
2. No notes

719

[ shortages ]

Which of the following supplies have you experienced a shortage

checkbox

of in the past year?

1. shortages 1
2. shortages 2
3. shortages 3
4. shortages 4
5. shortages 5
6. shortages 6
7. shortages 7
8. shortages 8
9. shortages 9

Sterile surgical gloves Saline

Recommended pediatric vaccines

Commonly used antibiotics (penicillin, ampicillin, vancomycin, nafcillin, gentamycin, amikacin, cephalothin, cefotaxime, etc.)

Insulin

Anti-malarial drugs Oxygen

Syringes and/or needles appropriate for pediatric use

Endotracheal tubes appropriate for infants and children

720

721

722

723

724

725

726

727

728

729

730

[ shortages_s_or_f ]

[ gloves_shortage_cause ]

Show the field ONLY if: [shortages(1)] = '1'

[ saline_shortage_cause ]

Show the field ONLY if: [shortages(2)] = '1'

[ vaccines_shortage_cause ]

Show the field ONLY if: [shortages(3)] = '1'

[ antibiotics_shortage_cause ]

Show the field ONLY if: [shortages(4)] = '1'

[ insulin_shortage_cause ]

Show the field ONLY if: [shortages(5)] = '1'

[ antimalar_shortage_cause ]

Show the field ONLY if: [shortages(6)] = '1'

[ oxygen_shortage_cause ]

Show the field ONLY if: [shortages(7)] = '1'

[ needles_shortage_cause ]

Show the field ONLY if: [shortages(8)] = '1'

[ et_tubes_shortage_cause ]

Show the field ONLY if: [shortages(9)] = '1'

[ heat_shortage ]

For each of the following, please specify whether the shortage was due to lack of financial resources to purchase the supply, or unavailability from the supplier (check all that apply).

Sterile surgical gloves

Saline

Recommended pediatric vaccines

Commonly used antibiotics

Insulin

Anti-malarial drugs

Oxygen

Syringes and/or needles appropriate for pediatric use

Endotracheal tubes appropriate for infants and children

Have you experienced supply shortages during periods of extreme heat?

descriptive

checkbox

1. gloves_shortage_cause 1 Finances
2. gloves_shortage_cause 2 Supply

checkbox

1. saline_shortage_cause 1 Finances
2. saline_shortage_cause 2 Supply

checkbox

1. vaccines_shortage_cause 1 Finances
2. vaccines_shortage_cause 2 Supply

checkbox

1. antibiotics_shortage_cause 1 Finances
2. antibiotics_shortage_cause 2 Supply

checkbox

1. insulin_shortage_cause 1 Finances
2. insulin_shortage_cause 2 Supply

checkbox

1. antimalar_shortage_cause 1 Finances
2. antimalar_shortage_cause 2 Supply

checkbox

1. oxygen_shortage_cause 1 Finances
2. oxygen_shortage_cause 2 Supply

checkbox

1. needles_shortage_cause 1 Finances
2. needles_shortage_cause 2 Supply

checkbox

1. et_tubes_shortage_cause 1 Finances
2. et_tubes_shortage_cause 2 Supply

yesno

1. Yes

0 No

731

[ heat_shortage_supplies ]

If yes, of which of the following supplies did you experience a

checkbox

732

Show the field ONLY if: [heat_shortage] = '1'

[ heat_shortage_other ] Show the field ONLY if:

[heat_shortage_supplies(4)] = '1'

shortage?

If you selected other, please specify.

1

2

3

4

text

heat_shortage_supplies 1

heat_shortage_supplies 2

heat_shortage_supplies 3

heat_shortage_supplies 4

Gloves

IV catheters IV fluids Other

733

734

[ blackouts ]

[ blackout_frequency ]

Section Header: *Electricity and Water Supply*

Does your facility experience blackouts?

If yes, how often does your facility experience blackouts?

yesno

1 Yes

1. No

radio

Show the field ONLY if: [blackouts] = '1'

1. daily
2. weekly
3. monthly
4. infrequently
5. never

735

736

[ generators ]

[ generator_frequency ]

Does your facility have emergency generators?

If yes, during a blackout, how often are they used?

yesno

1 Yes

1. No

radio

Show the field ONLY if: [generators] = '1'

1. always
2. sometimes
3. rarely
4. never

737

738

739

740

[ power_fluct ]

[ power_fluct_explain ]

Show the field ONLY if: [power_fluct] = '1'

[ water_interruption ]

[ water_interrupt_frequency ]

Does your facility experience other fluctuations in power supply (e.g. power drops, surges, brownouts)?

If yes, please explain type of fluctuation and frequency (daily, weekly, monthly, infrequently, never)

Does your facility experience interruptions of its water supply?

If yes, how often are the interruptions of water supply?

yesno

1 Yes

1. No notes

yesno

1. Yes
2. No

radio

Show the field ONLY if: [water_interruption] = '1'

1. daily
2. weekly
3. monthly
4. infrequently
5. never

741

742

743

[ waste_removal ]

[ waste_removal_sops ]

[ waste_sop_attach_1 ]

Show the field ONLY if: [waste_removal_sops] = '1'

Section Header: *Waste Removal*

What is your system for removal of medical waste? Are there SOPs for removal of medical waste?

If yes, please attach the document here. If SOP is available online, insert the URL below.

notes

yesno

1 Yes

0 No file

744

745

746

747

748

749

[ waste_sop_attach_2 ]

Show the field ONLY if: [waste_sop_attach_1] <> ''

[ waste_sop_attach_3 ]

Show the field ONLY if: [waste_sop_attach_2] <> ''

[ waste_sop_url_1 ]

Show the field ONLY if: [waste_removal_sops] = '1'

[ waste_sop_url_2 ]

Show the field ONLY if: [waste_sop_url_1] <> ''

[ waste_sop_url_3 ]

Show the field ONLY if: [waste_sop_url_2] <> ''

[ supply_chain_and_waste_rem oval_complete ]

Additional file

Additional file

Paste the URL here:

Additional URL

Additional URL

Section Header: *Form Status*

Complete?

file

file

text

text

text

dropdown

1. Incomplete

Instrument: Programs/Committees (programscommittees)  Enabled as survey

1. Unverified
2. Complete

750

751

752

753

754

755

[ committees_background ]

[ ipc_programs ]

[ ipc_resources ]

Show the field ONLY if: [ipc_programs] = '1'

[ ipc_budget_line ]

Show the field ONLY if: [ipc_resources] = '1'

[ n_infection_preventionists ]

Show the field ONLY if: [ipc_programs] = '1'

[ preventionist_titles ]

Show the field ONLY if: [n_infection_preventionists] > '0'

This is a space to describe the setup of Infection Prevention and Control (IPC), Healthcare-Associated Infection (HCAI), Antimicrobial Stewardship (AMS), Quality Patient Safety, Workforce health and safety, disaster response, and equipment maintenance programs in your hospital. You may include the names of the committees that deal with these issues and indicate if any of the responsibilities are combined in a shared committee (e.g. a committee that is responsible for both HCAI monitoring and AMS).

Section Header: *Infection prevention and control (IPC)*

Do you have a formal IPC program?

Does your hospital provide fiscal and human resource support for maintaining the IPC program?

If yes, is there a budget line for this program?

Number of infection preventionists (e.g. if there is an outbreak, how many trained people are available to investigate)

What are the titles/roles of the infection preventionists?

notes

yesno

1. Yes
2. No

yesno

1. Yes
2. No

yesno

1. Yes
2. No

text (integer)

notes

| 756 | [ ipc_staff ]  Show the field ONLY if: [ipc_programs] = '1' | What type of staff are involved in the program?  *check all that apply* | checkbox | | | | | |
| --- | --- | --- | --- | --- | --- | --- | --- | --- |
|  |  |  | 1 | ipc_staff 1 | | doctors | |  |
|  |  |  | 2 | ipc_staff 2 | | nurses | |  |
|  |  |  | 3 | ipc_staff 3 | | pharmacists | |  |
|  |  |  | 4 | ipc_staff 4 | | microbiologists | |  |
|  |  |  | 5 | ipc_staff 5 | | environmental health | |  |
|  |  |  | 6 | ipc_staff 6 | | administrators | |  |
|  |  |  | 7 | ipc_staff 7 | | maintenance staff | |  |
|  |  |  | 8 | ipc_staff 8 | | laboratory personnel | |  |
|  |  |  | 9 | ipc_staff 9 | | other | |  |
| 757 | [ ipc_staff_other ]  Show the field ONLY if: [ipc_staff(9)] = '1' | If you selected "other," please specify. | text | | | | | |
| 758 | [ ipc_leader ]  Show the field ONLY if: [ipc_programs] = '1' | Is there a designated leader for the IPC program? | yesno | |  | | | |
|  |  |  | 1 | Yes |  |  |  |  |
|  |  |  | 0 | No |  |  |  |  |
| 759 | [ ipc_leader_creds ]  Show the field ONLY if: [ipc_leader] = '1' | If yes, what are the leader's credentials?  *(i.e. pediatric infectious disease doctor)* | text | | | | | |
| 760 | [ ipc_leader_compensation ]  Show the field ONLY if: [ipc_leader] = '1' | How is the IPC program leader compensated for activities related to this committee? | checkbox | | | | | |
|  |  |  | 1 | ipc_leader_compensation 1 | | | additional salary support | |
|  |  |  | 2 | ipc_leader_compensation 2 | | | relieved of other responsibilities so that they can lead the IPC program | |
|  |  |  | 3 | ipc_leader_compensation 3 | | | IPC responsibilities are added to their routine responsibilities without additional compensation | |
|  |  |  | 4 | ipc_leader_compensation 4 | | | other | |
| 761 | [ ipc_compensation_other ]  Show the field ONLY if: [ipc_leader_compensation(4)]  = '1' | If you selected other, please specify. | text | | | | | |
| 762 | [ written_ipc_guidelines ] | Are there written infection control policies and procedures? | yesno | |  | | | |
|  |  |  | 1 | Yes |  |  |  |  |
|  |  |  | 0 | No |  |  |  |  |
| 763 | [ written_ipc_guide_access ] | Are you able to obtain the written infection control policies and procedures? | yesno | |  | | | |
|  |  |  | 1 | Yes |  |  |  |  |
|  |  |  | 0 | No |  |  |  |  |
| 764 | [ ipc_policy_attach_1 ]  Show the field ONLY if: [written_ipc_guide_access] = '1' | If yes, please attach the document here. If available online, insert the URL below. | file | | | | | |
| 765 | [ ipc_policy_attach_2 ]  Show the field ONLY if: [ipc_policy_attach_1] <> '' | Additional file | file | | | | | |
| 766 | [ ipc_policy_attach_3 ]  Show the field ONLY if: [ipc_policy_attach_2] <> '' | Additional file | file | | | | | |
| 767 | [ ipc_policy_url_1 ]  Show the field ONLY if: [written_ipc_guide_access] = '1' | Paste the URL here: | text | | | | | |

768

769

770

771

772

773

774

775

776

777

778

779

780

781

782

783

784

[ ipc_policy_url_2 ]

Show the field ONLY if: [ipc_policy_url_1] <> ''

[ ipc_policy_url_3 ]

Show the field ONLY if: [ipc_policy_url_2] <> ''

[ hand_hygiene ]

[ hand_hygiene_annual ]

Show the field ONLY if: [hand_hygiene] = '1'

[ written_hand_hygiene ]

[ hand_policy_attach ] Show the field ONLY if:

[written_hand_hygiene] = '1' [ hand_policy_url ]

Show the field ONLY if: [written_hand_hygiene] = '1'

[ ppe_training ]

[ ppe_annual ]

Show the field ONLY if: [hand_hygiene] = '1'

[ ppe_competency_docs ]

[ ipc_effectiveness ]

[ hcai_combined ]

Show the field ONLY if: [ipc_programs] = '1'

[ hcai_program ]

Show the field ONLY if: [hcai_combined] <> '1'

[ hcai_resources ]

Show the field ONLY if: [hcai_program] = '1'

[ hcai_resources_budget ]

Show the field ONLY if: [hcai_resources] = '1'

[ n_hcai_preventionists ]

Show the field ONLY if: [hcai_program] = '1'

[ hcai_preventionist_titles ]

Show the field ONLY if: [n_hcai_preventionists] > '0'

Additional URL

Additional URL

Does your facility have hand hygiene training program?

Is formal hand hygiene training provided at least annually?

Are written hand hygiene policies and procedures available?

If yes, please attach the document here. If available online, insert the URL below.

Paste the URL here:

Does the hospital have a competency-based training program for use of personal protective equipment (PPE)?

Is PPE training provided at least annually?

Does the hospital maintain current documentation of PPE competency for all personnel who use PPE?

This space may be used to comment on the IPC program (effectiveness or other clarifications)

Section Header: *Healthcare-associated infections (HCAI)*

Is HCAI monitoring a combined responsibility of the committee described above?

*answer no if this is not a dedicated function of the committee previously described or if there is a separate body responsible for monitoring HCAIs*

Does your hospital have a formal program to monitor HCAIs?

Does your hospital provide fiscal and human resource support for maintaining the HCAI program?

If yes, is there a budget line for this program?

Number of HCAI preventionists (e.g. if there is an outbreak, how many trained people are available to investigate)

What are the titles/roles of the HCAI preventionists?

text

text

yesno

1. Yes
2. No

yesno

1. Yes
2. No

yesno

1. Yes
2. No file

text

yesno

1. Yes
2. No

yesno

1. Yes
2. No

yesno

1. Yes
2. No notes

yesno

1. Yes
2. No

yesno

1. Yes
2. No

yesno

1. Yes
2. No

yesno

1. Yes
2. No text

notes

785

[ hcai_staff ]

What type of staff are involved in the program?

checkbox

786

Show the field ONLY if: [hcai_program] = '1'

[ hcai_staff_other ]

Show the field ONLY if: [hcai_staff(9)] = '1'

*check all that apply*

If you selected "other," please specify.

1

2

3

4

5

6

7

8

9

text

hcai_staff 1

hcai_staff 2

hcai_staff 3

hcai_staff 4

hcai_staff 5

hcai_staff 6

hcai_staff 7

hcai_staff 8

hcai_staff 9

doctors nurses pharmacists

microbiologists environmental health administrators maintenance staff laboratory personnel other

787

788

789

[ hcai_leader ]

Show the field ONLY if: [hcai_program] = '1'

[ hcai_leader_creds ]

Show the field ONLY if: [hcai_leader] = '1'

[ hcai_leader_compensation ]

Is there a designated leader for the HCAI program?

If yes, what are the leader's credentials?

*(i.e. pediatric infectious disease doctor)*

How is the HCAI program leader compensated for activities

yesno

1. Yes
2. No text

checkbox

790

Show the field ONLY if: [hcai_leader] = '1'

[ hcai_compensation_other ]

Show the field ONLY if: [hcai_leader_compensation(4)]

= '1'

related to this committee?

If you selected other, please specify.

1

2

3

4

text

hcai_leader_compensation 1

hcai_leader_compensation 2

hcai_leader_compensation 3

hcai_leader_compensation 4

additional salary support

relieved of other responsibilities so that they can lead the HCAI program

HCAI

responsibilities are added to their routine responsibilities without additional compensation

other

791

792

793

794

[ travel_history ]

[ mdro_system ]

[ infection_commun_accept ]

[ infection_commun_transfer ]

Is travel and occupational history is included as part of admission and triage protocols?

Does the hospital have a system to identify/flag patients with targeted multidrug-resistant organisms (MDROs) UPON READMISSION so appropriate precautions can be applied?

Does the hospital have a system in place for within-facility communication of infectious status and isolation needs of patients prior to ACCEPTING from other facilities?

Does the hospital have a system in place for within-facility communication of infectious status and isolation needs of patients prior to TRANSFER TO other facilities?

yesno

1. Yes
2. No

yesno

1. Yes
2. No

yesno

1. Yes
2. No

radio

1. Yes
2. No
3. Not Applicable

795

796

797

798

799

800

801

802

803

804

805

806

807

[ hcai_policy_attach_1 ]

Show the field ONLY if: [hcai_program] = '1'

[ hcai_policy_attach_2 ]

Show the field ONLY if: [hcai_policy_attach_1] <> ''

[ hcai_policy_attach_3 ]

Show the field ONLY if: [hcai_policy_attach_2] <> ''

[ hcai_policy_url_1 ]

Show the field ONLY if: [hcai_program] = '1'

[ hcai_policy_url_2 ]

Show the field ONLY if: [hcai_policy_url_1] <> ''

[ hcai_policy_url_3 ]

Show the field ONLY if: [hcai_policy_url_2] <> ''

[ hcai_effectiveness ]

[ ams_combined ] Show the field ONLY if:

[ipc_programs] = '1' or [hcai_pr ogram] = '1'

[ ams_combined_with ]

Show the field ONLY if: [ams_combined] = '1'

[ ams_program ]

Show the field ONLY if: [ams_combined] <> '1'

[ ams_resources ]

Show the field ONLY if: [ams_program] = '1'

[ ams_resources_budget ]

Show the field ONLY if: [ams_resources] = '1'

[ ams_staff ]

Please attach recent reports and/or protocols from the HCAI committee as a document here, or as a URL below.

Additional file

Additional file

Paste the URL here:

Additional URL

Additional URL

This space may be used to comment on the HCAI program (effectiveness or other clarifications)

Section Header: *Antimicrobial Stewardship (AMS)*

Is AMS a combined responsibility of the committee(s) described above?

*answer no if this is not a dedicated function of the committee(s) previously described or if there is a separate body responsible for AMS*

If yes, which committee is responsible for AMS

*e.g. the IPC committee, the combined IPC/HCAI committee*

Does your hospital have a formal AMS program?

Does your hospital provide fiscal and human resource support for maintaining the AMS program?

If yes, is there a budget line for this program?

What type of staff are involved in the program?

file

file

file

text

text

text

notes

yesno

1 Yes

1. No

text

yesno

1. Yes
2. No

yesno

1. Yes
2. No

yesno

1. Yes
2. No

checkbox

808

Show the field ONLY if: [ams_program] = '1'

[ ams_staff_other ]

Show the field ONLY if: [ams_staff(9)] = '1'

*check all that apply*

If you selected "other," please specify.

1

2

3

4

5

6

7

8

9

text

ams_staff 1

ams_staff 2

ams_staff 3

ams_staff 4

ams_staff 5

ams_staff 6

ams_staff 7

ams_staff 8

ams_staff 9

doctors nurses pharmacists

microbiologists environmental health administrators maintenance staff laboratory personnel other

809

[ ams_leader ]

Show the field ONLY if: [ams_program] = '1'

Is there a designated leader for the AMS program?

yesno

1. Yes
2. No

810

811

[ ams_leader_creds ]

Show the field ONLY if: [ams_leader] = '1'

[ ams_leader_compensation ]

If yes, what are the leader's credentials?

*(i.e. pediatric infectious disease doctor)*

How is the AMS program leader compensated for activities

text

checkbox

812

Show the field ONLY if: [ams_leader] = '1'

[ ams_compensation_other ]

Show the field ONLY if: [ams_leader_compensation(4)]

= '1'

related to this committee?

If you selected other, please specify.

1

2

3

4

text

ams_leader_compensation 1

ams_leader_compensation 2

ams_leader_compensation 3

ams_leader_compensation 4

additional salary support

relieved of other responsibilities so that they can lead the AMS program

AMS

responsibilities are added to their routine responsibilities without additional compensation

other

813

814

815

816

817

818

819

820

821

822

823

[ ams_antibiotic_monitor ]

[ ams_sop ]

[ ams_sop_attach ]

Show the field ONLY if: [ams_sop] = '1'

[ ams_sop_url ]

Show the field ONLY if: [ams_sop] = '1'

[ antibiotics_guidelines ]

[ antibiotics_national ]

Show the field ONLY if: [antibiotics_guidelines] = '1'

[ antibiotics_national_url ]

Show the field ONLY if: [antibiotics_guidelines] = '1'

[ antibiotics_hospital ]

Show the field ONLY if: [antibiotics_guidelines] = '1'

[ antibiotics_hospital_url ]

Show the field ONLY if: [antibiotics_guidelines] = '1'

[ ams_guidelines_adq ]

[ ams_effectiveness ]

Does the hospital monitor antibiotic use?

Is there an existing SOP on antibiotic use?

If available, please attach SOP on antibiotic use or enter URL below

Paste the URL here:

Are there formal guidelines available for the use of antibiotics in children?

If yes, please attach NATIONAL guidelines for the use of antibiotics in children or insert URL below.

Paste URL for national guidelines here

If yes, please attach HOSPITAL-SPECIFIC guidelines for the use of antibiotics in children or insert URL below.

Paste URL for hospital-specific guidelines here

Are the guidelines adequate to meet current needs?

This space may be used to comment on the AMS program (effectiveness or other clarifications).

yesno

1. Yes
2. No

yesno

1. Yes
2. No file

text

yesno

1. Yes
2. No file

text

file

text

yesno

1. Yes

0 No notes

824

825

826

827

828

829

830

831

832

833

834

835

836

[ quality_combined ]

Show the field ONLY if: [ipc_programs] = '1' or [hcai_pr ogram] = '1' or [ams_program]

= '1'

[ quality_combined_with ]

Show the field ONLY if: [quality_combined] = '1'

[ quality_program ]

Show the field ONLY if: [quality_combined] <> '1'

[ quality_program_attach_1 ]

Show the field ONLY if: [quality_program] = '1'

[ quality_program_attach_2 ]

Show the field ONLY if: [quality_program_attach_1] <> ''

[ quality_program_attach_3 ]

Show the field ONLY if: [quality_program_attach_2] <> ''

[ quality_program_ped ]

Show the field ONLY if: [quality_program] = '1'

[ quality_resources ]

Show the field ONLY if: [quality_program] = '1'

[ quality_budget ]

Show the field ONLY if: [quality_program] = '1'

[ quality_staff ]

Show the field ONLY if: [quality_program] = '1'

[ quality_staff_other ]

Show the field ONLY if: [quality_staff(5)] = 1

[ quality_leaders ]

Show the field ONLY if: [quality_program] = '1'

[ quality_leader_creds ]

Show the field ONLY if: [quality_leaders] = '1'

Section Header: *Quality Patient Safety*

Is patient safety a combined responsibility of the committee(s) described above?

*answer no if this is not a dedicated function of the committee(s) previously described or if there is a separate body responsible for quality patient safety*

If yes, which committee is responsible for quality patient safety

*e.g. the HCAI committee, the combined IPC/HCAI committee*

Do you have a program in quality patient safety?

If yes, please attach available guidelines or reports.

Additional file

Additional file

Do you have a quality patient safety program dedicated just to pediatric services?

Does the hospital provide fiscal and human resource support for maintaining the quality patient safety program?

If yes, is there a budget line for this program?

Who is involved in the quality patient safety program?

If you selected "other," please specify

Are there designated leaders for the quality patient safety program?

If yes, what are the leader's credentials?

*(i.e. hospital administrator)*

yesno

| 1 | Yes |
| --- | --- |
| 0 | No |

text

yesno

| 1 | Yes |
| --- | --- |
| 0 | No |

file

file

file

yesno

| 1 | Yes |
| --- | --- |
| 0 | No |

yesno

| 1 | Yes |
| --- | --- |
| 0 | No |

yesno

| 1 | Yes |
| --- | --- |
| 0 | No |

checkbox

| 1 | quality_staff 1 | doctors |
| --- | --- | --- |
| 2 | quality_staff 2 | nurses |
| 3 | quality_staff 3 | pharmacists |
| 4 | quality_staff 4 | administrators |
| 5 | quality_staff 5 | other |

text

yesno

| 1 | Yes |
| --- | --- |
| 0 | No |

text

| 837 | [ quality_leader_comp ]  Show the field ONLY if: [quality_leaders] = '1' | How is the quality patient safety program leader compensated for activities related to this committee? | checkbox | | | |
| --- | --- | --- | --- | --- | --- | --- |
|  |  |  | 1 | quality_leader_comp 1 | | additional salary support |
|  |  |  | 2 | quality_leader_comp 2 | | relieved of other responsibilities so that they can lead the quality patient safety program |
|  |  |  | 3 | quality_leader_comp 3 | | quality patient safety responsibilities are added to their routine responsibilities without additional compensation |
|  |  |  | 4 | quality_leader_comp 4 | | other |
| 838 | [ quality_comp_other ]  Show the field ONLY if: [quality_leader_comp(4)] = '1' | If you selected other, please specify. | text | | | |
| 839 | [ quality_certificate ] | Is there a requirement for healthcare providers to maintain a certification in patient safety? | yesno | |  | |
|  |  |  | 1 | Yes |  |  |
|  |  |  | 0 | No |  |  |
| 840 | [ quality_certificate_freq ]  Show the field ONLY if: [quality_certificate] = '1' | If yes, how frequently is recertification required? | text | | | |
| 841 | [ quality_certificate_onsite ]  Show the field ONLY if: [quality_certificate] = '1' | Do you provide training onsite? | yesno | |  | |
|  |  |  | 1 | Yes |  |  |
|  |  |  | 0 | No |  |  |
| 842 | [ adverse_events ] | Do you have a program to identify adverse events in the hospital? | yesno | |  | |
|  |  |  | 1 | Yes |  |  |
|  |  |  | 0 | No |  |  |
| 843 | [ adverse_events_report_1 ]  Show the field ONLY if: [adverse_events] = '1' | If yes, please attach a recent report. | file | | | |
| 844 | [ adverse_events_report_2 ]  Show the field ONLY if: [adverse_events_report_1] <> '' | Additional file | file | | | |
| 845 | [ adverse_events_report_3 ]  Show the field ONLY if: [adverse_events_report_2] <> '' | Additional file | file | | | |
| 846 | [ disinfection_protocols ] | Do you have protocols for disinfection of equipment, beds, etc.? | yesno | |  | |
|  |  |  | 1 | Yes |  |  |
|  |  |  | 0 | No |  |  |
| 847 | [ disinfection_protocols_1 ]  Show the field ONLY if: [disinfection_protocols] = '1' | If yes, please attach the protocol as a document or insert URL below | file | | | |
| 848 | [ adverse_events_url_1 ]  Show the field ONLY if: [disinfection_protocols] = '1' | Paste URL here | text | | | |
| 849 | [ disinfection_report ]  Show the field ONLY if: [disinfection_protocols] = '1' | If yes, please also attach a recent report. | file | | | |
| 850 | [ quality_effectiveness ] | This space may be used to comment on quality patient safety program (effectiveness or other clarifications). | notes | | | |
| 851 | [ workforcehs ] | Section Header: *Workforce Health and Safety*  Is there a committee on workforce health and safety/occupational health? | yesno | |  | |
|  |  |  | 1 | Yes |  |  |
|  |  |  | 0 | No |  |  |

852

853

854

855

856

857

858

859

860

[ worforce_mental_health ]

[ arv ]

[ arv_administered ]

Show the field ONLY if: [arv] = '1'

[ workforce_effectiveness ]

[ disaster_program ]

[ disaster_resources ]

Show the field ONLY if: [disaster_program] = '1'

[ disaster_budget ] Show the field ONLY if:

[disaster_resources] = '1' [ disaster_team ]

[ disaster_staff ]

Are mental health services available for staff members?

Is Antiretroviral (ARV) therapy available for staff members that may have been exposed to HIV?

If yes, how quickly can ARV be administered?

This space may be used to comment on the workforce health and safety program (effectiveness or other clarifications).

Section Header: *Disaster Response*

Does your facility have a disaster response operations program?

Does hospital provide fiscal and human resource support for maintaining the disaster response program?

If yes, is there a budget line for this program?

Do you have a disaster response team?

Who is involved in the disaster response operations team?

yesno

1 Yes

1. No

yesno

1. Yes
2. No text

notes

yesno

1. Yes
2. No

yesno

1. Yes
2. No

yesno

1. Yes
2. No

yesno

1. Yes
2. No

checkbox

861

Show the field ONLY if: [disaster_team] = '1'

[ disaster_staff_other ]

Show the field ONLY if: [disaster_staff(7)] = '1'

If you selected "other," please specify.

1

2

3

4

5

6

7

text

disaster_staff 1

disaster_staff 2

disaster_staff 3

disaster_staff 4

disaster_staff 5

disaster_staff 6

disaster_staff 7

doctors nurses pharmacists administrators

maintenance staff security

other

862

863

864

865

[ disaster_team_ped_rep ]

Show the field ONLY if: [disaster_team] = '1'

[ disaster_team_ped_creds ]

Show the field ONLY if: [disaster_team_ped_rep] = '1'

[ disaster_leader ]

Show the field ONLY if: [disaster_program] = '1'

[ disaster_leader_creds ]

Show the field ONLY if: [disaster_leader] = '1'

Does the disaster response team have pediatric representation?

If yes, please list credentials of pediatric representative(s) on disaster response team

Is there a designated leader for the disaster response operations program?

If yes, what are the leader's credentials?

*(i.e. hospital administrator)*

yesno

1. Yes
2. No text

yesno

1. Yes
2. No text

| 866 | [ disaster_leader_comp ] | How is the disaster response operations program leader | checkbox |  |
| --- | --- | --- | --- | --- |
| 867 | Show the field ONLY if: [disaster_leader] = '1'  [ disaster_compe_other ] Show the field ONLY if:  [disaster_leader_comp(4)] = '1' | compensated for activities related to this committee?  If you selected other, please specify. | 1. disaster_leader_comp 1 2. disaster_leader_comp 2 3. disaster_leader_comp 3 4. disaster_leader_comp 4   text | additional salary support  relieved of other responsibilities so that they can lead the disaster response operations program  disaster response responsibilities are added to their routine responsibilities without additional compensation  other |
| 868 | [ disaster_ministry_funct ] | Is disaster response coordination a Ministry function? | yesno | |

869

870

871

872

873

874

875

876

877

[ disaster_ministry_invol ]

Show the field ONLY if: [disaster_ministry_funct] = '1'

[ disaster_plan ]

[ disaster_plan_attach ]

Show the field ONLY if: [disaster_plan] = '1'

[ disaster_plan_url ]

Show the field ONLY if: [disaster_plan] = '1'

[ disaster_drills ]

[ last_3_drill ]

Show the field ONLY if: [disaster_drills] = '1'

[ disaster_drill_report_1 ]

Show the field ONLY if: [disaster_drills] = '1'

[ disaster_drill_report_2 ]

Show the field ONLY if: [disaster_drill_report_1] <> ''

[ disaster_drill_report_3 ]

Show the field ONLY if: [disaster_drill_report_2] <> ''

If yes, which Ministry or Ministries are responsible?

Does your facility have an disaster plan?

If yes, please attach disaster plan or insert URL below.

Paste URL here:

Do you run disaster simulations/drills?

If yes, what were the last 3 disaster drills you ran? (please indicate dates if possible)

Please attach a report if available.

Additional file

Additional file

1. Yes
2. No text

yesno

1. Yes
2. No file

text

yesno

1. Yes
2. No notes

file

file

file

| 878 | [ communication_methods ] | What type(s) of within-facility communication methods are available?  *check all that apply* | checkbox | | | | | |
| --- | --- | --- | --- | --- | --- | --- | --- | --- |
|  |  |  | 1 | communication_methods 1 | | | | hallway speakers |
|  |  |  | 2 | communication_methods 2 | | | | fire alarm |
|  |  |  | 3 | communication_methods 3 | | | | email (to entire staff) |
|  |  |  | 4 | communication_methods 4 | | | | beepers |
|  |  |  | 5 | communication_methods 5 | | | | mobile messaging group (e.g. whatsapp group) |
|  |  |  | 6 | communication_methods 6 | | | | staff are notified on an individual basis |
|  |  |  | 7 | communication_methods 7 | | | | other |
| 879 | [ comm_methods_other ]  Show the field ONLY if: [communication_methods(7)]  = '1' | If you selected "other," please specify. | text | | | | | |
| 880 | [ disaster_effectiveness ] | This space may be used to comment on the disaster response program (effectiveness or other clarifications). | notes | | | | | |
| 881 | [ equip_maintenance ] | Section Header: *Equipment Maintenance*  Do you have a program to repair and maintain medical equipment? | yesno | |  | | | |
|  |  |  | 1 | Yes |  |  |  |  |
|  |  |  | 0 | No |  |  |  |  |
| 882 | [ equip_maintenance_incl ]  Show the field ONLY if: [equip_maintenance] = '1' | Which of the following are maintained through these programs? | checkbox | | | | | |
|  |  |  | 1 | equip_maintenance_incl 1 | | | Ventilators and other ICU equipment | |
|  |  |  | 2 | equip_maintenance_incl 2 | | | Incubators | |
|  |  |  | 3 | equip_maintenance_incl 3 | | | Anesthesia equipment | |
|  |  |  | 4 | equip_maintenance_incl 4 | | | other | |
| 883 | [ equip_maint_other ]  Show the field ONLY if: [equip_maintenance_incl(4)] = '1' | If you selected "other," please specify | text | | | | | |
| 884 | [ equip_maint_effectiveness ] | This space may be used to comment on the equipment maintenance program (effectiveness or other clarifications). | notes | | | | | |
| 885 | [ programscommittees_comple te ] | Section Header: *Form Status*  Complete? | dropdown | | | | | |
|  |  |  | 0 | Incomplete | |  | | |
|  |  |  | 1 | Unverified | |  |  |  |
|  |  |  | 2 | Complete | |  |  |  |
| Instrument: Communication/Finances/Policy (communicationfinanceslegislation)  Enabled as survey | | | | | | | | |
| 886 | [ communication_backgd ] | Section Header: *Communication*  This is a space to give background on the reporting or surveillance systems in your facility/network and/or country | notes | | | | | |
| 887 | [ notifiable_diseases ] | Please attach a list of diseases/organisms that must be reported (i.e. list of notifiable diseases) or insert URL below | file | | | | | |
| 888 | [ notifiable_diseases_url ] | Paste URL here | text | | | | | |
| 889 | [ report_within ] | To whom are notifiable diseases reported within your hospital? (e.g. Director of Clinical Services) | text | | | | | |
| 890 | [ report_outside ] | To whom are notifiable diseases reported outside your hospital? (e.g. Ministry of Health, others) | text | | | | | |
| 891 | [ reporting_protocol ] | Please attach protocol for reporting or provide URL below | file | | | | | |
| 892 | [ reporting_protocol_url ] | Paste URL here | text | | | | | |

| 893 | [ other_outbreak_reports ] | Are there other infections/ outbreak situations where reporting to the Ministry is not required where you would report to your hospital leadership and/or the Ministry (e.g. a case or outbreak of listeriosis on a pediatric ward, outbreak of Necrotizing Enterocolitis in the NICU, etc.)? | yesno  1 Yes  0 No | | | | | |
| --- | --- | --- | --- | --- | --- | --- | --- | --- |
| 894 | [ other_reports_eg ]  Show the field ONLY if: [other_outbreak_reports] = '1' | If yes, please provide examples. | text | | | | | |
| 895 | [ other_report_within ]  Show the field ONLY if: [other_outbreak_reports] = '1' | If yes, to whom would this be reported within your hospital? (e.g. Director of Clinical Services) | text | | | | | |
| 896 | [ other_report_outside ]  Show the field ONLY if: [other_outbreak_reports] = '1' | If yes, to whom would this be reported outside your hospital? (e.g. Ministry of Health, others) | text | | | | | |
| 897 | [ other_reporting_protocol ]  Show the field ONLY if: [other_outbreak_reports] = '1' | Please attach a protocol for non-notifiable disease reporting (if different from notifiable disease reporting) | file | | | | | |
| 898 | [ syndrome_report ] | If patients come in with a common syndrome where an organism is not identified, is this reported? | yesno | |  | | | |
|  |  |  | 1 | Yes |  |  |  |  |
|  |  |  | 0 | No |  |  |  |  |
| 899 | [ syndrome_report_within ]  Show the field ONLY if: [syndrome_report] = '1' | If yes, to whom would this be reported within your hospital? (e.g. Director of Clinical Services) | text | | | | | |
| 900 | [ syndrome_report_outside ]  Show the field ONLY if: [syndrome_report] = '1' | If yes, to whom would this be reported outside your hospital? (e.g. Ministry of Health, others) | text | | | | | |
| 901 | [ syndrome_report_protocol ]  Show the field ONLY if: [syndrome_report] = '1' | Please attach a protocol for syndrome reporting (if different from notifiable disease reporting) | file | | | | | |
| 902 | [ send_moh_info ] | How do you send information to the Ministry of Health (MoH)?  *check all that apply* | checkbox | | | | | |
|  |  |  | 1 | send_moh_info 1 | | online portal | | |
|  |  |  | 2 | send_moh_info 2 | | telephone | | |
|  |  |  | 3 | send_moh_info 3 | | fax | | |
|  |  |  | 4 | send_moh_info 4 | | email | | |
|  |  |  | 5 | send_moh_info 5 | | mail | | |
|  |  |  | 6 | send_moh_info 6 | | automatic electronic reporting (e.g. certain results flagged in a database where patient test results are entered and sent automatically to MoH) | | |
|  |  |  | 7 | send_moh_info 7 | | other | | |
| 903 | [ send_moh_info_other ]  Show the field ONLY if: [send_moh_info(7)] = '1' | If you selected "other," please specify. | text | | | | | |
| 904 | [ risk_communication ] | Is risk communication to the public a hospital function or a Ministry function? (e.g. if there were a case of cholera, who would communicate this to the public?)  *check all that apply* | checkbox | | | | | |
|  |  |  | 1 | risk_communication 1 | | | Hospital function |  |
|  |  |  | 2 | risk_communication 2 | | | Ministry function |  |
|  |  |  | 3 | risk_communication 3 | | | Other |  |
| 905 | [ risk_communication_other ]  Show the field ONLY if: [risk_communication(3)] = '1' | If you selected "other," please specify. | text | | | | | |
| 906 | [ communication_clarify ] | This is a space to clarify any answers from the communication section | notes | | | | | |

907

908

909

910

911

912

913

914

[ medsys_paper_only ]

[ medsys_hybrid ]

[ medsys_electronic ]

[ scan_for_info ]

Show the field ONLY if: [medsys_hybrid] = '1' or [meds ys_electronic] = '1'

[ medical_records_comments ] [ total_budget ]

[ total_budget_peds ]

[ patient_costs ]

Section Header: *Medical Records*

Do you EXCLUSIVELY use a paper system for medical records?

Do you use a "hybrid system" for medical records (electronic and paper)?

Do you EXCLUSIVELY use an electronic system for medical records?

If you have an electronic medical record system, can you selectively search the system for specific information?

This is a space to comment on the medical records system.

Section Header: *Finances*

What is the total budget of your institution? (in local currency, please specify currency)

*leave blank if information is not available or decline to answer*

What is the total budget dedicated to pediatrics? (in local currency, please specify currency)

*leave blank if information is not available or decline to answer*

Are patients required to pay a co-pay or similar fee for any of the

yesno

1. Yes
2. No

yesno

1. Yes
2. No

yesno

1. Yes
2. No

yesno

1. Yes
2. No

notes text

text

checkbox

following:

*check all that apply*

1. patient_costs 1
2. patient_costs 2
3. patient_costs 3
4. patient_costs 4
5. patient_costs 5

inpatient hospital visits outpatient hospital visits lab tests

imaging medications

915

916

917

[ flat_fee ]

[ flat_fee_amt ]

Show the field ONLY if: [flat_fee] = '1'

[ flat_fee_exclusions ]

Do patients pay a flat fee for a hospital visit?

If yes, what is the fee?

*in local currency, please specify currency*

If yes, are any of the following NOT included in the flat fee?

yesno

1 Yes

1. No text

checkbox

918

Show the field ONLY if: [flat_fee] = '1'

[ flat_fee_exclusions_other ]

Show the field ONLY if: [flat_fee_exclusions(6)] = '1'

If you selected "other," please specify.

1

2

3

4

5

6

text

flat_fee_exclusions 1

flat_fee_exclusions 2

flat_fee_exclusions 3

flat_fee_exclusions 4

flat_fee_exclusions 5

flat_fee_exclusions 6

inpatient hospital visits outpatient hospital visits lab tests

imaging medications other

919

920

921

[ turn_away ]

[ turn_away_explain ]

Show the field ONLY if: [turn_away] = '1'

[ national_insurance ]

Do you ever have to turn away patients that are unable to pay?

If yes, please explain.

In your country, is there currently a program of national health insurance?

yesno

1. Yes
2. No notes

yesno

1. Yes

0 No

| 922 | [ national_insurance_plans ]  Show the field ONLY if: [national_insurance] = '0' | If no, are there plans for one? | yesno | |  | | | |
| --- | --- | --- | --- | --- | --- | --- | --- | --- |
|  |  |  | 1 | Yes |  |  |  |  |
|  |  |  | 0 | No |  |  |  |  |
| 923 | [ private_insurance_pct ] | What percent of your patient population has private insurance? | text (integer, Min: 0, Max: 100) | | | | | |
| 924 | [ money_from ] | Where does the hospital's money come from?  *check all that apply* | checkbox | | | | | |
|  |  |  | 1 | money_from 1 | | | national government |  |
|  |  |  | 2 | money_from 2 | | | patients |  |
|  |  |  | 3 | money_from 3 | | | private insurance |  |
|  |  |  | 4 | money_from 4 | | | national insurance |  |
|  |  |  | 5 | money_from 5 | | | donations/NGOs |  |
|  |  |  | 6 | money_from 6 | | | other |  |
| 925 | [ money_from_other ]  Show the field ONLY if: [money_from(6)] = '1' | If you selected "other," please specify. | text | | | | | |
| 926 | [ primary_fund_gov ]  Show the field ONLY if: [money_from(1)] = '1' | government | radio (Matrix - ranking)  1 Which is the primary source of funding? | | | | | |
| 927 | [ primary_fund_pat ]  Show the field ONLY if: [money_from(2)] = '1' | patients | radio (Matrix - ranking)  1 Which is the primary source of funding? | | | | | |
| 928 | [ primary_fund_priv ]  Show the field ONLY if: [money_from(3)] = '1' | private insurance | radio (Matrix - ranking)  1 Which is the primary source of funding? | | | | | |
| 929 | [ primary_fund_nat ]  Show the field ONLY if: [money_from(4)] = '1' | national insurance | radio (Matrix - ranking)  1 Which is the primary source of funding? | | | | | |
| 930 | [ primary_fund_don ]  Show the field ONLY if: [money_from(5)] = '1' | donations/NGOs | radio (Matrix - ranking)  1 Which is the primary source of funding? | | | | | |
| 931 | [ primary_fund_other ]  Show the field ONLY if: [money_from(6)] = '1' | other | radio (Matrix - ranking)  1 Which is the primary source of funding? | | | | | |
| 932 | [ how_drs_paid ] | How are doctors paid?  *check all that apply* | checkbox | | | | | |
|  |  |  | 1 | how_drs_paid 1 | | | Ministry of Health | |
|  |  |  | 2 | how_drs_paid 2 | | | academic institution/higher education | |
|  |  |  | 3 | how_drs_paid 3 | | | private practice | |
|  |  |  | 4 | how_drs_paid 4 | | | private insurance | |
|  |  |  | 5 | how_drs_paid 5 | | | national insurance | |
|  |  |  | 6 | how_drs_paid 6 | | | other | |
| 933 | [ how_drs_paid_other ]  Show the field ONLY if: [how_drs_paid(6)] = '1' | If you selected "other," please specify. | text | | | | | |
| 934 | [ disaster_funds ] | Does your institution have funds set aside for use in disasters? | yesno | |  | | | |
|  |  |  | 1 | Yes |  |  |  |  |
|  |  |  | 0 | No |  |  |  |  |
| 935 | [ disaster_funds_adq ]  Show the field ONLY if: [disaster_funds] = '1' | If yes, in the past, has the disaster fund been adequate for your needs? | radio | | | | | |
|  |  |  | 1 | Yes | |  | | |
|  |  |  | 0 | No | |  |  |  |
|  |  |  | 2 | Not Applicable | |  |  |  |

936

937

[ other_disaster_funds ]

Show the field ONLY if: [disaster_funds] = '0' or [disast er_funds_adq] = '0'

[ disaster_funds_where ]

Show the field ONLY if: [other_disaster_funds] = '1'

If there were no disaster funds set aside or disaster funds were inadequate, are there other places from which you have successfully obtained funding?

If yes, where?

radio

1 Yes

0 No

2 Not Applicable text

938

939

[ natl_disaster_fund ]

[ funds_ease_of_access ]

Is there a national disaster fund available?

If yes, how easy is it to access other national disaster funds?

yesno

1 Yes

1. No

radio

Show the field ONLY if: [natl_disaster_fund] = '1'

1. very easy
2. somewhat easy
3. somewhat difficult
4. very difficult
5. nearly impossible

940

941

942

943

944

945

946

947

948

949

950

[ annual_finances ]

[ annual_finances_attach ]

Show the field ONLY if: [annual_finances] = '1'

[ finances_clarify ] [ legal_policy ]

[ legal_policy_attach ]

Show the field ONLY if: [legal_policy] = '1'

[ legal_policy_attach_2 ]

Show the field ONLY if: [legal_policy_attach] <> ''

[ legal_policy_attach_3 ]

Show the field ONLY if: [legal_policy_attach_2] <> ''

[ legal_policy_url ]

Show the field ONLY if: [legal_policy] = '1'

[ legal_policy_url_2 ]

Show the field ONLY if: [legal_policy_url] <> ''

[ legal_policy_url_3 ]

Show the field ONLY if: [legal_policy_url_2] <> ''

[ consent_clarify ]

Do you have an annual financial statement?

Please attach annual financial statements/income expenditure statement if you are able.

This is a space to clarify any answers from the finances section

Section Header: *Consent for care*

Do you have a policy for treating children in the absence of a parent or guardian?

i.e. in the event that a child comes into the hospital and requires treatment but a parent or guardian cannot be found, are there legal protections that would allow doctors to treat the child? Is there someone in the hospital/government who can authorize treatment?

If yes, please attach policy for treating children in the absence of a parent or guardian or insert URL below

Additional File

Additional File

Paste consent policy URL here

Additional URL

Additional URL

This space may be used to comment on consent for care in your country/hospital.

yesno

1 Yes

0 No file

notes

yesno

1 Yes

0 No

file

file

file

text

text

text

notes

| 951 | [ communicationfinanceslegisl ation_complete ] | Section Header: *Form Status*  Complete? | | dropdown | | |
| --- | --- | --- | --- | --- | --- | --- |
|  |  |  |  | 0 | Incomplete |  |
|  |  |  |  | 1 | Unverified |  |
|  |  |  |  | 2 | Complete |  |
| Instrument: Summary and Suggestions (summary_and_suggestions) | | |  Enabled as survey |  |  |  |
| 952 | [ pp_name ] | Name, title, and contact information of person who filled out the survey: | | notes, Identifier | | |
| 953 | [ addl_people ] | Additional people who helped fill out the survey: title/job description/role in the organization, NO NAMES | | text | | |
| 954 | [ start_date ] | Internal evaluation date started | | text (date_dmy) | | |
| 955 | [ end_date ] | Internal evaluation date completed | | text (date_dmy) | | |
| 956 | [ hours_to_complete ] | Estimated time required to complete the survey (hours) | | text | | |
| 957 | [ suggestions ] | Thank you for taking the time to complete this survey. We value your feedback and would like to invite any suggestions you may have for improvements to the survey. | | notes | | |
| 958 | [ summary_and_suggestions_c omplete ] | Section Header: *Form Status*  Complete? | | dropdown | | |
|  |  |  |  | 0 | Incomplete |  |
|  |  |  |  | 1 | Unverified |  |
|  |  |  |  | 2 | Complete |  |
