# Supplementary material for: The Children’s Hospitals in Africa Mapping Project (CHAMP) survey: Facilities, equipment, supplies, infrastructure, and capacity to respond to emergencies
Source: PLOS Glob Public Health. 2025 Nov 26;5(11):e0005153. doi: 10.1371/journal.pgph.0005153 (PMC12654909; doi:10.1371/journal.pgph.0005153)
Supplement: S1 Table — (DOCX) [file pgph.0005153.s002.docx]

| **S1 Table: Nairobi Meeting Attendees** | | |
| --- | --- | --- |
| **Name** | **Hospital** | **Country** |
| Dr. Rodrick Kisenge | Muhumbili University of Health and Allied Sciences, Dar es Salaam | Tanzania |
| Dr. Workeabebe Abeba | Vice Chair, Department of Pediatrics and Child Health, Addis Ababa University | Ethiopia |
| Dr. Goitom Gebreyesuss Mebrahtu | Assistant Professor of Pediatrics and Child Health, Addis Ababa University | Ethiopia |
| Prof. Augustine Omoigberale | President of Paediatric Association of Nigeria | Nigeria |
| Dr. Nancy Ngum | New Partnership for Africa Development | South Africa |
| Prof. Martin Veller | University of Witwatersrand, Johannesburg | South Africa |
| Dr. Ashraf Coovadia | University of Witwatersrand, Johannesburg | South Africa |
| Dr. Heloise Buys | Head, Ambulatory & Emergency Paediatrics, Red Cross Memorial Children’s Hospital, Cape Town | South Africa |
| Dr. Anchen Laubscher | Group Medical Director, Queen Mamohato Memorial Hospital, Maseru | Lesotho |
| Mrs. Mathutsane Margaret Mohapi | General Manager, Tsepong Pty Ltd, Queen Mamohato Memorial Hospital, Maseru | Lesotho |
| Dr. Issa Makumbi | Assistant Commissioner (Epidemiology and surveillance Division), Ministry of Health | Uganda |
| Dr. Edison Arwanire Mworozi | Senior Consultant Paediatrician, Mulago National Referral Hospital, Kampala | Uganda |
| Dr. Ezekiel Mupere | Head, Dept. of Paediatrics, Makerere University, Kampala | Uganda |
| Dr. Pat Naidoo | Executive Director, ELMA Philanthropies Services East Africa Limited | Uganda |
| Dr. Aimable Kanyamuhunga | Head of Pediatrics Department & Child Health at Kigali University Teaching Hospital | Rwanda |
| Dr. Mandayachepa Nyando | Malawi University of Science and Technology, Thyolo | Malawi |
| Dr. Musaku Mwenechanya | University Teaching Hospitals-Children's Hospital, Lusaka, Zambia. | Zambia |
| Dr. Ebenezar Badoe | Head of Department of Child Health, Korle Bu Teaching Hospital, University of Ghana, Accra | Ghana |
| Dr. Sia Wata Camanor | Head Pediatrician & Chief Medical Officer, JFK Medical Center, Monrovia | Liberia |
| Dr. Crispen Ngwenya | Pediatrician, Parirenyatwa General Hospital, Harare | Zimbabwe |
| Dr. Magdalene Kuria | Pediatrician, Child Health and Mortality Prevention Surveillance, Kisumu | Kenya |
| Dr. Alliya Mohamed | Gertrude’s Children’s Hospital, Muthaiga, Nairobi | Kenya |
| Dr. Patrick Mburugu | Pediatrician, Jomo Kenyatta University, Juja | Kenya |
| Dr. Rosemarie Lopokoiyit | Secretary - Kenya Paeditrician Association | Kenya |
| Dr. Mildred Mudany | Pediatrician/Country Director - Jhpiego | Kenya |
| Prof. Ruth Nduati | Pediatrician/Faculty - Nairobi University | Kenya |
| Prof. Wilmot James | Visiting Professor of International and Public Affairs, Columbia University | USA |
| Prof. Phillip Larussa | Professor of Pediatrics - Division of Pediatric Infectious Diseases, Columbia University | USA |
| Prof. Lawrence Stanberry | Chairman of the Department of Pediatrics, Columbia University | USA |
| Ms. Hannah Grace Bousquet | Research Assistant, Columbia University | USA |
