# Supplementary material for: The Children’s Hospitals in Africa Mapping Project (CHAMP) survey: Facilities, equipment, supplies, infrastructure, and capacity to respond to emergencies
Source: PLOS Glob Public Health. 2025 Nov 26;5(11):e0005153. doi: 10.1371/journal.pgph.0005153 (PMC12654909; doi:10.1371/journal.pgph.0005153)
Supplement: S2 Table — (DOCX) [file pgph.0005153.s003.docx]

| **S2 Table: Adult ICU Capacity %(n/N)^a^** | |
| --- | --- |
| Hospitals have ICUs where children are cared for | 100 (20/20) |
| **Adult ICU** | |
| Number of Hospitals that have Adult ICUs | 30 (6/20) |
| Has paediatric beds in the ICU | 0 (0/6) |
| Average Bed Occupancy rate, median (IQR) | 57.5 (87.25) |
| Has adequate number of beds in the ICU to meet current paediatric needs | 0 (0/6) |
| Number of beds available for paediatric patients, median | 1.5 |
| Number of beds that are dedicated to paediatric patients | 0 |
| Average daily census of paediatric patients, range | (1-4) |
| Additional beds are needed for paediatric patients, median (IQR) | 9 (4.25) |
| Isolation rooms in the adult ICU that can be used for paediatric patients | 33 (2/6) |
| ^a^ n = positive responses and N = number of hospitals responding to survey questions | |
