# Supplementary material for: The Children’s Hospitals in Africa Mapping Project (CHAMP) survey: Facilities, equipment, supplies, infrastructure, and capacity to respond to emergencies
Source: PLOS Glob Public Health. 2025 Nov 26;5(11):e0005153. doi: 10.1371/journal.pgph.0005153 (PMC12654909; doi:10.1371/journal.pgph.0005153)
Supplement: S3 Table — (DOCX) [file pgph.0005153.s004.docx]

| **S3 Table: Combined NICU/ PICU Capacity % (n/N)^a^** | | |
| --- | --- | --- |
| Number of Hospitals that have a combined NICU/ PICU | 25 (5/20) | |
| Number of beds in the NICU/PICU, median (IQR) | 8 (5) | |
| Average daily census of paediatric patients, median (IQR) | 6 (2) | |
| Average Bed Occupancy rate (Median) (IQR) | 75 (22) | |
| Has adequate number of beds in the NICU/ PICU to meet current needs | 0 (0/5) | |
| Additional beds are needed for paediatric patients, median (IQR) | 7 (6) | |
| More beds needed specifically for bigger or smaller children | Bigger | 0 (0/5) |
|  | Smaller | 40 (2/5) |
|  | Both | 60 (3/5) |
| Reserved rooms in the Combined NICU/ PICU that can be used for neonatal patients | 60 (3/5) | |
| Number of beds reserved for neonatal patients are adequate for current needs | 0 (0/3) | |
| Has isolation rooms in the NICU/PICU | 40 (2/5) | |
| ^a^ n = positive responses and N = number of hospitals responding to survey questions | | |
