# Supplementary material for: The Children’s Hospitals in Africa Mapping Project (CHAMP) survey: Facilities, equipment, supplies, infrastructure, and capacity to respond to emergencies
Source: PLOS Glob Public Health. 2025 Nov 26;5(11):e0005153. doi: 10.1371/journal.pgph.0005153 (PMC12654909; doi:10.1371/journal.pgph.0005153)
Supplement: S4 Table — (DOCX) [file pgph.0005153.s005.docx]

| **S4 Table: Malnutrition Ward % (n/N)^a^** | |
| --- | --- |
| Number of Hospitals that have a Malnutrition Ward | 60 (12/20) |
| Number of beds in the Malnutrition Ward, median (IQR) | 15 (22.75) |
| Has adequate number of beds in the malnutrition ward to meet current needs | 25 (3/12) |
| Number of additional beds that are needed, median (IQR) | 13 (10) |
| Provides parenteral nutrition (IV) for malnourished patients | 26.3 (5/19) |
| Provides parenteral nutrition (IV) for other types of paediatric patients (e.g. short gut, gut failure, necrotizing enterocolitis, severe food allergy burnt patients, GI Disorders, Gastroschesis) | 40 (8/20) |
| Provides nutrition counseling for non-malnourished paediatric patients | 40 (8/20) |
| ^a^ n = positive responses and N = number of hospitals responding to survey questions | |
