# Supplementary material for: The Children’s Hospitals in Africa Mapping Project (CHAMP) survey: Facilities, equipment, supplies, infrastructure, and capacity to respond to emergencies
Source: PLOS Glob Public Health. 2025 Nov 26;5(11):e0005153. doi: 10.1371/journal.pgph.0005153 (PMC12654909; doi:10.1371/journal.pgph.0005153)
Supplement: S5 Table — (DOCX) [file pgph.0005153.s006.docx]

| **S5 Table: Reasons that prevent adding additional beds % (n/N)^a^** | | |
| --- | --- | --- |
| General paediatric wards | Staffing (nurses) | 67.8 (11/16) |
|  | Staffing (doctors) | 62.5 (10/16) |
|  | Finances | 75 (12/16) |
|  | Space | 81.3 (13/16) |
| PICU | Staffing (nurses) | 75 (9/12) |
|  | Staffing (doctors) | 66.7 (8/12) |
|  | Finances | 83.3(10/12) |
|  | Space | 66.7 (8/12) |
| NICU | Staffing (nurses) | 75 (9/12) |
|  | Staffing (doctors) | 83.3(10/12) |
|  | Finances | 83.3(10/12) |
|  | Space | 83.3(10/12) |
| Combined NICU/ PICU | Staffing (nurses) | 60 (3/5) |
|  | Staffing (doctors) | 40 (2/5) |
|  | Finances | 60 (3/5) |
|  | Space | 80 (4/5) |
| Adult ICU | Staffing (nurses) | 100 (6/6) |
|  | Staffing (doctors) | 83.3 (5/6) |
|  | Finances | 100 (6/6) |
|  | Space | 83.34 (5/6) |
| Malnutrition Ward | Staffing (nurses) | 33.34 (3/9) |
|  | Staffing (doctors) | 33.34 (3/9) |
|  | Finances | 55.56 (5/9) |
|  | Space | 100 (9/9) |
| ^a^ n = positive responses and N = number of hospitals responding to survey questions | | |
