# Supplementary material for: The Children’s Hospitals in Africa Mapping Project (CHAMP) survey: Facilities, equipment, supplies, infrastructure, and capacity to respond to emergencies
Source: PLOS Glob Public Health. 2025 Nov 26;5(11):e0005153. doi: 10.1371/journal.pgph.0005153 (PMC12654909; doi:10.1371/journal.pgph.0005153)
Supplement: S6 Table — (DOCX) [file pgph.0005153.s007.docx]

| **S6 Table: Disaster Response % (n/N)^a^** | | |
| --- | --- | --- |
| **Disaster Response Program** | | |
| Has a disaster response program | 52.6 (10/19) | |
| Has a designated leader for the program | 100 (10/10) | |
| Provides fiscal and human resource support for maintaining the program | 70 (7/10) | |
| Has a budget line for the program | 50 (5/10) | |
| **Disaster Response Team** | | |
| Has a disaster response team | 57.9 (11/19) | |
| Hospital staff that is a part of the disaster response team (860) | Doctors | 100 (11/11) |
|  | Nurses | 100 (11/11) |
|  | Pharmacists | 90.9 (10/11) |
|  | Administrators | 100 (11/11) |
|  | Maintenance Staff | 72.7 (8/11) |
|  | Security | 81.8 (9/11) |
| Has paediatric representation on the team | 81.8 (9/11) | |
| **Disaster Response Plan** | | |
| Has a disaster response plan | 57.9 (11/19) | |
| Runs disaster simulations and/or drills | 31.6 (6/19) | |
| Indicated that disaster response coordination is a Ministry level function | 78.9 (15/19) | |
| Methods of disaster communications | Hallway Speakers | 17.6 (3/17) |
|  | Fire Alarm | 52.9 (9/17) |
|  | Email (to entire staff) | 47.1 (8/17) |
|  | Beepers | 5.9 (1/17) |
|  | Mobile Messaging Group (e.g. WhatsApp) | 88.2 (15/17) |
|  | Staff are notified on an individual basis | 58.8 (10/17) |
| **Disaster Funding** | | |
| Has funds set aside for use in disasters | 16.7 (3/18) | |
| Can obtain funds for disasters from other places | 61.5 (8/13) | |
| Places funds can be obtained from | Within the hospital | 25 (2/8) |
|  | NGOs | 25 (2/8) |
|  | Ministry of Health | 25 (2/8) |
|  | Cooperating Partners | 12.5 (1/8) |
|  | Government Donors | 12.5 (1/8) |
| Has a National Disaster Fund | 14/16 (87.5) | |
| Accessing National Funds | Very Easy | 14.3 (2/14) |
|  | Somewhat Easy | 7.1 (1/14) |
|  | Somewhat Difficult | 42.9 (6/14) |
|  | Very Difficult | 7.1 (1/14) |
|  | Nearly Impossible | 28.6 (4/14) |
| ^a^ n = positive responses and N = number of hospitals responding to survey questions | | |
