# Supplementary material for: The Children’s Hospitals in Africa Mapping Project (CHAMP) survey: Facilities, equipment, supplies, infrastructure, and capacity to respond to emergencies
Source: PLOS Glob Public Health. 2025 Nov 26;5(11):e0005153. doi: 10.1371/journal.pgph.0005153 (PMC12654909; doi:10.1371/journal.pgph.0005153)
Supplement: S7 Table — (DOCX) [file pgph.0005153.s008.docx]

| **S7 Table: Anaesthetic Agents % (n/N)^a^** | | |
| --- | --- | --- |
| Methods by which reusable tools/equipment are sterilized | Autoclave | 100 (18/18) |
|  | Dry heat | 11.12 (2/18) |
|  | Radiation | 5.56 (1/18) |
|  | Chemically | 55.56 (10/18) |
| Methods that are available administration of anesthetic agents | Vaporizer | 82.35 (14/17) |
|  | Copper kettle | 0 (0/17) |
|  | Open drop | 0 (0/17) |
|  | By intravenous therapy(IV) | 100 (17/17) |
|  | By ether andchloroform drip | 11.76 (2/17) |
| Types of anesthetic agents that are available | Barbiturates | 76.47 (13/17) |
|  | Propofol | 70.59 (12/17) |
|  | Ketamine | 82.35 (14/17) |
|  | Dexmetatomidine | 47.06 (8/17) |
|  | Benzodiazepines | 58.82 (10/17) |
|  | Narcotics | 94.12 (16/17) |
|  | Succinylcholine | 76.47 (13/17) |
|  | Pancuronium | 52.94 (9/17) |
|  | Vecuronium | 47.06 (8/17) |
|  | Rocuronium | 23.52 (4/17) |
|  | Cisatracurium | 29.41 (5/17) |
|  | Atracurium | 35.94 (6/17) |
|  | Mivacurium | 11.76 (2/17) |
|  | Sevoflurane | 35.94 (6/17) |
|  | Halothane | 52.94 (9/17) |
|  | Desflurane | 35.94 (6/17) |
|  | Isoflurane | 47.06 (8/17) |
|  | Methoxyflurane | 23.52 (4/17) |
|  | Ether | 5.88 (1/17) |
|  | Chloroform | 11.76 (2/17) |
|  | Naloxone | 35.94 (6/17) |
|  | Sugammadex | 23.52 (4/17) |
|  | Flumazenil | 11.76 (2/17) |
| ^a^ n = positive responses and N = number of hospitals responding to survey questions | | |
