# Supplementary material for: The Children’s Hospitals in Africa Mapping Project (CHAMP) survey: Facilities, equipment, supplies, infrastructure, and capacity to respond to emergencies
Source: PLOS Glob Public Health. 2025 Nov 26;5(11):e0005153. doi: 10.1371/journal.pgph.0005153 (PMC12654909; doi:10.1371/journal.pgph.0005153)
Supplement: S8 Table — (DOCX) [file pgph.0005153.s009.docx]

| **S8 Table: Radiology Services % (n/N)^a^** | | |
| --- | --- | --- |
| Places where Radiological Services are available | Performed in your hospital | 95 (19/20) |
|  | Up-referred to another hospital in your network of hospitals | 35 (7/20) |
|  | up-referred to a hospital outside of your network of hospitals | 15 (3/20) |
| Radiological Services that are available | Ultrasound | 100 (20/20) |
|  | X-ray | 100 (20/20) |
|  | ComputedTomography scan (CT) | 70 (14/20) |
|  | Magnetic ResonanceImaging scan (MRI) | 50 (10/20) |
|  | Positron EmissionTomography (PET)scan | 5 (1/20) |
|  | Diagnostic nuclear medicine capabilities (e.g. bone, renal, or tagged white cell scan) | 25 (5/20) |
|  | Therapeutic Nuclear Medicine capabilities (e.g. radiotherapy for malignancies) | 25 (5/20) |
| Computed Tomography scan (CT) | up-referred to another hospital in your network of hospitals | 42.86 (3/7) |
|  | up-referred to a hospital outside of your network of hospitals | 57.14 (4/7) |
| Magnetic Resonance Imaging scan (MRI) | up-referred to another hospital in your network of hospitals | 40 (4/10) |
|  | up-referred to a hospital outside of your network of hospitals | 60 (6/10) |
| Positron Emission Tomography (PET)scan | up-referred to another hospital in your network of hospitals | 5.26 (1/19) |
|  | up-referred to a hospital outside of your network of hospitals | 31.58 (6/19) |
| Diagnostic nuclear medicine capabilities (e.g. bone, renal, or tagged white cell scan) | up-referred to another hospital in your network of hospitals | 0 (0/15) |
|  | up-referred to a hospital outside of your network of hospitals | 40 (6/15) |
| Therapeutic Nuclear Medicine capabilities(e.g. radiotherapy for malignancies) | Up-referred to another hospital in your network of hospitals | 20 (3/15) |
|  | up-referred to a hospital outside of your network of hospitals | 26.67 (4/15) |
| Has a linear accelerator on-site | 50 (2/4) | |
| Radiotherapy is provided in your hospital/network | 50 (10/20) | |
| Has a blood irradiator in your hospital/network (342) | 16.67 (3/18) | |
| ^a^ n = positive responses and N = number of hospitals responding to survey questions | | |
