# Supplementary material for: The Children’s Hospitals in Africa Mapping Project (CHAMP) survey: Facilities, equipment, supplies, infrastructure, and capacity to respond to emergencies
Source: PLOS Glob Public Health. 2025 Nov 26;5(11):e0005153. doi: 10.1371/journal.pgph.0005153 (PMC12654909; doi:10.1371/journal.pgph.0005153)
Supplement: S9 Table — (DOCX) [file pgph.0005153.s010.docx]

| **S9 Table: Combined NICU/ PICU Equipment and Supplies** | |
| --- | --- |
| **Available Equipment, Supplies or Procedures** | **% (n/N)^a^** |
| Pulse oximeters | 100 (5/5) |
| Continuous ECG monitors | 60 (3/5) |
| Invasive pressure monitors | 60 (3/5) |
| Mechanical ventilators | 80 (4/5) |
| CPAP machines | 100 (5/5) |
| Oxygen concentrators | 0 (0/5) |
| Oxygen cylinders | 100 (5/5) |
| Peritoneal dialysis | 80 (4/5) |
| Haemodialysis machine | 40 (2/5) |
| Cooling devices for induced hypothermia | 40 (2/5) |
| High frequency oscillators (HFVO) | 40 (2/5) |
| EEG monitors | 20 (1/5) |
| Ultrasound machines | 80 (4/5) |
| Phototherapy lights | 100 (5/5) |
| Central line insertion and maintenance | 60 (3/5) |
| **Number of Machines** | **Median (IQR)** |
| Number of mechanical ventilators | Not enough data |
| Number of functional mechanical ventilators | Not enough data |
| Number of CPAP machines | 4 (2) |
| Number of functional CPAPs machines | 4 (3) |
| Number of Oxygen Concentrators in the ICU | No data |
| Number of Oxygen Cylinders in the ICU | No data |
| Number of functional Oxygen Concentrators in the ICU | Not enough data |
| ^a^ n = positive responses and N = number of hospitals responding to survey questions | |
