# Supplementary material for: The Children’s Hospitals in Africa Mapping Project (CHAMP) survey: Facilities, equipment, supplies, infrastructure, and capacity to respond to emergencies
Source: PLOS Glob Public Health. 2025 Nov 26;5(11):e0005153. doi: 10.1371/journal.pgph.0005153 (PMC12654909; doi:10.1371/journal.pgph.0005153)
Supplement: S10 Table — (DOCX) [file pgph.0005153.s011.docx]

| **S10 Table: Medical Supplies % (n/N)^a^** | | |
| --- | --- | --- |
| Has adequate medical supplies to meet current needs | | 31.6 (6/19) |
| Re-use disposable supplies | | 31.6 (6/19) |
| Medical supplies that are prone to shortages | Sterile surgical gloves | 52.6 (10/19) |
|  | Saline | 21.1 (4/19) |
|  | Recommended paediatric vaccines | 21.1 (4/19) |
|  | Commonly used antibiotics (penicillin, ampicillin, vancomycin, nafcillin, gentamycin, amikacin, cephalothin, cefotaxime, etc.) | 47.4 (9/19) |
|  | Insulin | 31.6 (6/19) |
|  | Anti-malarial drugs | 26.3 (5/19) |
|  | Oxygen | 42.1 (8/19) |
|  | Syringes and/or needles appropriate for paediatric use | 26.3 (5/19) |
|  | Endotracheal tubes appropriate for infants and children | 63.1(12/19) |
| Has experienced supply shortages during periods of extreme heat | | 27.8 (5/18) |
| Medical supplies that experience a shortage because of extreme heat | Gloves | 60 (3/5) |
|  | I.V Catheters | 60 (3/5) |
|  | I.V Fluids | 80 (4/5) |
|  | Other | 60 (3/5) |
| ^a^ n = positive responses and N = number of hospitals responding to survey questions | | |
