# Supplementary material for: The Children’s Hospitals in Africa Mapping Project (CHAMP) survey: Facilities, equipment, supplies, infrastructure, and capacity to respond to emergencies
Source: PLOS Glob Public Health. 2025 Nov 26;5(11):e0005153. doi: 10.1371/journal.pgph.0005153 (PMC12654909; doi:10.1371/journal.pgph.0005153)
Supplement: Attachment — Submitted filename: Response to Reviewers.docx [file pgph.0005153.s015.docx]

PGPH-D-25-01272
The Children’s Hospitals in Africa Mapping Project (CHAMP) Survey: Facilities, Equipment, Supplies, Infrastructure, and Capacity to Respond to Emergencies.
PLOS Global Public Health


Dear Reviewers,

Please see our responses to the questions and comments raised.

**2. Has the statistical analysis been performed appropriately and rigorously?**

Reviewer #1: Yes

Reviewer #2: No

We reviewed all analyses in the paper to detect any errors or misuse of methods and were unable to pick up any flaws. It would have been helpful to have specific comments on what was not performed appropriately.

**Review Comments to the Author**

**Reviewer #1: The 'survey development' section should be described in more detail: please define 'healthcare leaders' and 'team from Columbia'. How were the 13 African countries selected? Were the healthcare leaders compensated or incentivized for their contributions to the survey? Additionally, how were the surveyed hospitals selected? Purposive sampling, snowball technique...?**

In August 2018, four members of Columbia University’s Department of Pediatrics (S1 Table) convened a two-day meeting at Columbia Global Center in Nairobi with 25 individuals from 13 African countries who held hospital, medical school or healthcare-related leadership roles (S1 Table). Those invited to the meeting were known to the Columbia team or were identified through snowball recruitment by those known to the Columbia team. The meeting was convened to discuss the development of a survey to assess the readiness of hospitals in sub-Saharan Africa to respond to critical events such as epidemic outbreaks and mass casualties affecting children. The healthcare leaders were not compensated or incentivized to contribute and did so willingly on the basis that such data at each site could be used for their own local advocacy and audit purposes. This is included in the revised manuscript.

The hospitals included were those that met the criteria and that the healthcare leaders who attended the Nairobi meeting had access to. As they all came from large institutions within their countries this was representative of the highest levels of care for children in those countries albeit such a selected few. This is now made clearer in the limitations section.

**The overall goal of the survey seems to conflict itself at times: initially it is described as an assessment of pediatric readiness during mass casualties and epidemic outbreaks. However, later the participants expanded the scope of the survey to include quite broad measures, like "highest quality of care" and "malnutrition support services." If the scope was just overall pediatric readiness, and not specifically emergencies or mass casualties, the title and abstract should be changed to reflect this.**

The initial goal of the Columbia team was to focus on readiness but that the African leaders attending the meeting wanted a much broader goal that is reflected in the title and abstract. These would still be part of an emergency response audit as undoubtedly the number of cases of malnutrition would escalate in such instances. We believe the title still captures the essence of this study despite having a few additional areas captured in the data.

**Please give more information about the IRB process, including the five sites where review was required and protocol number. In line 144, define "CIUMC".**

Five hospitals conducted their standard IRB review and deemed that the study did not require IRB oversight. When the study was designed the investigators were assured that the data would be aggregated and de-identified so that no site could be identified. We do not think it appropriate to identify the five hospitals that conducted IRB reviews.

Columbia University Irving Medical Center (CUIMC) was defined in the section above under site selection and survey implementation.

**Line 184 seems irrelevant to this study's aims of emergency preparedness: "The most common causes of high monthly admissions were malaria, respiratory tract infections, diarrheal diseases, meningitis, and malnutrition." Lines 228-230 is similar, where if you're talking about emergency preparedness, I don't know that high daily volumes from pneumonia is applicable. Table 6 is similar "most common reasons for high ER usage": if you're trying to connect common diagnoses as potential areas for offloading during a surge, you need to explicitly state that. Otherwise this data seems like it belongs in a different publication.**

We apologize that it is clear that the main focus was on the hospitals' capacity to provide highest quality care not not only emergency care. These questions relate the most common illnesses the hospitals face and that might impact their ability to provide the highest level of care.

**In Table 2, define "SOP."**

Thisabbreviation has been replaced by ‘protocol’ which we believe is clearer.

**Line 205 has a typo: " For the hospitals with separate NICUs, the average number of beds was 17. 5 (range, 4-50)
the average daily census was 26, with an average bed occupancy of 100% (Table 4)."**

Thank you we have fixed that error in the revised manuscript.

**Table 7: I don't see how average wait time to elective surgery pertains to mass casualties and emergency preparedness.**

This piece of data was considered relevant as it was aimed at showing what these hospitals face in providing the highest quality of care during times outside of a disaster. Whilst it is not directly related to emergency preparedness it does provide good information, we believe, on the hospital’s capacity in this area that provided another metric of comparison with other hospitals on the continent.

**Line 358 has two periods.**

This is corrected in the revised manuscript.

**Line 438-439: "Not only is it the right thing to do, but it is good economics." This is an important point that needs to be much more clearly emphasized. Give statistics on how much money is saved by investing in quality pediatric care.**

While the specific survey responses did not allow a detailed examination of cost-savings and return on investment due to investments in improved pediatric care, the survey data (available open-source) will allow future studies to expand on the economic impacts of these investments.

A brief illustrative summary of literature findings on investments in improved quality of pediatric care in hospital in Sub-Saharan settings is provided below. We have added these references to the manuscript in support of the statement in line 438-439.

| **Intervention / Area** | **Location** | **Intervention Description** | **ROI / Cost‑Effectiveness Highlights** | **Reference** |
| --- | --- | --- | --- | --- |
| WHO Emergency Care Toolkit | Uganda | Implementation of WHO’s Emergency Care Toolkit at regional referral hospitals to improve emergency triage, protocols, and training. | 655% ROI; US $1.67M saved; 34 lives saved in 1 year; scale-up adds US $29.9M in economic gains | Werner, K., Risko, N., Kalanzi, J., Wallis, L. A., & Reynolds, T. A. (2022). Cost-effectiveness analysis of the multistrategy WHO emergency care toolkit in regional referral hospitals in Uganda. *PLoS ONE*, *17*(12 October). |
| Smart Triage Pediatric Platform | Uganda (Jinja Hospital) | Deployment of a digital triage platform for rapid identification and management of pediatric sepsis. | US $15.32 saved per YLL averted; >94% probability of being cost-saving | Li, E. C. K., Tagoola, A., Komugisha, C., Nabweteme, A. M., Pillay, Y., Ansermino, J. M., & Khowaja, A. R. (2023). Cost-effectiveness analysis of Smart Triage, a data-driven pediatric sepsis triage platform in Eastern Uganda. *BMC Health Services Research*, *23*(1). https://doi.org/10.1186/s12913-023-09977-5 |
| Pediatric Operating Rooms | Sub-Saharan Africa | Establishment of two pediatric operating rooms to improve access to emergency surgical care. | US $151 per DALY averted; cost-effective surgical preparedness investment | Yap, A., Halid, S. I., Ukwu, N., Laverde, R., Park, P., Klazura, G., Bryce, E., Cheung, M., Marseille, E., Ozgediz, D., & Ameh, E. A. (2023). *Cost-Effectiveness of a Pediatric Operating Room Installation in Sub-Saharan Africa*. https://doi.org/10.1101/2023.03.02.23286697 |
| Community Health Worker (CHW) Programs | sub-Saharan Africa | Expansion of CHW programs for home-based care, referrals, and health education for maternal and child health. | 10:1 ROI; improved child survival, jobs, and system efficiencies | Masis, L., Gichaga, A., Zerayacob, T., Lu, C., & Perry, H. B. (2021). Community health workers at the dawn of a new era: 4. Programme financing. In *Health Research Policy and Systems* (Vol. 19). BioMed Central Ltd. https://doi.org/10.1186/s12961-021-00751-9 |
| Maternal & Child Health Investment | sub-Saharan Africa | Investments in core maternal, newborn, and child health services including immunization, nutrition, and obstetric care. | US $7 return per US $1 invested; global economic gains up to US $3.8 trillion by 2035 | A Adotey, S. K. (n.d.). *investing-in-maternal-and-child-health-fuels-prosperity-for-women-young-people-and-children-in-africa/ 2/11*. https://www.weforum.org/stories/2024/09/how |
| Regional Emergency Preparedness Program | Ethiopia, Kenya, São Tomé & Príncipe | World Bank-financed multisectoral program to enhance epidemic surveillance, labs, and emergency response capacity. | BCR 5.2; IRR 29.5%; positive NPV across preparedness investments | World Bank (2023): https://documents1.worldbank.org/curated/en/099433310162397541/pdf/SECBOS00d3ae4a090af870e8bab78dc000.pdf |

**In the limitations section, you state that not all hospitals answered every question. Please include response rates by percentages - the questions that are left unanswered also yield important data.**

All the tables had quantitative data in the following format % (n/N)^a^ where a n = positive responses and N = number of hospitals responding to survey questions. So we believe we have addressed this issue.

**Reviewer #2: The title addresses a relevant and important topic, particularly for pediatric healthcare in sub-Saharan Africa. The following comments are intended to help strengthen the manuscript’s quality, clarity, and scientific rigor.**

**Major Comments**
**1. Sample Size and Selection Criteria**
**The study does not clearly describe how the 20 hospitals were selected by the participants in Nairobi. The note that “Potential eligible sites were suggested by the Nairobi meeting participants” requires further explanation — detailing how, when, and based on what criteria these sites were proposed, and whether this process introduced selection bias.**

We describe in the Site selection and survey implementation section

our criteria for selection of the hospitals met the CHAMP definition of a “Children’s Hospital”.

In the Survey Development section we designated. "a CHAMPS “Children’s Hospital”, as facilities, either hospital building based, or ward based within hospitals, that focus on providing care to neonates, children and adolescents and are capable of providing a broad array of paediatric medical and surgical services, and are staffed by physicians and nurses dedicated to paediatric care with variable degree of formal paediatric training. We recruited 24 hospitals based on funding available. As noted by the reviewer and stated in Hospital characteristics section, 19 of 20 of the hospitals were affiliated with medical schools. This was intentional as teaching hospitals are generally the best staffed and resourced hospitals. As pointed out in the Discussion section, "Given their status, these institutions would be among the leading children’s hospitals in their country”. We intentionally surveyed what we thought would be among the best staffed and resourced children's hospitals showcasing the capabilities, limitations and challenges of the top programs. We recognized that this group of hospitals were not representative of most hospitals where children might receive care. Indeed, we expect that most hospitals where children might receive care have greater challenges and fewer resources than the hospitals in our study. In the Limitations section, we acknowledge that our group of hospitals are not representative of the broader landscape of hospitals in Africa.

Invitations to participate in the CHAMP project were emailed to pediatric chiefs at institutions that met the criteria in the last quarter of 2018.

**2. Clarification of Terminology
The term “Children’s Hospital” may give the impression that Africa has many stand-alone pediatric hospitals, which is not the case in most countries. The authors should try another wording than children hospital as it gives wrong impression that these hospitals were standalone pediatric hospitals even if it is operational defined**

We believe we did address this in the Survey development section (lines 116-122).

“While the term “Children’s Hospital” may imply a stand-alone facility that exclusively cares for children, during the meeting, the group developed a more practical designation of a CHAMP “Children’s Hospital”, as facilities, either hospital building based, or ward based within hospitals, that focus on providing care to neonates, children and adolescents and are capable of providing a broad array of paediatric medical and surgical services, and are staffed by physicians and nurses dedicated to paediatric care with variable degree of formal paediatric training”

3. Use of WHO Standards
**The criteria used to classify medical supplies and services as “adequate” or “inadequate” are not clearly defined. It would strengthen the methodology if the authors referenced established international standards — such as the WHO Hospital Emergency Response Checklist or the Essential Emergency and Critical Care (EECC) framework — to guide and standardize the hospital readiness assessment.**

We acknowledge this fine point and will make reference to these standards in the conclusions as they would certainly assist in the design of future studies in this area. We believe in will strengthen this section. These two references below will be added to the revised manuscript.

**1.** World Health Organization Regional Office for Europe. Hospital emergency response checklist: an all-hazards tool for hospital administrators and emergency managers. Copenhagen: WHO Regional Office for Europe; 2011. Available from: <https://www.who.int/publications/i/item/hospital-emergency-response-checklist>

**2.** Schell CO, Gerdin Wärnberg M, Hvarfner A, Höög A, Baker U, Castegren M, et al. Essential emergency and critical care: a consensus among global clinical experts. BMJ Glob Health. 2021;6(9):e006585. doi:10.1136/bmjgh-2021-006585. Available from: <https://gh.bmj.com/content/6/9/e006585>

**4. Results Section
The sentence “The median ‘maximum age’ of children cared for in the facilities surveyed was 14 years (range: 12–18)” (line 176) requires clarification. It is unclear whether this refers to the maximum age allowed for admission, or the median of the maximum ages reported by each hospital. Both the maximum and median values should be clearly defined and reported consistently.**

Nineteen of twenty hospitals surveyed reported the maximum age of children that were allowed for admission in their facilities. The median maximum age allowed for admission was 14 (range 12-18). We have rephrased this sentence in the revised manuscript.

**5. Discussion Section
The discussion section largely restates the results without critically interpreting them in relation to the study’s aim — assessing hospitals' readiness to care for children amid public health emergencies.
Additionally:
The data point that “Fourteen out of 20 hospitals (70%) reported that the wait time in the emergency area was less than one hour” should be discussed more meaningfully. This reported wait time is considerably better than expected in the African context, given known challenges with patient load, staff shortages, and emergency system limitations. The authors should explore possible explanations and discuss this finding within the regional context.**

We agree this did seem better than expected given the resource constraints. This survey did not have a follow up with the respondents to delve into specifics such as these and therefore the possible explanations at this stage would be speculative. So whilst we agree this would be have useful in the discussion we feel there isn’t enough data from the respondents to offer an explanation for this.

**Although the aim mentions readiness for emergencies and disasters, the findings are not adequately discussed within the framework of emergency and disaster preparedness standards. The authors should reflect on how the reported infrastructure, equipment, and services compare to established emergency and disaster readiness benchmarks.**

While our survey did not utilize the WHO emergency response checklist, the survey questions did address the domains contained in the checklist. As stated above we will now include the relevant references in the conclusion section.

**6. Conclusion (in both Abstract and Main Text)
Critical findings such as:
λThe necessity of admitting more than one child in a bed.
λThe infection risks associated with placing multiple children in one crib
λFrequent interruptions in water supply
should be explicitly highlighted, as these have significant implications for hospital readiness, infection control, and patient safety.**

Thank you these are now incorporated into the conclusion in the revised manuscript.

**7. Study Limitations
The limitations of the study should be explicitly stated, including:
λThe nature of the hospitals surveyed (mostly tertiary, teaching hospitals)
λThe sample’s lack of representative for the wider African hospital landscape
λPotential selection bias**

These limitations are now included in the revised manuscript.

**Editorial Comments
The aim of the study is unnecessarily repeated between lines 91–96 and again in lines 97–98. This duplication should be removed for clarity.**

This redundancy is addressed now in the revised manuscript.

**Key words: can be focused to the research**

Agree and this will be the revised list of key words below.

pediatric hospitals; emergency preparedness; hospital infrastructure; Health Systems; disaster readiness; hospital capacity; inpatient services; healthcare resources; health equity; hospital surge capacity; healthcare delivery; public health emergencies

Finally our revised manuscript has now got a revised reference list with an additional 6 references.

Sincerely

Ashraf Coovadia

Corresponding Author
