# Supplementary material for: The Children’s Hospitals in Africa Mapping Project (CHAMP) survey: Facilities, equipment, supplies, infrastructure, and capacity to respond to emergencies
Source: PLOS Glob Public Health. 2025 Nov 26;5(11):e0005153. doi: 10.1371/journal.pgph.0005153 (PMC12654909; doi:10.1371/journal.pgph.0005153)
Supplement: Attachment — Submitted filename: Response to Reviewers 2 October 2025 .docx [file pgph.0005153.s016.docx]

2 October 2025

PGPH-D-25-01272
The Children’s Hospitals in Africa Mapping Project (CHAMP) Survey: Facilities, Equipment, Supplies, Infrastructure, and Capacity to Respond to Emergencies.
PLOS Global Public Health

Vinay Nair Kampalath, MD, MSc, DTM&H
Academic Editor
PLOS Global Public Health

Dear Dr Kampalath,

We would like to thank the reviewers for their insightful comments and for the opportunity to revise our manuscript. We have carefully addressed all concerns raised and have made the necessary corrections as outlined below:

**Reviewer #1**

**Comment 1:**
*Line 74 should contain two separate sentences.*
**Response:**
Corrected. The sentence now reads: *“Twenty-four sites were recruited. Twenty hospitals from 15 countries completed the survey from 2018 to 2019.”*

**Comment 2:**
*Line 86 should not have a comma after "where".*
**Response:**
The comma after “where” has been removed.

**Comment 3:**
*Line 100 should not have a comma after "Tarun".*
**Response:**
The comma after “Tarun” has been removed.

**Comment 4:**
*Line 161 should have a space between "Sierra" and "Leone".*
**Response:**
This spacing error has been corrected.

**Reviewer #2**

**Comment 1:**
*The manuscript still requires English language editing.*
**Response:**
We have edited the manuscript to improve grammar, clarity, and overall readability particularly in the discussion and conclusion sections. In this process we also found the percentage data missing for "commonly used antibiotics" in line 345 which we have filled in now.

**Comment 2:**
*The reference style (e.g., references 17–23) may need summarizing.*
**Response:**
We have reviewed ALL the references and not just those in question and summarized them where appropriate, in line with the journal's referencing style. We have therefore reformatted the entire reference list as we did pick up formatting inconsistencies.

We used an AI tool to assist with formatting references  in Vancouver style to ensure consistency and accuracy. No AI tools were used in the preparation of the manuscript beyond formatting the reference list.

**Comment 3:**
*In line 198, the statement that all hospitals have a PICU contradicts the discussion regarding a shortage of PICUs.*
**Response:**
It is correct that all hospitals had a PICU but we can find nowhere in the manuscript where we describe a shortage of pediatric intensive care units. We did describe a shortage of PICU beds. Perhaps the reviewer was confused about units versus beds.

**Comment 4:**
*The conclusion could be improved and better structured.*
**Response:**
We have revised the conclusion to ensure it is concise and focused. Redundant elements have been moved to the discussion, and we have included incorporated the study limitation in the discussion section now and removed the section labelled as ‘Limitations’

We sincerely appreciate the reviewers' constructive feedback, which has significantly improved our manuscript. We hope the revised version meets the standards for publication.

Sincerely

Ashraf Coovadia

Corresponding Author
